# Dynamics of genetic and somatic trade-offs in ageing and mortality

**DOI:** 10.1038/s41586-026-10407-9

**Published:** 2026-04-22

**Authors:** Danny Arends, David G. Ashbrook, Suheeta Roy, Lu Lu, Zachary Sloan, Arthur G. Centeno, Kurt H. Lamour, João Pedro de Magalhães, Pjotr Prins, Karl W. Broman, Saunak Sen, Sarah J. Mitchell, Michael R. MacArthur, Özlem Altintas Akin, Xiaoxu Li, Amandeep Bajwa, Vivian Diaz, David E. Harrison, Randy Strong, James F. Nelson, Khyobeni Mozhui, Johan Auwerx, Evan G. Williams, Richard A. Miller, Robert W. Williams

**Affiliations:** 1 Department of Applied Sciences, Northumbria University, Newcastle upon Tyne, UK; 2 Department of Genetics, Genomics and Informatics, University of Tennessee Health Science Center, Memphis, TN USA; 3 Institute for Neuroscience and Cardiovascular Research, The University of Edinburgh, Edinburgh, UK; 4 Department of Entomology and Plant Pathology, University of Tennessee Knoxville, Knoxville, TN USA; 5 Department of Inflammation and Ageing, University of Birmingham, Birmingham, UK; 6 Department of Biostatistics and Medical Informatics, University of Wisconsin-Madison, Madison, WI USA; 7 Department of Preventive Medicine, University of Tennessee Health Science Center, Memphis, TN USA; 8 Ludwig Princeton Branch, Princeton University, Princeton, NJ USA; 9 Lewis-Sigler Institute for Integrative Genomics, Princeton University, Princeton, NJ USA; 10 Department of Health Sciences and Technology, ETH Zurich, Zurich, Switzerland; 11 Laboratory of Integrative Systems Physiology, Institute of Bioengineering, École Polytechnique Fédérale de Lausanne, Lausanne, Switzerland; 12 Department of Surgery, University of Tennessee Health Science Center, Memphis, TN USA; 13 Barshop Institute for Longevity and Aging Studies, University of Texas Health San Antonio, San Antonio, TX USA; 14 The Jackson Laboratory, Bar Harbor, ME USA; 15 Luxembourg Centre for Systems Biomedicine, University of Luxembourg, Esch-sur-Alzette, Luxembourg; 16 Department of Pathology and Geriatrics Center, University of Michigan, Ann Arbor, MI USA

**Keywords:** Genetic linkage study, Biomarkers, Genetic variation

## Abstract

DNA variants modulate mortality risks across an entire lifespan but their dynamic age-dependent effects have not been resolved in any species for either sex. Here we mapped variants that shape mortality using an actuarial approach, starting with a base population of 6,438 pubescent mice and ending with 559 survivors that lived beyond 1,100 days of age. Twenty-nine *Vita* loci influence lifespan with strong age- and sex-specific effects. Most act during distinct stages with polarities that often invert with age, but a minority have consistent age-dependent effects in one or both sexes. A separate set of 30 *Soma* loci influence correlations between body mass and life expectancy. Nineteen *Soma* loci mediate higher mortality in larger young mice, whereas 11 mediate lower mortality in larger old mice. All effects are stronger in male mice than in female mice. *Vita* and *Soma* loci form epistatic networks split strictly by sex. These findings provide a genetic bridge between evolutionary theories of ageing and molecular mechanisms that can guide interventions to extend healthy lifespan.

## Abstract (web summary)

Mapping DNA variants that modulate mortality using a starting population of 6,438 young mice defined 29 distinct loci that influence lifespan and mortality with divergent age- and sex-specific effects, as well as 30 loci that specifically couple body mass with longevity.

## Main

We do not yet understand the genetic, molecular, cellular and organismal processes that shape the intrinsic variability in rates of ageing, lifespan and all-cause mortality in humans, mice or other model organisms^1–11^. Although thousands of variants modulate risks of age-related diseases, the great majority influence proximate causes of death and not the core mechanisms that influence ageing rates per se^12,13^. To disentangle causes from consequences and to define those variants that modulate mortality across entire lifespans, we developed actuarial methods that map variation in lifespan of progressively older survivorships—equivalent to a range from 12 to 94 years in humans^14^. This approach has been tested^4,15–17^, but has not been applied systematically in any organism. The closest human parallels are biometric studies of age-dependent changes in heritability of lifespan in Scandinavian twin cohorts born between 1870 and 1910^18^. Our study is a complementary dissection resolved at the level of discrete genetic effects—59 well-defined loci that dynamically interact to influence mortality rates and lifespan. We address four sets of questions in geroscience:

1. What DNA variants influence mortality rates, and how and when do they act? Which have persistent effects, which have transient effects, and which act only late in life?
2. How do these loci align with sex differences in mortality during and after reproduction? Is there evidence of genotype-by-sex (G × S) interactions, genotype-by-genotype (G × G) interactions, or even genetic antagonism between the sexes?
3. What loci account for the strong negative association between larger body size early in life and shorter lifespans versus larger body size late in life and longer lifespans? Are these loci discrete sets?
4. How do the dynamics and action of loci and their many interactions support or refute predictions made by major evolutionary theories of ageing—the mutation accumulation theory^19^, the antagonistic pleiotropy theory^20^ and the longevity-assurance/disposable soma theory^21^?

To dissect age-localized genetic effects on mortality and lifespan we relied on the largest study of mouse ageing—the National Institute on Aging’s Interventions Testing Program (ITP)^22^. For more than two decades, teams at three sites have used a population of genetic siblings called the University of Michigan Heterogeneous Cohort 3 (UM-HET3) to study effects of drug interventions on longevity^22,23^. These mice are effectively one large sibship segregating for 11 million DNA variants. In earlier work we mapped lifespan using 3,055 mice and detected seven lifespan loci^17^. Here we have doubled the sample size to 6,438 mice, quadrupled numbers of genetic markers, and introduced a more powerful actuarial mapping method. The result has been a fourfold increase in numbers of lifespan loci (*Vita* type) and the confirmation of six of seven loci that we had previously mapped. In addition, we have mapped 30 loci of a new type that specifically influence correlations between body mass and life expectancies in males and females (*Soma* type loci) as a function of age. We also comprehensively analysed age-dependent heritability changes, and the strong sex- and age-dependent epistatic interactions among all loci.

The dynamics of *Vita* and *Soma* loci are highly complex. Some act almost exclusively at younger or older ages. A subset modulates life expectancies more uniformly and includes DNA variants that may act as pacemakers of ageing^24^. Many have marked and even complementary or antagonistic sex differences, as do almost all epistatic partnerships. Some loci have effects that are predicted by the theory of antagonistic pleiotropy^19,25,26^, whereas others have effects long after the reproductive phase of life and are consonant to different degrees with both the mutation accumulation theory and the longevity-assurance or disposable soma theory of ageing^2,27^. It should now be practical to convert some of these loci into well-defined molecular and cellular mechanisms that modulate age-dependent rates of ageing and risks of mortality with potential translational relevance to human health and longevity.

**Fig. 1:**
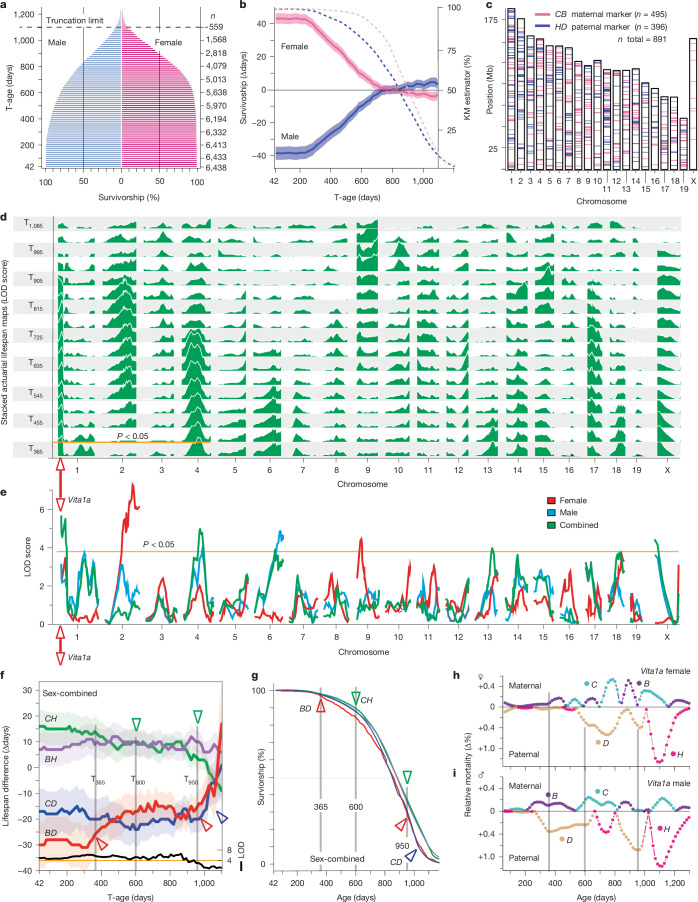
Survivorship sizes, sex differences and *Vita* lifespan loci. **a**, Survivorships of mice stratified by the minimum inclusion age (T-age, left *y* axis) starting with a full base population of 6,438 at the youngest T-age of 42 days (T_42_) and extending to a high truncation limit (dashed horizontal line) that only includes the subset of mice living to at least 1,100 days (T_1,100_). This final survivorship group that we studied contains 559 mice. The first death was at 46 days, and the last death was at 1,456 days. Numbers of survivors are indicated on the right *y* axis. **b**, Sex differences in lifespans of the T_42_ to T_1,100_ survivorships. Lines indicate mean and shaded bands represent s.e.m. The 81-day female lifespan advantage at T_42_ is neutralized by T_725_. Dashed lines show Kaplan–Meier (KM) estimators for both sexes. **c**, Ideogram of chromosomes and approximate positions of single nucleotide polymorphisms (SNPs) on the GRCm38/mm10 genome assembly that were used to define maternal (*C* versus *B*) and paternal (*H* versus *D*) haplotypes. **d**, Seventeen stacked genetic maps for the sex-combined (green filled) survivorships in 45-day steps (but computed in 15-day steps) from T_365_ to T_1,085_. The *y* axis of each stack gives the LOD with genome-wide significance at *P* < 0.05, correcting for numbers of markers tested and using a Cauchy correction for time-series tests. The vertical red line with arrowheads in the lowest T_365_ map highlights *Vita1a* and points to an expanded version in **e**. All survivorship maps are provided in Extended Data Fig. 1. **e**, A detailed version of a survivorship map, in this case of mice living to at least 365 days (T_365_) for females, males and sex-combined and sex-adjusted data. Here we use the same statistical thresholds as in **d**, but stratified by sex. Red arrowheads mark the *Vita1a* locus. **f**, Actuarial genetic effect size plots for genotypes at *Vita1a* (sex-combined) from T_42_ to T_1,100_. Coloured lines represent mean lifespan differences of survivorships of the four genotypes (*CH*,* BH*,* CD* and* BD*) as a function of T-age relative to the average of all mice and shaded bands represent s.e.m. estimates. LOD scores by T-age are plotted as a black line, with an orange line at the threshold of *P* < 0.05. The trio of shaded vertical lines at T_365,_ T_600_, and T_950_ in **f**–**h** are to help readers compare these three different plot styles of survival and mortality data. Red, green and blue triangles mark inflection points in mean lifespan of *BD*, *CH* and *CD* survivorship, respectively, at T_365_ and T_950_. **g**, Kaplan–Meier plot of survival stratified by genotypes with arrowheads as marked in **f**. **h**,**i**, Plots of age-dependent differences in relative mortality rates at *Vita1a* in females (**h**) and males (**i**). Each panel is divided into an upper maternal block with mortality rates of the *C* and *D* maternal haplotypes, and a lower paternal block with relative mortality rates of paternal *D* and *H* haplotypes. Deviations away from zero in either direction signify a relative increase in mortality for that haplotype relative to the alternative haplotype. Note that the *D* haplotype is strongly disadvantageous only before 900 days, whereas the *H* haplotype is disadvantageous after 1,000 days. We used a LOESS regression with an *α* of 0.2 over 75-day mean haplotype mortality counts. Grey vertical lines are provided for comparison across sexes in **f**,**g**.

**Fig. 2:**
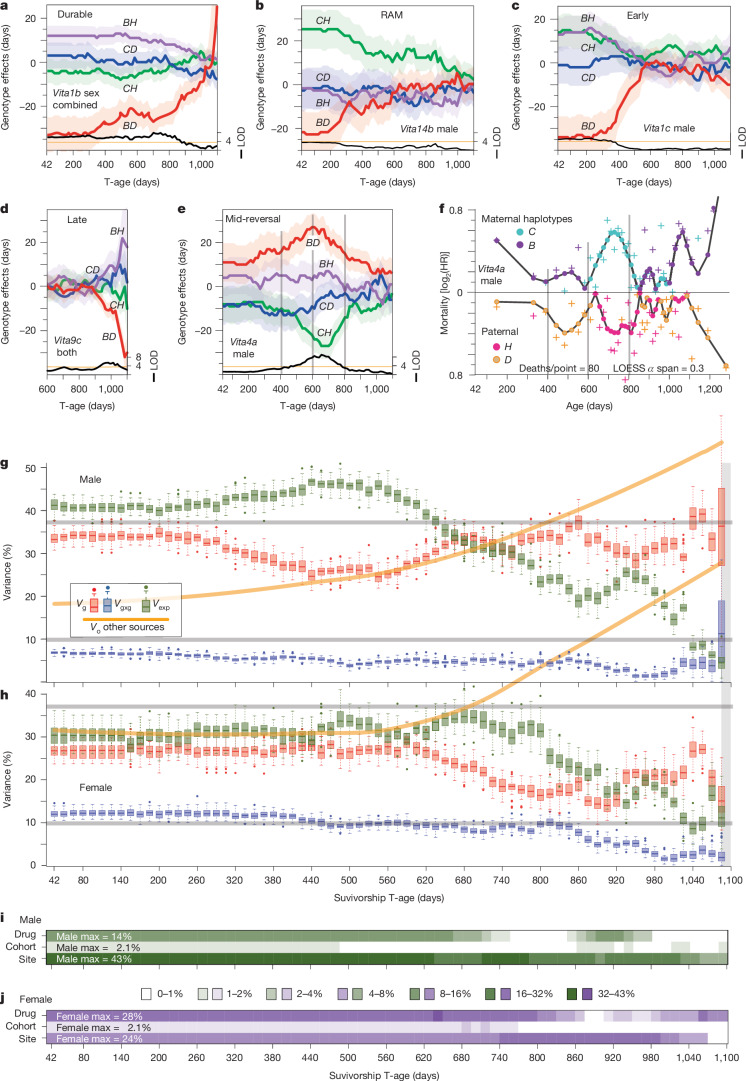
Dynamics of *Vita* loci, their contributions to heritability, and source of variance. **a**–**f**, Genetic effect plots define differences in the lifespan (mean ± s.e.m.) of survivorships for the four genotypes (*BH*, *BD*, *CH* and *CD*) for different categories of *Vita* loci. The effects of genotypes in days are marked by coloured lines (relative difference from the average). Two-letter labels define genotypes (pairs of haplotypes). LOD scores are plotted as a black trace. Extended Data Fig. 2 provides these plots for sex-combined data, females and males. **a**, *Vita1b* has durable and relatively uniform actuarial effects that extend from T_42_ to T_900_ survivorships. The initially negative effect of the *BD* genotype in most survivorships is caused by higher mortality visible starting in the T_855_ survivorship. **b**, *Vita14b* is a candidate rate of ageing modulator that has its highest LOD scores in the base male survivorship at T_42_, which reflects a lifelong and uniform contrast in mortality rates between *BD* and *CH* genotypes. All modes of display are compared for this locus in Extended Data Fig. 5, enabling readers to compare survivorship plots with age-dependent mortality. **c**, *Vita1c* has early effects in male mortality from inflections at T_365_ and T_590_. **d**, *Vita9c* is an example of a locus with late effects after T_935_ due to complex sex differences (Extended Data Fig. 2q). **e**, *Vita4a* has a marked reversal of genotype effects between T_450_ and T_810_—indicated by three vertical grey lines in **e**,**f**—that is caused by offset waves of mortality in males: an early phase of haplotype *C* mortality starting at 400 days (**f**) and a delayed phase of haplotype *H* mortality starting at 560–600 days that both peak at roughly 750 to 800 days. **f**, Age-dependent hazard ratio (HR) for males at *Vita4a*. Each crosshair point represents 80 mortality events over an age range centred on the point that have *C* or *B* maternal haplotypes (top) or *H* or *D* paternal haplotypes. Points were smoothed using a LOESS regression with a span of 0.3 (Methods). **g**,**h**, Comparisons of the variance explained across all survivorships specifically by peak markers. The pink box plots (*V*_g_) are estimates of variance that can be explained by all 29 *Vita* loci. The blue box plots are similar estimates of variance explained by the subset of significant *Vita*–*Vita* epistatic interactions (*V*_g__×__g_) defined in Q1 and Q2 of Fig. 5b. The green box plots (*V*_exp_) estimate the summed experimental variance that is further broken down in **i**,**j**. The heights of all variance box plots are computed on the basis of *n* = 50 independent subsampling replicates, each derived from an 80% random subset of the total dataset at each of 72 T-ages. In the final and smallest survivorship at T_1,100_, the sample size is 252 females and 248 males, somewhat less than the 559 well-genotyped mice at this T-age. The orange lines are smoothed averages of other sources of variance (*V*_o_), including mainly increased later-onset disease-associated mortality, gene-by-environmental effects and higher-order genetic factors such as indirect genetic effects of co-housed mice. **h**, Female components as above, but note the significantly lower variance values compared with males. The grey horizontal lines at 10% and 37.5% in **g**,**h** are to help readers compare variance levels between sexes. **i**,**j**, Three key experimental sources of variance by survivorship. Rows labelled ‘Drug’ are variance estimates of dietary supplements versus control diet. ‘Cohort’ is variance associated with year of production of mice—nine years in total. ‘Site’ is variance attributable to differences among the three vivaria used to house the mice. For a display of *V*_g_ of individual *Vita* loci for all survivorships, see Extended Data Fig. 6. See Supplementary Tables 2 and 3 for sample sizes by sex and T-age, and for the fit of statistical models.

**Fig. 3:**
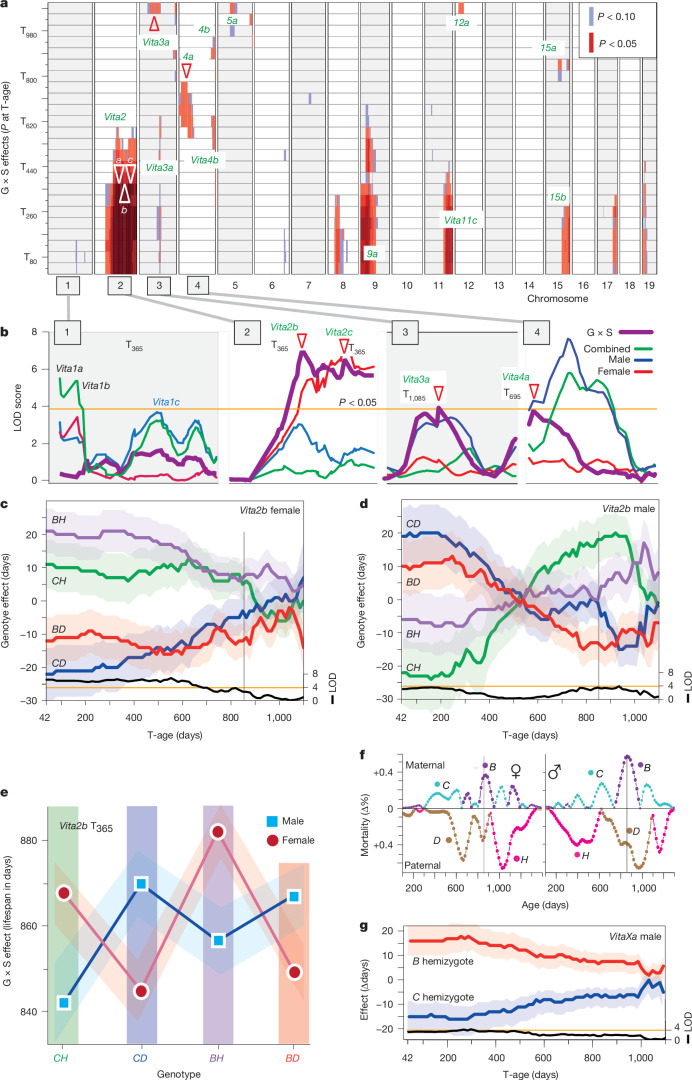
Antagonistic sex effects within *Vita* loci. **a**, Overview of all significant (red) and suggestive (light blue) locus-by-sex interaction effects (G × S) across all *Vita* loci in all survivorships. The G × S effect on chromosome 8 is most probably a real interaction of a locus that has a main effect below our threshold. The colour intensity indicates the LOD for the G × S term at genome-wide significance levels of *P* < 0.05 and *P* < 0.10, correcting for numbers of markers tested and using a Cauchy correction for time-series tests. The G × S values in Table 2 are more discrete and only test top markers in the most significant survivorship. **b**, Chromosomes 1 to 4 (magnified 3.7-fold on the *x* axis) illustrate the range of sex differences in effects of *Vita* loci at T_365_. Red and blue lines signify female and male additive genetic effects (LOD scores), whereas the thicker purple lines signify the G × S effect, and the green lines are the ‘unsexed’ or combined additive effect. The orange line is the genome-wide significance threshold at LOD of 3.95 and the Cauchy correction. There are no G × S effects on chromosome 1. *Vita* loci with significant G × S effects on chromosomes 2, 3 and 4 are labelled in green with the T-age of peak G × S at red triangles. **c**,**d**, Female (**c**) and male (**d**) *Vita2b* genotype effects are strongly antagonistic. The notable reversals in mean male life expectancies of genotypes in **d** are caused by higher mortality rates of carriers of the *C* and *H* haplotypes up to about 700 days, followed by higher mortality of *D* and *B* carriers from 800 to 1,100 days (vertical lines at peak mortality of around 850 days). Data are mean ± s.e.m. **e**, The G × S difference of lifespan of males and females for all genotypes. At T_365_ the *CH* and *BH* genotypes are advantageous for life expectancies of females, whereas the *CD* and *BD* genotypes are advantageous for life expectancies of males. Data are mean ± s.e.m. **f**, Mortality rate differences as a function of age, sex and parental haplotypes as in Fig. 1h. In females, mortality rates of *D* carriers peak at 650 days, whereas mortality rates for *H* carriers do not peak until 1,040 days. In males, the *B* and *D* haplotypes have a leptokurtic distribution that peaks between 700 and 1,050 days, whereas the *C* and *H* haplotypes have a more uniform mortality distribution across lifespan. **g**, The hemizygous *B* haplotype in males at *VitaXa* has a mean 30-day positive effect on lifespan compared to the *C* haplotype until T_300_. The standard error bands (shaded) are relatively stable even in the oldest male survivorships owing to the truncated age range of deaths.

**Fig. 4:**
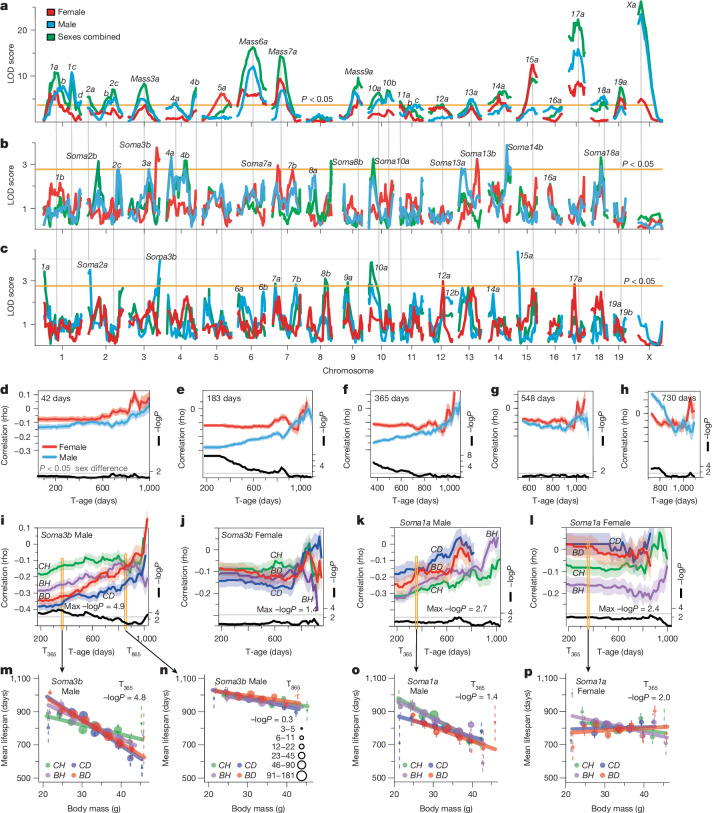
The genetic modulation of life expectancy by body mass is stronger in males than females. **a**, Conventional quantitative trait loci (QTL) maps of body mass (*Mass* loci) at 183 days for both sexes and combined. There are 25 significant *Mass* loci at T_183_ (see Extended Data Fig. 7 for all other ages). The yellow horizontal lines in **a**–**c** are genome-wide acceptance thresholds. The faint vertical grey lines at *Mass* peaks do not overlap significantly with *Soma* loci. **b**, Maps of the *Soma* loci that modulate correlations between body mass at 42 days and life expectancy in the T_42_ survivorship. Several loci are named despite being below threshold at this age or in **c**. **c**, Corresponding *Soma* loci for body mass at 183 days (6 months) with life expectancy at T_185_. **d**–**h**, Actuarial plots for correlations of body mass at 42, 183, 385, 558 and 730 days of age for males and female mice. Error bands in **d**–**l** represent 50% confidence intervals computed using a Fisher *z*-transformation to ensure that bounds remained within limits of –1 to +1. This visualization allows for a direct assessment of the estimate’s reliability; a narrower interval indicates a more precise estimate of the sample correlation coefficient. The –log*P* significance of the correlation difference was computed using a modified Fisher *r*-to-*z* transformation test (Methods). **d**, There is only a modest correlation of body mass at 42 days with subsequent lifespan in any survivorship in either sex. **e**, By 183 days, there is a strong negative correlation in males and a significant sex difference (–log*P* of the sex difference has a FDR of 0.05 at a value of 1.64). **g**, There is no sex difference at 548 days. **h**, Body mass at 730 days has a positive correlation in males, but by the T_1,040_ survivorship the female correlations are higher. **i**,**j**, Actuarial plots of correlations between body mass at 183 days with subsequent survivorships for *Soma3b* for males (**i**) and females (**j**). **i**, Correlations of the four colour-coded genotypes differ significantly for males in the T_365_ survivorship (left orange bar) but not in the older T_665_ survivorship. The –log*P* *Soma* scores are shown on the right axis, with the maximum actuarial value. Note arrows from orange bars to **m**,**n**, cross-sectional views of these body mass to life expectancy correlations at T_365_ that are highly significant in the T_365_ survivorship with a –log*P* of 4.8, but not significant in the T_865_ survivorship in **n**. **k**,**l**, Survivorship for males (**k**) and females (**l**) at *Soma1a*. *Soma1a* is close to the significance threshold in males in the T_710_ survivorship. Orange bars correspond to **o**,**p**. LOD scores are significant at 2.75. **m**,**n**, Correlation of lifespan and body mass at 183 days of age for *Soma3b* (Spearman rank rho) in males in T_365_ (**m**) and T_865_ (**n**) survivorships. Survivorships were binned in 2-g classes. The six circles sizes provide approximate sample size per class scaled to the log of the number. **o**,**p**, *Soma* correlations of lifespan and body mass at 183 days in males (**o**) and females (**p**), corresponding to **k**,**l** at T_365_.

**Fig. 5:**
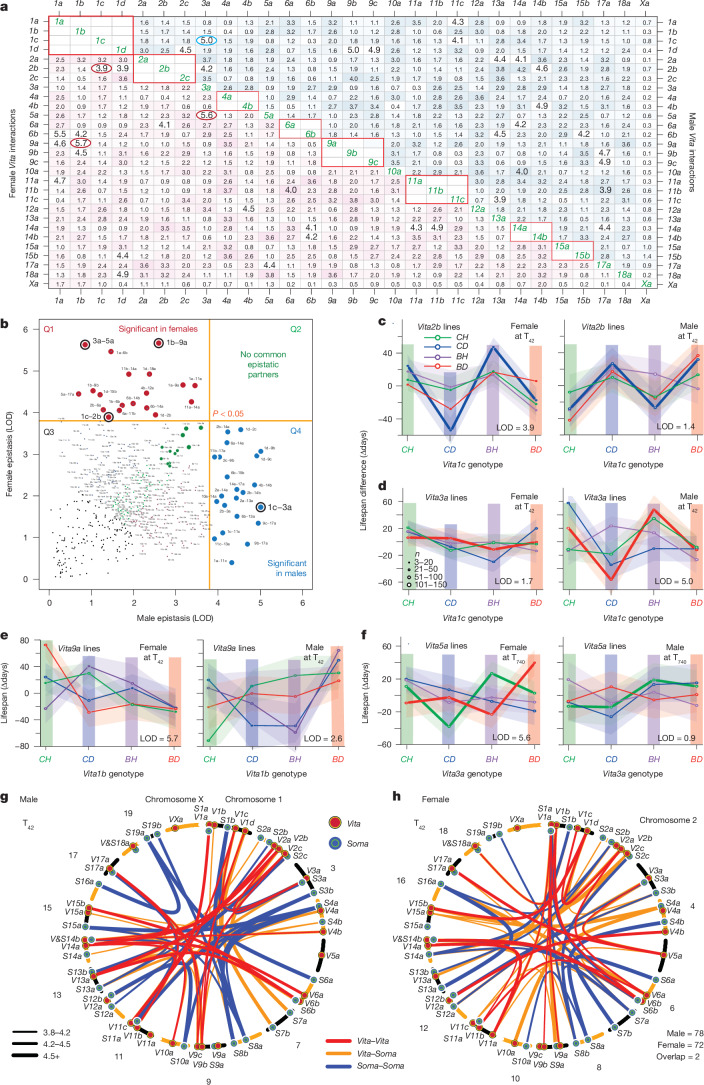
Epistasis among *Vita* and *Soma* loci. **a**, A full matrix of LOD scores of interactions among *Vita* loci in the T_42_ survivorship. The upper triangle tabulates male scores and the lower triangle tabulates female scores. LODs at or above 3.9 (*n* = 22 detected in males, *n* = 19 detected in females), labelled in larger font, are significant with a Bonferroni correction for 387 tests per sex that excludes 19 tests between syntenic loci (for example, between *Vita1a* and *Vita1b*). Similar matrices are shown for all four ages, for three sex combinations—combined, females and males—in Supplementary Table 10. **b**, Correlation plot of female versus male epistasis in the T_42_ survivorship. Those above the threshold are marked in red for females and in blue for males, and are labelled by their *Vita* pair numbers. Note that there are no points with significant LODs that are shared by both sexes (quadrant Q2). However, the green points in quadrant Q3 highlight some low-level interactions shared by both sexes. The Spearman rank order correlation of male and female LOD scores is 0.32. **c**, A pair of effect-size plots of a strong female epistatic interaction between *Vita1c* and *Vita2b* at T_42_ that is also highlighted with a bullseye in **b**. The coloured columns in **c** define the four genotypes at the first locus (*Vita1c*), whereas the four coloured lines define the genotypes at the second locus (*Vita2b*). Compare polarities of female and male effects of the *CD* genotype (thicker blue lines)—an example of modest antagonistic epistasis. **d**, Right, interaction between *Vita1c* and *Vita3a* in males, highlighted by the blue bullseye in **b**. Left, there is no interaction in females. **e**,**f**, Epistatic interactions at T_42_ (**e**) and T_740_ (**f**). The *Vita1b–Vita9a* interaction illustrates the classic masking effect, with all lines converging in the pink *BD Vita1b* column. The effects in males are not significant. The strong *Vita3a*–*Vita5a* interaction is weak in the T_42_ female survivorship (**e**) but strong at T_740_ (**f**). Point sizes indicate numbers of individuals per genotype pair. In** c**–**f**, the *y* axis shows estimates of the lifespan difference of the 16 small points in each panel and the shaded bands represent the s.e.m. **g**,**h**, Overview of epistatic interactions in the T_42_ survivorship with LOD scores above 3.8 (thin lines), above 4.2 (medium lines), and above 4.5 (thick lines), corresponding to Benjamini–Hochberg correction for 1,471 tests at *P* values of about 0.1, 0.05 and 0.01, respectively. Chromosomes are labelled with dots and abbreviated *Vita* and *Soma* symbols. These epistatic relations provide key constraints when evaluating sets of candidate genes. Epistatic signals highlight 3.3% of all 11,768 pairwise tests. None of the interactions define novel loci per se and should be regarded as genetic and functional glosses of *Vita* and *Soma* loci. For example, if *Atp6v1h* is the main *Vita1a* effector, then biochemical partners include *Pex5*, *Phb2*, *Foxm1* and *Etfrf1* in *Vita6a*; *Keap1* and *Sesn3* in *Vita9a*; and *Inpp5j*, *Gck*, *Ogdh*, *Igfbp3* and *Pex13* in *Vita11a*. These genes are parts of networks centred on lysosomal stress, autophagy and mitophagy (*Atp6v1h*, *Foxm1*, *Inpp5j* and *Phb2*), or on β-oxidation and metabolic stress (*Etfrf1*, *Gck*, *Igfbp3*, *Ogdh*, *Pex5*, *Pex13*, *Keap1* and *Sesn3*). Eight out of 11 genes are listed in the GenAge database (build 21 from August 2023). Colour and line styles define partnership types. There are 78 links in males and 72 links in females in total, but only two overlap in both sexes. Extended Data Fig. 9 provides the same plots for all survivorships.

**Fig. 6:**
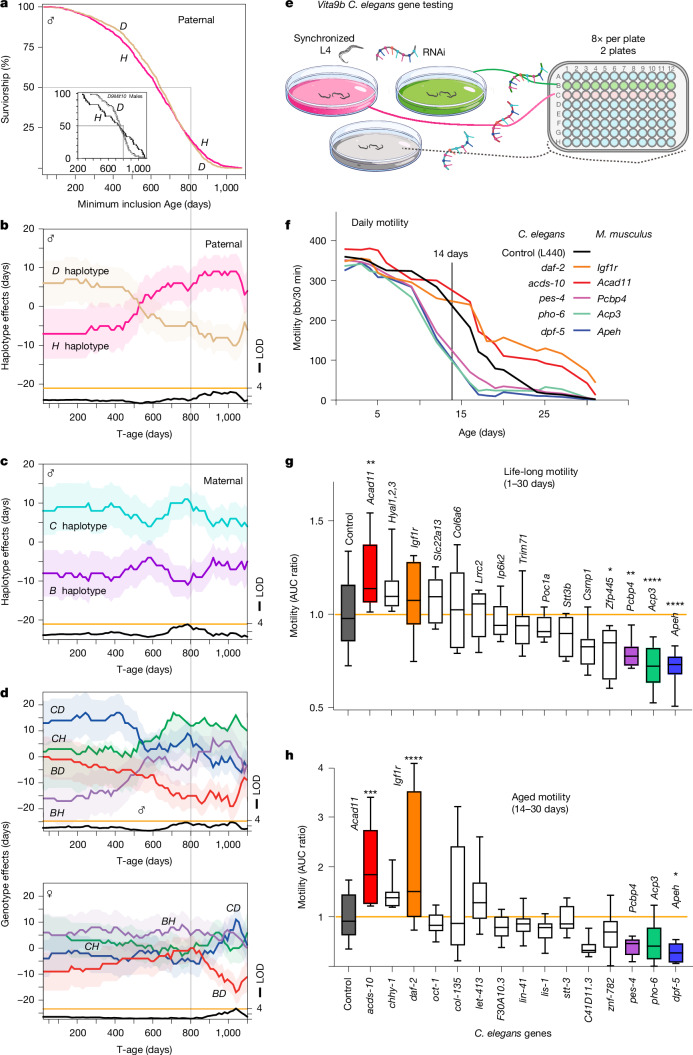
Genetics of *Vita9b* and candidate gene analysis. **a**, Kaplan–Meier plot for paternal haplotypes in male UM-HET3 mice at *Vita9b*, which corresponds to human chromosome 3p21.1 (46 to 53 Mb) and 3q22.2 (130 to 138 Mb). The *D* haplotype has a survival advantage compared with the *H* haplotype until 800 days of age (faint vertical line through **a**–**e**). The small Kaplan–Meier inset box is redrawn from Jackson et al. (2002), who detected the same effect at this locus in a separate cohort^53^ (linkage to marker *D9Mit10*). **b**,**c**, Contrasting effect plots with for the paternal (*H* and *D*) (**b**) and the maternal (*C* and *B*) (**c**) haplotypes, along with LOD scores at the bottom. Life expectancies of *H* and *D* haplotypes invert between T_250_ and T_900_. Those of *C* and *B* haplotypes are durable. Shaded bands show s.e.m. **d**, Corresponding genotype plots for males (top) and females (bottom) that are significant only in old survivorships. **e**, Design of the *C. elegans* motility assay, in which RNAi for candidate genes in *Vita9b* were used to knock down expression of target transcripts starting at the larval L4 stage; the first day of adult life. We used eightfold replication within plates and two plate replicates. **f**, Age-dependent change of motility in controls (black), *daf-2* (orthologue of mouse *Igf1r*) knockdowns (positive control, orange), and knockdowns of candidate genes with significant effects in **g**,**h**. The vertical black line at 14 days marks the threshold of senescence. **g**, Lifelong motility relative to controls (1 to 30 days) with knockdown of candidate genes and *daf-2* (*Igf1r*)-knockdown positive control. Box plots are based on at least 30 worms per well, 8 wells per clone and two independent screens. In **g**,**h**, we used two-tailed *t*-tests, assuming unequal variance to establish significance with a Bonferroni correction at *P < 0.1, **P < 0.05, ***P < 0.01 and ****P < 0.001. Ratios are normalized to control values integrated over the age range (ratios of areas under the curve (AUC)). Of the 15 genes tested in **g**, knockdown of three genes reduces motility significantly: *pes-4* (*Pcbp4*), *pho-6* (*Acp3*) and *dpf-5* (*Apeh*). By contrast, knockdown of *acds-10*, an orthologue of mouse *Acad11*, increases motility relative to the control, in a pattern resembling *daf-2* knockdown. **h**, Motility during the aged phase of life (14–30 days; reproduction typically ends before day 10). The positive effects of knocking down *daf-2* (*Igf1r*) or *acds-10* (*Acad11*) in this senescent stage are significant. Activity is reduced in *C41D11.3* (*Csrnp1*) and *pes-4* (*Pcbp4*) knockdowns with a nominal *P* value of 0.05, whereas *dpf-5* (*Apeh*) knockdown reduces motility with an adjusted *P* < 0.1.

The results are divided into six sections. The first section summarizes the actuarial mapping method and differences in survivorships by sex and genotype (Fig. 1 and Tables 1 and 2). The second partitions *Vita* loci by their dynamics and defines age-dependent shifts in heritable and non-heritable factors (Fig. 2). The third covers sex differences and antagonism (Fig. 3). The fourth introduces correlated trait mapping (Fig. 4) used to map 30 *Soma* loci (Tables 3 and 4) that modulate body mass–life expectancy trade-offs across age. The fifth is a systematic analysis of epistatic interactions among all loci (Fig. 5) and the discovery of a sharp split by sex. The last provides case studies of how genetic, bioinformatic and experimental data can be combined to move from maps towards genes and mechanisms of mortality and ageing (Fig. 6).

**Table 1:** Overview of *Vita* loci with effect sizes

				*Vita* positions (Mb, GRCm38)^c^							Survivorship T-ages^d^						Effect sizes^h^		
	Locus	Peak LOD^e^	Cauchy –log*P*^f^	Chr.	Proximal	Peak	Distal	Size (Mb)	Peak SNP ID	SNP Chr_Bp	From	Peak T-age	To	Duration	Max *n* at peak^g^	Mean lifetime (days)	Peak var male (%)	Peak var female (%)	Max peak diff. (days)
1	*Vita1a*	6.84	3.64	1	0.0	3.0	8.0	8.0	rs31443144	1_3010274	42	860	935	893	3,377	1,002	5.7	3.4	30
2	*Vita1b*	7.91	3.83	1	16.0	24.0	31.1	15.1	rs32645812	1_24042124	42	695	845	803	5,056	930	4.4	2.2	37
3	*Vita1c*	5.51	3.14	1	101.4	121.5	148.6	47.2	rs30776698	1_121483290	42	230	335	308	3,255	810	3.0	0.0	52
4	*Vita1d*	5.47	3.05	1	151.7	167.1	176.3	24.6	rs32557046	1_167148678	42	230	335	293	3,379	810	0.9	0.0	54
5	*Vita2a*	5.54	3.14	2	71.8	89.2	110.9	39.0	rs227254817	2_89156987	42	140	275	233	1,548	807	2.9	5.6	70
6^a^	*Vita2b*	6.84	3.51	2	110.9	112.7	130.9	20.0	rs27463377	2_112255823	575	800	890	315	4,079	970	0.5	1.1	20
7	*Vita2c*	8.13	4.61	2	132.8	148.4	158.4	25.6	rs29880912	2_148442635	42	545	665	623	2,930	906	0.0	1.2	30
8^a,b^	*Vita3a*	4.26	1.92	3	42.2	83.8	121.5	79.2	rs13477232	3_83354281	1,055	1,070	1,100	45	368	1,146	7.5	0.0	35
9^a^	*Vita4a*	9.00	4.84	4	38.9	52.5	63.3	24.3	rs13477705	4_52524395	42	650	710	668	2,588	908	8.2	1.6	53
10	*Vita4b*	4.43	2.38	4	132.4	154.3	156.0	23.6	rs250153474	4_154254581	500	635	680	180	2,652	900	2.5	0.0	33
11	*Vita5a*	4.05	2.10	5	36.6	67.6	90.2	53.6	rs3659098	5_67573068	965	1,085	1,100	135	350	1,149	8.0	0.0	36
12	*Vita6a*	4.14	2.93	6	97.3	107.4	122.3	25.0	rs38831890	6_93680853	42	140	560	518	3,396	807	2.2	0.0	38
13	*Vita6b*	5.27	3.05	6	108.1	132.8	149.7	41.7	rs31845771	6_132762500	42	320	515	473	3,296	823	2.6	0.0	47
14	*Vita9a*	5.68	3.75	9	13.4	29.9	44.7	31.2	rs38493199	9_34932404	42	95	725	683	2,754	910	0.9	5.0	35
15^a^	*Vita9b*	6.66	3.28	9	95.3	104.1	116.5	21.2	rs30513860	9_104091597	935	1,025	1,055	120	1,847	1,112	6.5	9.0	24
16	*Vita9c*	5.79	3.20	9	108.1	124.1	124.4	16.3	rs49693645	9_124056586	920	980	1,070	150	1,990	965	1.1	1.4	20
17^a^	*Vita10a*	4.48	2.48	10	56.7	72.8	100.5	43.7	rs13480647	10_72780332	965	980	1,010	45	1,812	1,078	5.8	0.2	23
18	*Vita11a*	4.21	2.20	11	0.0	5.6	30.1	30.1	rs26911247	11_6599922	560	635	695	135	2,652	901	3.4	0.0	35
19	*Vita11b*	4.07	1.96	11	59.0	82.2	109.4	50.4	rs234364394	11_82176894	995	1,040	1,055	60	586	1,114	0.0	9.1	29
20	*Vita11c*	4.91	2.91	11	97.9	113.7	119.3	21.3	rs29454463	11_113729074	42	42	290	248	3,332	806	1.7	0.2	44
21^a^	*Vita12a*	5.22	3.01	12	99.6	112.9	120.1	20.6	rs33465836	12_112855820	530	635	695	165	5,475	910	1.5	2.5	27
22	*Vita13a*	5.62	3.36	13	47.9	86.8	114.9	67.0	rs29246040	13_83858506	42	230	410	368	6,203	863	1.4	1.7	33
23	*Vita14a*	6.08	3.61	14	62.6	70.8	101.4	38.8	rs51512690	14_78415875	42	42	290	248	6,438	844	2.8	1.2	48
24^b^	*Vita14b*	4.86	2.87	14	78.4	101.4	120.3	41.9	rs49624430	14_101437466	42	80	290	248	3,377	1,006	2.1	0.4	18
25	*Vita15a*	5.08	2.89	15	55.5	74.2	88.4	32.9	rs45900875	15_74248242	785	905	950	165	3,549	1,023	5.7	1.9	21
26	*Vita15b*	5.73	3.14	15	99.3	102.2	104.0	4.7	rs31838876	15_99306167	42	95	275	233	3,398	813	1.3	0.0	40
27	*Vita17a*	4.97	3.07	17	0.0	32.9	73.7	73.7	rs6348789	17_32883804	440	665	740	300	5,286	925	3.8	1.3	23
28	*Vita18a*	4.56	2.73	18	33.2	52.5	81.0	47.8	rs45936103	18_52488251	42	140	455	413	6,431	858	1.5	1.6	28
29	*VitaXa*	5.64	3.28	X	0.0	36.0	69.8	69.8	rs13483724	X_36008085	42	260	485	443	3,332	806	6.6	0.0	35

^a^Marked loci in the left index column were detected in ref. ^17^ and replicated here. *Vita3a* has an early effect but linkage is significant only late in males.

^b^*Vita3a* and *Vita14b* marked in the index column are RAM-type loci with two T-ages with high LOD scores. *Vita3a* has an early effect but LODs are significant only late in males. The highest LOD for *Vita14b* is at T_860_ in combined data, but see Fig. 2b for early peak LOD in males.

^c^*Vita* chromosomes and positions using the GRCm38/mm10 assembly. Peak is the marker or imputed position with the highest LOD at peak T-age; proximal and distal refer to marker positions.

^d^Survivorship T-ages in 15-day truncation steps over which *Vita* loci are regarded as having an effect on lifespan centred around the peak T-age that gave the peak LOD for the first listed sex effects entry.

^e^Peak LOD values are significant at *P* values of 0.01, 0.05 and 0.10 with LODs of 4.65, 3.95 and 3.65, respectively.

^f^Cauchy –log*P* values are significant at ≥1.5.

^g^Max *n* at peak is the survivorship sample size at the *Vita* peak LOD at the peak T-age. For example, for *Vita1a* this number is 3,377 mice at T_860_, which had a mean survival of 1,002 days.

^h^Peak var male and peak var female are percentages of variance that a locus accounts for in the male or female survivorship, respectively with the peak LOD. See Extended Data Fig. 3 for variances explained at all T-ages in both sexes.

A full version of Table 1 is provided in the Supplementary Information.

**Table 2:** Overview of *Vita* loci with sex and epistatic effects and mortality features

		Mapping summary				Sex effects^a^			Epistatic sex effects		*Vita* mortality features	
	Locus	Peak LOD^b^	Chr.	Locus peak (Mb)	Peak T-age	Sex effects^a^	G × S at peak LOD^c^	Max G × S at any T-age^d^	Interactions in male^e^	Interactions in female^e^	Type and age of main effects^f^	
1	*Vita1a*	6.84	1	3.0	860	C, F, M	0.78	1.40	11c	6b, 9a, 11a	Durable, late reversal (M, F)	Extended Data Figs. 1 and 2a
2	*Vita1b*	7.91	1	24.0	695	C, F	0.78	0.78	-	6b, 9a, 9b	Durable, late reversal (M, F)	Extended Data Figs. 1 and 2b
3	*Vita1c*	5.51	1	121.5	230	M	2.41	2.62	3a, 11c	2b	Early (M)	Extended Data Figs. 1 and 2c
4	*Vita1d*	5.47	1	167.1	230	M	2.28	2.28	2c, 9b, 9c	2b, 15b, 18a	Early (M)	Extended Data Figs. 1 and 2d
5	*Vita2a*	5.54	2	89.2	140	M, F, X	9.78	9.78	13a, 14a	-	Early, reversal (M), RAM (F)	Extended Data Figs. 1 and 2e
6	*Vita2b*	6.84	2	112.7	800	F, C,X, M	0.35	8.62	3a, 14b	1c, 1d, 6a	Reversal (M), RAM (F)	Extended Data Figs. 1 and 2f
7	*Vita2c*	8.13	2	148.4	545	F, C, X	2.65	7.31	1d	-	Reversal (M), RAM (F)	Extended Data Figs. 1 and 2g
8	*Vita3a*	4.26	3	83.8	1,070	M, X	3.07	3.07	1c, 2b	5a	Early, RAM, reversal (M), durable (F)	Extended Data Figs. 1 and 2h
9	*Vita4a*	9.00	4	52.5	650	M, C, X	2.98	3.58	-	-	Mid, reversal (M), durable (F)	Extended Data Figs. 1 and 2i
10	*Vita4b*	4.43	4	154.3	635	M, X	3.30	3.64	14b	12a	Mid (M)	Extended Data Figs. 1 and 2j
11	*Vita5a*	4.05	5	67.6	1,085	M, X	2.85	2.91	-	3a, 17	Late (M)	Extended Data Figs. 1 and 2k
12	*Vita6a*	4.14	6	107.4	140	M, C	1.74	1.74	14a	2b, 11b	Early, mid (M)	Extended Data Figs. 1 and 2l
13	*Vita6b*	5.27	6	132.8	320	M, X	2.79	2.79	13a, 15a	1a, 1b, 14a, 14b	Early, mid, RAM (M)	Extended Data Figs. 1 and 2m
14	*Vita9a*	5.68	9	29.9	95	F, C, M, X	5.80	5.80	-	1a, 1b	Early (M), early, mid, RAM, reversal (F)	Extended Data Figs. 1 and 2n
15	*Vita9b*	6.66	9	104.1	1,025	C, F, M	1.21	2.11	1d, 17a	1b	Early, reversal, late (M), late (F)	Extended Data Figs. 1 and 2o
16	*Vita9c*	5.79	9	124.1	980	C, M, X	2.73	2.73	1d, 17a	-	Early, reversal, late (M), late (F)	Extended Data Figs. 1 and 2p
17	*Vita10a*	4.48	10	72.8	980	C, M	0.86	1.33	14a	-	Late (M)	Extended Data Figs. 1 and 2q
18	*Vita11a*	4.21	11	5.6	635	M	1.86	1.86	-	-	Mid reversal (M)	Extended Data Figs. 1 and 2r
19	*Vita11b*	4.07	11	82.2	1,040	F, X	1.86	3.45	-	1a, 14a	Durable, late reversal (F)	Extended Data Figs. 1 and 2s
20	*Vita11c*	4.91	11	113.7	42	M, X	4.41	4.71	1a, 1c, 13a	-	RAM (M)	Extended Data Figs. 1 and 2t
21	*Vita12a*	5.22	12	112.9	635	C	0.03	0.94	-	4b	Durable (M), durable (F)	Extended Data Figs. 1 and 2u
22	*Vita13a*	5.62	13	86.8	230	C, M	0.29	1.68	2a, 6b, 11c	-	RAM (M), mid (F)	Extended Data Figs. 1 and 2v
23	*Vita14a*	6.08	14	70.8	42	C, M	1.62	1.69	2a, 6a, 10a, 17a	6b, 11a, 11b	Early (M), RAM (F)	Extended Data Figs. 1 and 2w
24	*Vita14b*	4.86	14	101.4	80	C, M	0.22	1.80	2b, 4b	6b	RAM (M), RAM (F)	Extended Data Figs. 1 and 2x
25	*Vita15a*	5.08	15	74.2	905	C, M, X	1.36	3.25	-	-	Early, mid, RAM (M), durable (F, C)	Extended Data Figs. 1 and 2y
26	*Vita15b*	5.73	15	102.2	95	M, X	4.11	4.11	6b	1d	Early, mid, late (M)	Extended Data Figs. 1 and 2z
27	*Vita17a*	4.97	17	32.9	665	C, M	0.77	2.24	9b, 9c, 11b, 14a	5a	RAM (M)	Extended Data Figs. 1 and 2aa
28	*Vita18a*	4.56	18	52.5	140	C	1.49	1.63		1d	Early (M), RAM (F)	Extended Data Figs. 1 and 2ab
29	*VitaXa*	5.64	X	36.0	260	M	NA	NA	-	-	RAM (M)	Extended Data Figs. 1 and 2ac

NA, not applicable.

^a^Sex effects associated with the high linkage scores: combined (C), female (F), male (M) and G × S effect (X). The order is determined by LOD score, from high to low. Peak LOD only applies to the first sex effects entry.

^b^Peak LOD values are significant at *P* values of 0.01, 0.05 and 0.10 with LODs of 4.65, 3.95 and 3.65, respectively.

^c^G × S LOD. Values are significant with Bonferroni corrections at *P* ≤ 0.05 with LOD ≥ 2.75 when tested at the peak LOD marker and peak T-age.

^d^Maximum G × S LOD in any T-age survivorship

^e^Epistatic G × G effects by sex: *Vita* loci are listed by suffixes at LOD ≥ 3.8.

^f^Type and age of main effects depend on sex as noted. Durable, effects to ≥T_770_; RAM, gradual effect reduction to ≥T_800_; reversal, effect reversals; early, ≤T_500_; mid, T_500_ to T_845_; late, ≥T_860_.

A full version of Table 2 is provided in the Supplentary Information.

**Table 3:** Summary of *Soma* loci linkage statistics, age ranges and positions

									*Soma* positions (Mb, GRCm38)^a^					
	Locus	Max –log*P* any T-age^b^	High –log*P* at age weighed^c^	High T-age^d^	Sex effects^e^	Effect range (days g^−1^)	Actuarial range (days)^f^	Chr	Proximal	Peak	Distal	Size	Marker ID	SNP ID
1	*Soma1a*	3.95	3.42	185	C	8.3	≤365	1	0.0	3.0	24.0	24.0	1_3010272	rs31443144
2	*Soma1b*	4.09	3.85	730	M	4.3	≥730	1	63.9	86.2	133.6	69.7	1_86216552	rs240615003
3	*Soma2a*	3.56	3.50	185	M, C	10.4	≥183	2	0.0	13.6	24.6	24.6	2_13600088	rs29825025
4	*Soma2b*	5.07	3.12	42	C	47.4	≤548	2	30.4	60.2	83.9	53.5	2_60201233	rs29953805
5	*Soma2c*	5.09	2.77	42	M	30.8	42	2	148.4	161.9	180.1	31.6	2_161871392	rs27303276
6	*Soma3a*	3.53	2.76	550	M	5.5	42, ≥548	3	42.2	88.0	108.2	66.0	3_87974845	rs36895924
7	*Soma3b*	4.93	3.95	185	M	9.5	All	3	121.5	159.6	159.6	38.1	3_159581164	rs30858154
8	*Soma4a*	4.02	3.32	42	M	20.0	All	4	0.0	11.2	30.8	30.8	4_30761996	rs27779705
9	*Soma4b*	3.55	3.15	42	C, M, F	20.4	≤183, ≥730	4	66.8	107.4	156.0	89.2	4_107374161	rs28147090
10	*Soma6a*	3.99	3.60	550	C, F	3.1	All	5	0.0	8.0	25.9	25.9	6_8006720	rs49698565
11	*Soma6b*	3.46	3.13	730	F	4.0	≥730	6	66.8	138.7	139.6	72.8	6_138658041	rs30021501
12	*Soma7a*	5.27	4.75	365	M	7.6	≥183	7	4.3	16.1	43.5	39.2	7_16072018	rs32395309
13	*Soma7b*	3.84	3.79	550	M	7.5	≥183	7	85.1	120.1	145.3	60.2	7_120086292	rs31151709
14	*Soma8a*	3.07	2.92	365	M	6.9	≤365	8	41.1	71.7	86.0	44.9	8_71684276	rs33469281
15	*Soma8b*	3.48	3.13	42	C	22.9	All	8	86.0	111.3	129.1	43.1	8_126505019	rs33182145
16	*Soma9a*	2.93	2.92	185	C	6.6	183	9	20.0	51.1	58.1	38.1	9_51116640	rs13461391
17	*Soma10a*	4.49	3.85	185	C,M	7.1	≤183, ≥730	10	0.0	18.1	90.1	90.1	10_18144599	rs29381732
18	*Soma11a*	4.22	3.31	365	F	3.6	All	11	50.4	97.4	113.7	63.3	11_97448477	rs45809946
19	*Soma12a*	3.88	2.97	185	F	6.1	183	12	58.3	71.7	79.1	20.8	12_71677220	rs29155467
20	*Soma12b*	2.91	2.91	730	M	4.2	≥183	12	79.1	118.2	118.2	39.1	12_118179607	rs30534543
21	*Soma13a*	3.52	2.80	42	M	46.0	≤183	13	4.4	19.4	67.3	62.9	13_19367506	rs50623363
22	*Soma13b*	4.71	3.22	42	F	25.0	42	13	77.3	98.5	111.4	34.1	13_98521647	rs30068968
23	*Soma14a*	3.12	3.12	550	M	7.1	183–548	14	20.7	31.0	31.0	10.3	14_30957748	rs30464161
24	*Soma14b*	3.89	3.88	42	M	49.7	42	14	79.4	101.4	120.3	40.9	14_101437466	rs49624430
25	*Soma15a*	4.50	4.34	185	M	16.4	183–548	15	0.0	3.3	20.7	20.7	15_3288506	rs31623892
26	*Soma16a*	3.86	2.76	550	C, F	4.3	≥548	16	38.0	75.8	96.4	58.4	16_75758401	rs46542250
27	*Soma17a*	2.97	2.87	730	F, C	2.6	183 F, 548 M, 730 F	17	0.0	26.5	68.8	68.8	17_26542857	rs33475140
28	*Soma18a*	4.68	3.30	42	C	24.0	42 M, 42–365 F	18	21.1	52.5	70.8	49.8	18_52488251	rs45936103
29	*Soma19a*	3.32	3.32	730	M	4.4	730	19	0.0	3.4	11.6	11.6	19_3403302	rs31128510
30	*Soma19b*	3.00	3.00	730	M	5.1	730	19	35.0	53.9	62.0	27.0	19_53851357	rs30416321

^a^*Soma* chromosomes and positions using GRCm38 coordinates. Proximal and distal refer to marker positions.

^b^Max –lop*P* any T-age is the maximum value across any actuarial scan from any of the five ages at which mice were weighed. This value was not used to establish significance.

^c^High –log*P* at age weighed and max –log*P* any T-age are the highest scores corresponding to: (1) one of the five ages at which mice were weighed; or (2) the highest –log*P* at any T-age. Values are genome-wide significant at *P* < 0.05 with Benjamini–Hochberg correction at values ≥ 2.75.

^d^High T-age is the T-age at which mice were weighed that gave the high –log*P* at age weighed. If the high T-age is 730, then all values correspond to the T_730_ survivorship for the first listed sex effects.

^e^Sex effects listed in the order corresponding to data for high –log*P* and hIgh T-age. Combined (C); female (F) and male (M).

^f^Actuarial range in days associated with *Soma* loci. When two age ranges are shown, they apply in order of sex effects.

A full version of Table 3 is provided in the Supplementary Information.

**Table 4:** Summary of *Soma* loci effects and overlap with *Mass* and *Vita* loci

		*Soma* overview						Effect sizes								*Mass* overlap		*Vita* overlap	
								Days gained or lost per gat high T-age^a^				Correlation of body mass with lifespan (rho)^a^							
	**Locus**	**Max –log***P*^**b**^	**Sex effects**^**c**^	**Effect range (days g**^**−1**^)	**Chr.**	**Peak**	**Actuarial range (days)**^**d**^	***CH***	***BH***	***CD***	***BD***	***CH***	***BH***	***CD***	***BD***	***Mass*** **locus overlap**^**e**^	***Mass*** **locus type and sex**^**e**^	***Vita*** **locus overlap**^**e**^	***Vita*** **locus type and sex**^**e**^
1	Soma1a	3.95	C	8.3	1	3.0	≤365	−10.2	−11.8	−3.5	−4.5	−0.23	−0.26	−0.09	−0.12	-		*Vita1a*, *Vita1b*	Durable (C)
2	*Soma1b*	4.09	M	4.3	1	86.2	≥730	5.1	1.4	0.9	5.2	0.32	0.07	0.08	0.32	*Mass1b*	Early (M)	*Vita1c*	Early (M)
3	*Soma2a*	3.56	M, C	10.4	2	13.6	≥183	−11.4	−21.8	−14.4	−16.1	−0.22	−0.43	−0.29	−0.38	*Mass2a*	Early (C)		
4	*Soma2b*	5.07	C	47.4	2	60.2	≤548	7.3	2.7	−40.1	−30.2	0.07	0.03	−0.23	−0.23	-			
5	*Soma2c*	5.09	M	30.8	2	161.9	42	−13.9	−13.1	−5.8	−36.6	−0.09	−0.09	−0.05	−0.34	-		*Vita2c*	Mid (F, M)
6	*Soma3a*	3.53	M	5.5	3	88.0	42, ≥548	1.3	−1.0	−3.7	−4.2	0.03	−0.05	−0.12	−0.17	*Mass3a*	Durable (C)	*Vita3a*	Early, late (M)
7	*Soma3b*	4.93	M	9.5	3	159.6	All	−8.4	−13.7	−17.9	−16.7	−0.18	−0.29	−0.38	−0.34	-			
8	*Soma4a*	4.02	M	20.0	4	11.2	All	−15.5	−31.9	−14.1	−34.1	−0.13	−0.28	−0.11	−0.22	*Mass4a*	Early (M)		
9	*Soma4b*	3.55	C, M, F	20.4	4	107.4	≤183, ≥730	−12.9	−25.1	−4.7	−18.6	−0.13	−0.17	−0.02	−0.18	*Mass4b*	Durable (M)	*Vita4b*	Mid (M)
10	*Soma6a*	3.99	C, F	3.1	5	8.0	All	−2.9	−2.4	0.2	0.1	−0.15	−0.13	0.00	0.01	-			
11	*Soma6b*	3.46	F	4.0	6	138.7	≥730	−2.2	0.3	0.4	1.8	−0.17	0.02	0.04	0.12	-		*Vita6b*	Early (M)
12	*Soma7a*	5.27	M	7.6	7	16.1	≥183	−14.5	−6.9	−10.2	−7.0	−0.38	−0.20	−0.26	−0.15	-			
13	*Soma7b*	3.84	M	7.5	7	120.1	≥183	−2.0	−4.9	2.6	−4.8	−0.09	−0.17	0.06	−0.17	-			
14	*Soma8a*	3.07	M	6.9	8	71.7	≤365	−8.3	−5.3	−12.2	−10.4	−0.22	−0.12	−0.33	−0.27	-			
15	*Soma8b*	3.48	C	22.9	8	111.3	All	−11.9	−13.9	−28.5	−5.6	−0.11	−0.11	−0.25	−0.03	-			
16	*Soma9a*	2.93	C	6.6	9	51.1	183	−13.0	−6.4	−10.0	−9.0	−0.30	−0.17	−0.23	−0.22	-		*Vita9a*	Early (F)
17	*Soma10a*	4.49	C, M	7.1	10	18.1	≤183, ≥730	−9.8	−9.0	−3.8	−10.9	−0.25	−0.22	−0.09	−0.25	*Mass10a*	Early (M)	*Vita10a*	Late (M)
18	*Soma11a*	4.22	F	3.6	11	97.4	All	−0.1	−3.7	−2.3	−2.0	0.03	−0.18	−0.15	−0.10	*Mass11c*	Durable (M)	*Vita11b*	Late (F)
19	*Soma12a*	3.88	F	6.1	12	71.7	183	−1.2	−7.3	−4.5	−2.8	−0.05	−0.24	−0.16	−0.08	*Mass12a*	Durable (F)		
20	*Soma12b*	2.91	M	4.2	12	118.2	≥183	4.8	3.6	0.6	2.1	0.26	0.28	0.03	0.13	-		*Vita12a*	Mid (C)
21	*Soma13a*	3.52	M	46.0	13	19.4	≤183	12.3	−33.7	−25.1	−21.2	0.04	−0.28	−0.17	−0.21	-			
22	*Soma13b*	4.71	F	25.0	13	98.5	42	−1.5	−22.3	2.7	−19.3	−0.06	−0.25	0.01	−0.11	-		*Vita13a*	Early (F, M)
23	*Soma14a*	3.12	M	7.1	14	31.0	183–548	−0.1	−4.7	−0.8	2.4	−0.02	−0.17	−0.06	0.07	-			
24	*Soma14b*	3.89	M	49.7	14	101.4	42	−45.8	−6.9	3.9	−18.1	−0.35	−0.07	0.06	−0.12	*Mass14a*	Early (C)	*Vita14a*	Early mid (C)
25	*Soma15a*	4.50	M	16.4	15	3.3	183–548	−7.1	−3.6	−20.0	−13.5	−0.15	−0.09	−0.43	−0.31	-			
26	*Soma16a*	3.86	C, F	4.3	16	75.8	≥548	−5.1	−4.8	−9.1	−8.4	−0.15	−0.13	−0.26	−0.22	*Mass16a*	Early mid (M)		
27	*Soma17a*	2.97	F, C	2.6	17	26.5	183 F, 548 M, 730 F	−0.8	−1.1	1.5	1.1	−0.06	−0.09	0.14	0.09	*Mass17a*	Time diff (M, F)	*Vita17a*	Durable (M)
28	*Soma18a*	4.68	C	24.0	18	52.5	42 M, 42–365 F	−0.9	−13.2	−24.9	−14.5	0.02	−0.10	−0.24	−0.16	-		*Vita18a*	
29	*Soma19a*	3.32	M	4.4	19	3.4	730	4.1	−0.3	3.9	3.2	0.27	−0.02	0.21	0.21	-			
30	*Soma19b*	3.00	M	5.1	19	53.9	730	4.3	−0.8	3.0	2.8	0.23	−0.04	0.22	0.18	*Mass19a*	Early Mid (M)		

^a^Effect sizes for genotypes at the high T-age and first sex effects group. Days gained or lost effect sizes for *CH*, *BH*, *CD* and *BD* genotypes at the high T-age for first sex effects group. Similar values for correlations by genotypes of body mass with lifespan.

^b^Max –log*P* any T-age is the maximum value across any actuarial scan from any of the five ages at which mice were weighed. This value was not used to establish significance.

^c^Sex effects shown in the order corresponding to data for high –log*P* and high T-age: C, combined, F, female and M, male.

^**d**^Actuarial range associated with *Soma* loci. When two age ranges are given they apply in order of sex effects.

^e^High T-age is the T-age at which mice were weighed that gave the high –log*P* when actually weighed. If the high T-age is 730, then all values correspond to the T_730_ survivorship for the first listed sex effects. *Mass* locus overlap and *Vita* locus overlap with the *Soma* locus. *Vita* type and sex from Table 1.

The ‘Time diff’ label for *Soma17a* highlights early and late T-age effects in females, intermediate in males.

A full version of Table 4 is provided in the Supplementary Information.

## 29 *Vita* loci are defined by actuarial mapping

We analysed lifespans of 72 nested survivorships (Fig. 1a), each of which we generated by truncating from younger to older cut-offs in 15-day steps. The base survivorship includes mice that entered the study and survived to at least the first truncation age (T-age) of 42 days (Fig. 1a, T_42_). The terminal survivorship includes only the oldest 559 mice (8.7%)—those that reached at least 1,100 days of age (T_1,100_). At the T_42_ T-age the sex difference of lifespan is 81 days (Fig. 1b): 806 ± 210 days (mean ± s.d.) for males and 887 ± 175 days for females. This difference is stable to T_215_ because so few animals of either sex die before this age; only 20 males and 13 females. However, between 215 and 410 days 204 males die but only 18 females die, and by T_740_, expectancies of males and females have converged at 946 days (Fig. 1b).

**Extended Data Fig. 1:**
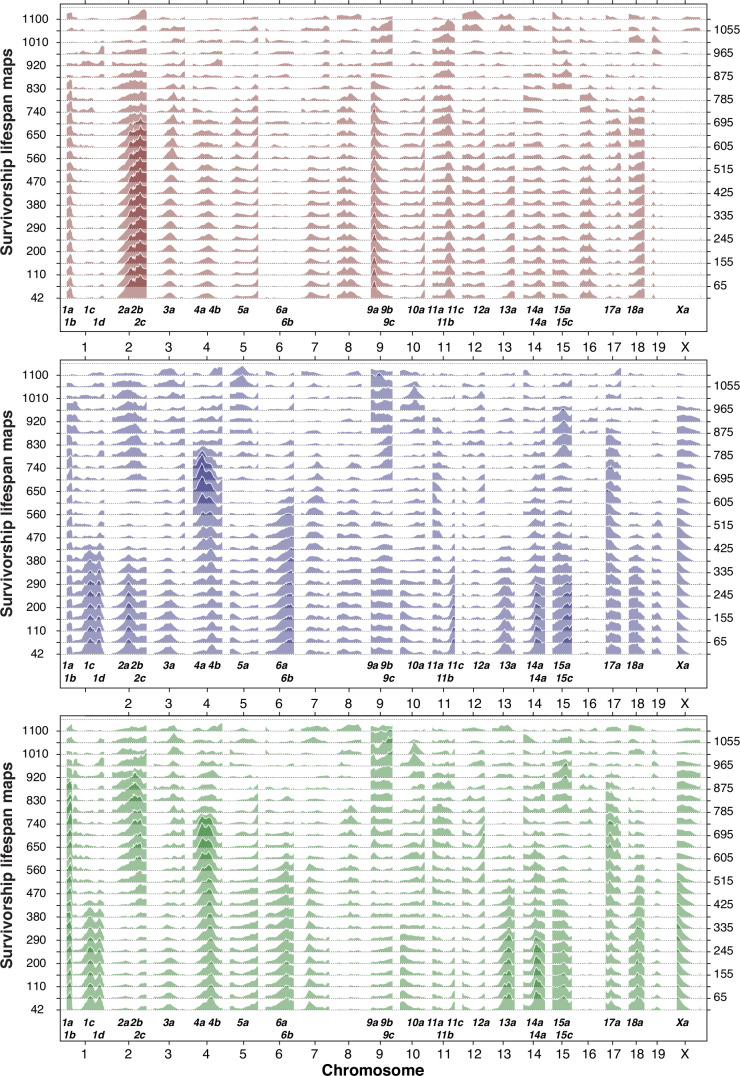
Sex-stratified and combined maps of mean lifespans of survivorships. **a,b,c**, This extends the content of Fig. 1d to include female and male survivorship maps across the entire range of survivorships. Other conventions as in Fig. 2d. The LOD threshold is defined by the horizontal dashed lines for each survivorship. Note that only every third survivorship is plotted, accounting for some *Vita* loci that do not reach significance here.

**Extended Data Fig. 2:**
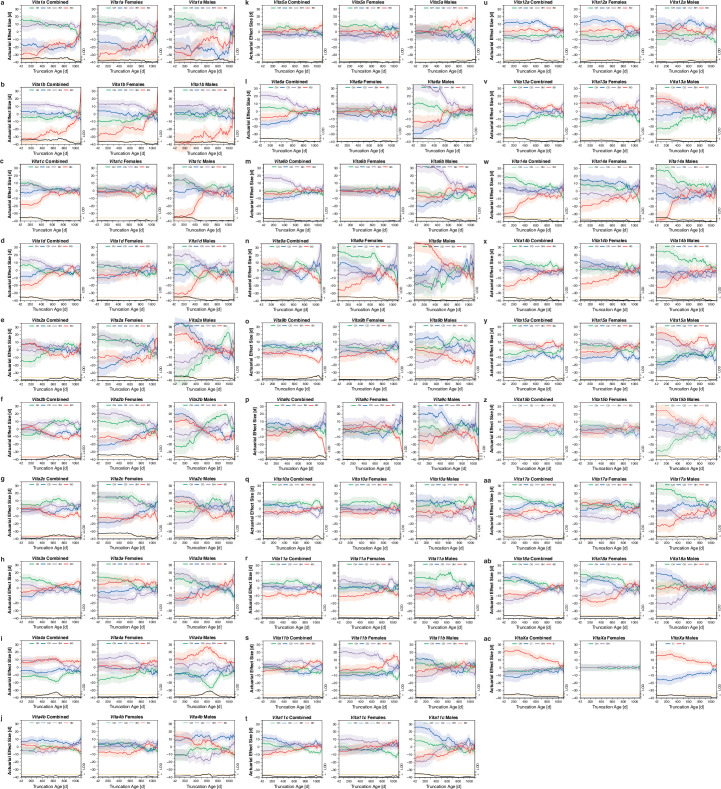
*Vita* loci genotype effect plots. Trios of effect size plots with standard error bands as described in detail in the legend to Figs. 1 and 2. Each trio for the 29 *Vita* loci includes combined data for both sexes (left), for females only (middle), and for males only (right). To understand the genetic sources that account for timing changes in mortality rates it is helpful to compare these more complex genotype plots to pairs of haplotype plots in Extended Data Figs. 3, 4.

**Extended Data Fig. 3:**
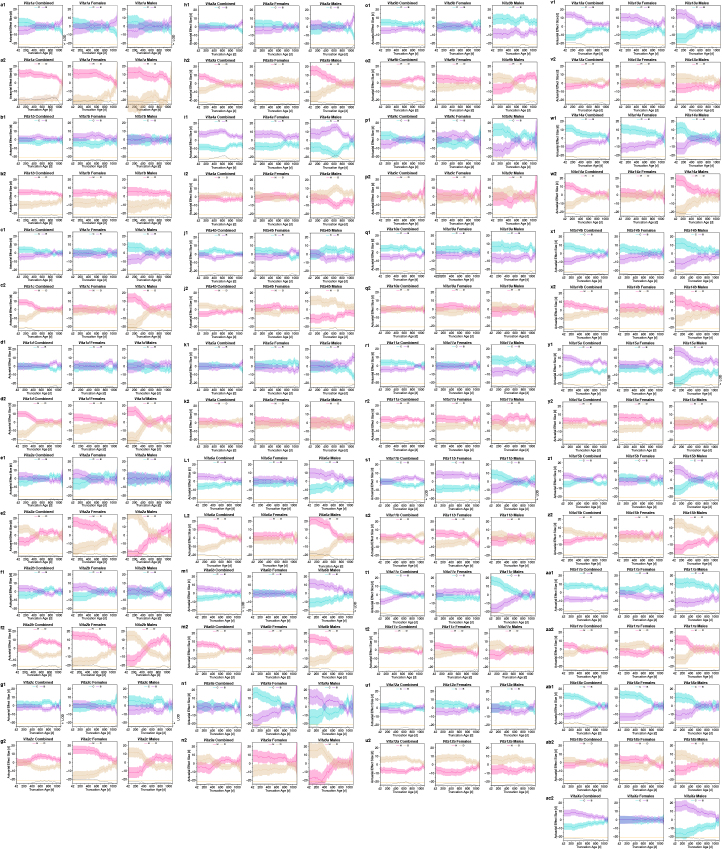
*Vita* loci haplotype effect plots. There are two trios of effect size plots for each *Vita* locus with standard error bands. The upper trio (blue and purple shades) give the difference in actuarial effects of the maternal haplotypes—*C* and *B*. The lower trio (pink and beige shades) give the difference in effects of the paternal haplotypes—*H* and *D*. These plots are symmetric and easier to interpret than those in Extended Data Fig. 2. However, they generally have lower LOD scores.

We mapped loci that modulate mean lifespans of survivorship at a false discovery rate (FDR) of *P* < 0.05 after applying a Bonferroni correction with an additional Cauchy correction for the actuarial analysis (Fig. 1c and Table 1). Mapping was stratified by sexes and in combination (Fig. 1d,e, Table 2, Extended Data Fig. 1 and Supplementary Tables 2–9 for trait and mapping data files). Loci have average effects of 36 ± 12 days (mean ± s.e.m.) on life expectancies that often depend strongly on sex and T-age (Fig. 1c–f, Table 1 and Extended Data Figs. 1–3). Peak effects explain 2.5 ± 0.9% of variance in females and 3.2 ± 0.5% in males. Linkage scores range from a low of 4.1 logarithm of the odds (LOD) (*Vita5a*) to a high of 9.0 LOD in males (*Vita4a*) and to a high of 8.1 LOD in females (*Vita2c*). Confidence intervals are 35 ± 19 Mb (mean ± s.d.) with the smallest two loci under 10 Mb (Fig. 1d-f and Table 1). All genes in loci were reviewed for variants that potentially affect function (Supplementary Table 1a,b). *Vita* loci have age-delimited effects that average 349 ± 229 days (mean ± s.d.) (Table 1, average of the ‘Duration’ column).

## Dynamics of *Vita* loci

**Extended Data Fig. 4:**
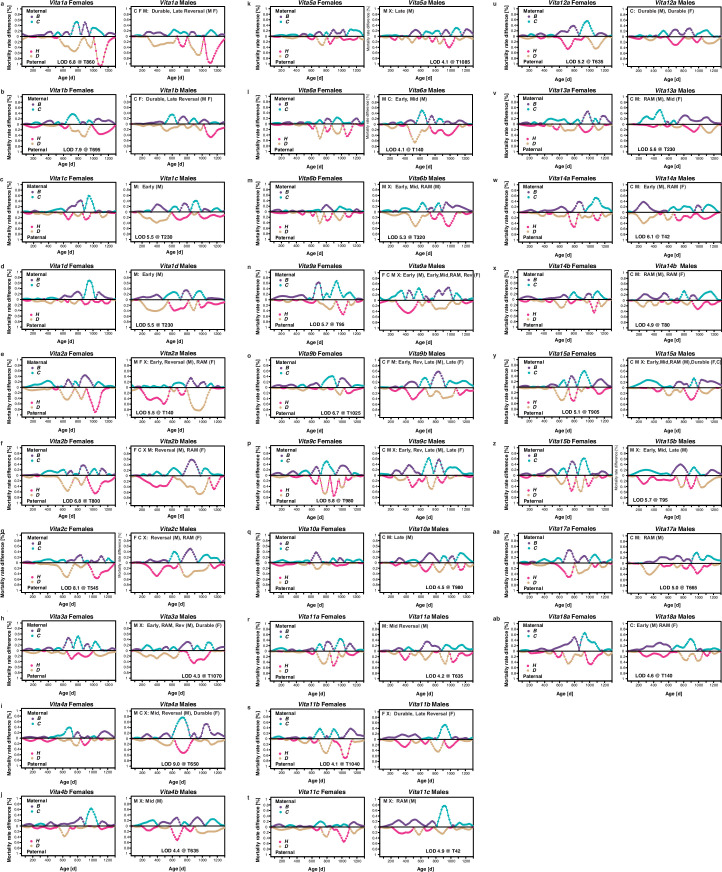
Mortality rates of maternal and paternal haplotypes of *Vita* loci. Relative mortality rate difference between the maternal pair of haplotypes (*C* and *B* on the top of each panel) and between the paternal pair of haplotypes (*H* and *D* on the bottom of each panel). Here we have used a LOESS smoother with a relatively short α span 0.2. At any one age only the haplotype with the higher rate of mortality is shown, accounting for abrupt reversals.

**Extended Data Fig. 5:**
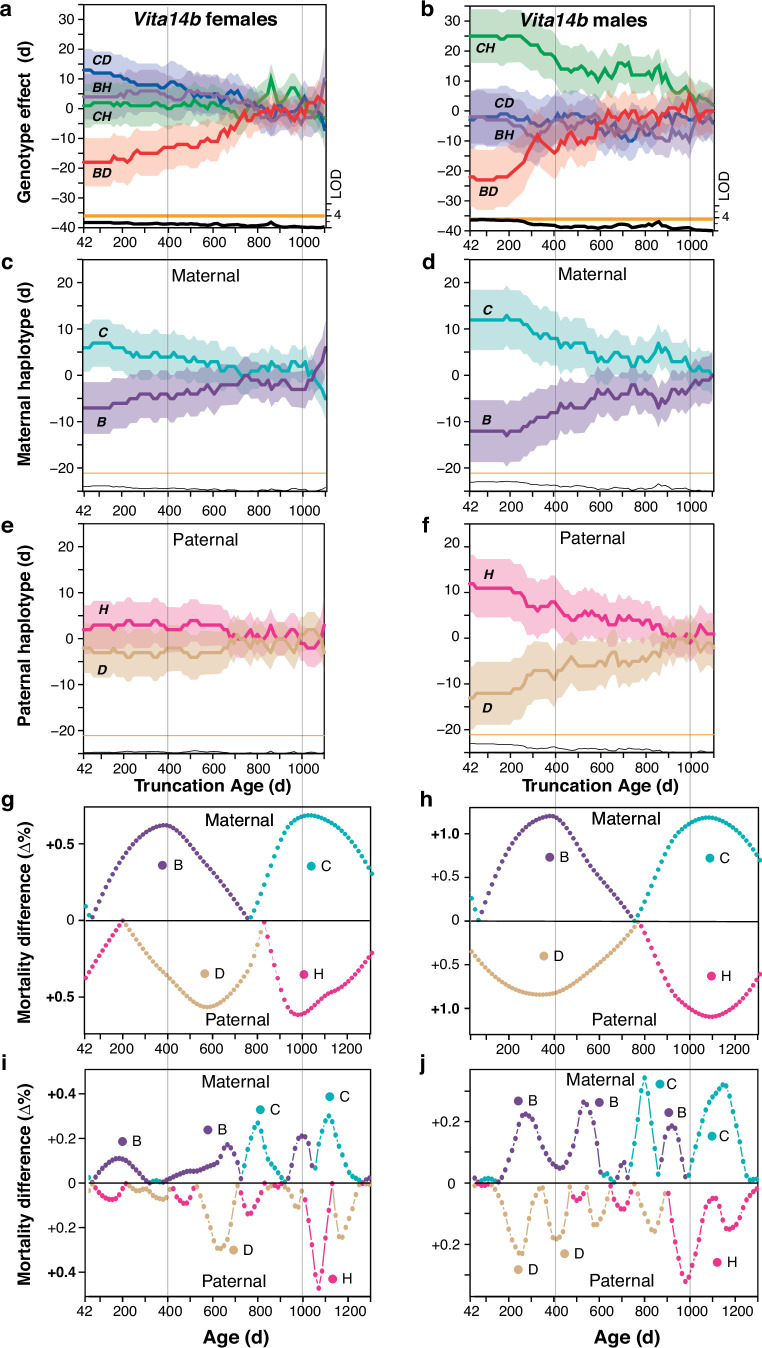
Direct comparisons of all methods of display of survivorship effect sizes of genotypes, haplotypes, and mortality rates of a *Vita14b* in females (left) and males (right). **a,b**, The actuarial genotype effect plots with standard error bands of *Vita14b* (see Fig. 2b) for both sexes. **c,d**, Corresponding haplotype-specific plots. **g,h**, Age-dependent relative mortality rate differences using a LOESS smoother with a span over the entire range of ages (*α* of 1) that averages mortality difference at a high level. While effects are similar between sexes, only the much stronger male effect in **b** reaches significance—the sum of the two reinforcing haplotype effects. **i**,**j**. An analysis of age-dependent differences in mortality rates using a LOESS with an α span of 0.2 that exposes much finer details of both age-dependent and haplotype-dependent differences in mortality. Extended Data Fig. 4 uses only this finer-grained smoother.

Effects of *Vita* loci are age-dependent (Fig. 1, Tables 1 and 2, Extended Data Figs. 1–4 and Supplementary Tables 2 and 3). *Vita1a* and *Vita1b* have stable actuarial effects from T_42_ to T_890_ (Figs. 1f and 2a) but strong late-age mortality effects. By contrast, *Vita14b* has a gradual reduction in effects (Fig. 2b) highlighted by the converging slopes of *BD* and *CH* genotypes from T_230_ to T_1,100__._ Other loci have transient effects. For example, *Vita1c* acts only in survivorships that include adults younger than 500 days (Fig. 2c), whereas *Vita9c* acts only in the oldest survivorships (Fig. 2d). *Vita4a* has marked effects that reverse between T_410_ and T_800_ in males (Fig. 2e,f). Inflection points in actuarial plots correspond to ages on Kaplan–Meier plots (Fig. 1g) at which mortality rates of genotype classes diverge or converge (Extended Data Figs. 2–5). In Figs. 1h and 2f there are, for example, strong age-dependent differences in mortality rates between maternal and paternal haplotypes by sex and age. The *D* haplotype contributes to higher mortality earlier in life; the *H* haplotype to higher mortality later in life. This explains the actuarial benefit even in the earliest survivorships of inheriting an *H* haplotype (Fig. 1f and Extended Data Fig. 4 for all other *Vita* loci). The impact of DNA variants within *Vita* loci on mortality is greatest at inflections in actuarial plots—at the peaks and troughs of mortality rate plots (Fig. 2a–f and Extended Data Figs. 2–4). These are the T-age ranges during which the mean lifespan of survivorships can swing by up to 15 days over just 45 days (Fig. 2e). By contrast, *Vita* loci that have nearly constant slopes from T_42_ to T_750_ (Fig. 2b) behave as expected of putative modulators of rates of ageing and of differences in all-cause mortality^5,24^.

We have categorized the dynamics of *Vita* loci into four categories (Table 2, far right) using the plots reproduced in Extended Data Figs. 2–4.

1. *Loci with durable effects*. Five loci have relatively constant effects on life expectancies of survivorships from as early as T_42_ out to the T_750_ survivorship in one or both sexes (Fig. 1f and Tables 1 and 2). Their actuarial durability makes them predictors of life expectancy even in adolescence, but this does not mean that they have age-independent effects on mortality. In fact, peak effects at a locus such as *Vita1a* are concentrated late in life (Fig. 1h,i).
2. *Loci with steadily diminishing effects* have uniform actuarial slopes from as early as 215 days that extend to the T_750_ survivorship. They are potential rate of ageing modulators (RAMs) using a liberal definition of ‘uniform slopes’. Seven have an almost age-independent hazard ratio (HR) over at least 500 days. Their effects converge towards minimal expectancy differences. *Vita14b* is an example in which the initially high difference in males between *CH* and *BD* genotypes fades from T_305_ to T_1,100_ (Fig. 2b). The effect is due to high mortality of *B* and *D* carriers up to 800 days followed by high mortality of *C* and *H* carriers thereafter (Extended Data Fig. 5).
3. *Loci with age-range restricted effects*. This tripartite category includes loci with action limited to early, middle or late survivorships (Fig. 2c–f).
  - *Early-age loci have effects before T*_*500*_. Loci of this type are three times more common in males than in females (Table 2). One of the best examples is *Vita1c*, for which the 50-day difference in life expectancies of carriers of *BH* and *BD* genotypes is restricted to males (Fig. 2c and Extended Data Figs. 2c and 3c1,c2). Exceptions are notable because polarities of genotype effects are flipped between sexes at four of these types of loci—*Vita2b*, *Vita9a*, *Vita11b* and *Vita18a* (Fig. 3c–f and Extended Data Fig. 2n,n1,s,s1,ab,ab1). The male bias in this type of locus is almost always linked to higher mortality rates in the T_200_ to T_700_ survivorships (Fig. 1a,b, Table 2 and Extended Data Fig. 4).
  - *Mid-survivorship loci effects from T*_*500*_ *to T*_*845*_. There are six loci of this type. *Vita4a* is an impressive example, with a biphasic fluctuation in mortality rates of *BD* and *CH* genotypes between T_500_ and T_710_ in males (Fig. 2e and Extended Data Figs. 2i and 3i1,3i2). This effect is caused by offset waves of mortality. A *D* haplotype wave of mortality starts at 400 days (vertical line in Fig. 2f) but is followed by *C* and *H* haplotype waves at 600 days. By 1,000 days both *B* and *D* haplotypes have high relative HR values approaching 2 (log_2_(HR) ≈ 1). The offsets in peaks of effects in actuarial plots (Fig. 2e) versus mortality plots (Fig. 2f) is because survivorships integrate mortality out to the final death. These waves are more obvious in Fig. 2f, and for all loci in Extended Data Fig. 4.
  - *Late-acting* Vita *loci effects after T*_*860*_. Five loci only act after T_860_ (Fig. 2d and Tables 1 and 2). As mice die the ratios of genotypes do shift but this does not affect mapping until about T_1,100_. Thereafter, genotype imbalance inhibited us from further mapping (Supplementary Table 2).
4. *Loci with reversals of genetic effects.* Twelve loci have effects that reverse across survivorships (Table 2). Reversals can involve a single genotype (the red *BD* trace in Fig. 2a) or two or more, for example *BD* and *CH* effects of *Vita4a* (Fig. 2e,f). These patterns are associated with age-dependent and highly variable HRs.

## Dynamics of heritability

**Extended Data Fig. 6:**
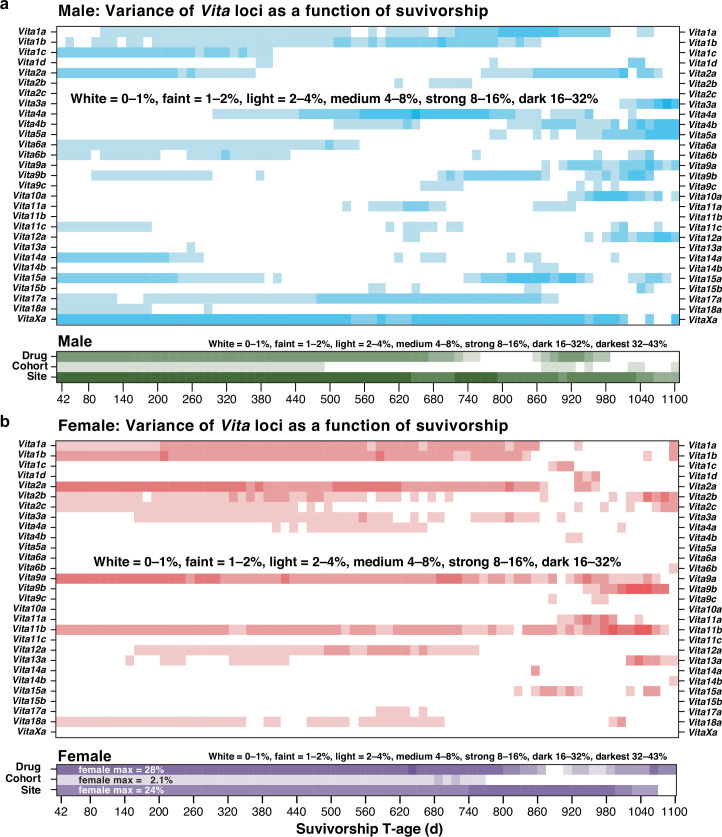
Dynamics and variance of 29 *Vita* loci as a function of sex and survivorship. **a,b**, Variance contributions of *Vita* loci were estimated for each survivorship and sex: males in **a** and females in **b** with intensity of colors indicating the approximate fraction of variance explained, where white = 0–1%, faint = 1–2%, light = 2–4%, medium 4–8%, strong 8–16%, dark 16–32%. Non-genetic experimental sources of variance (**Ve**) are provided in green for males and purple for females. **Drug** is variance attributable to multiple nominally ineffective supplementary drug treatments versus the standard chow diet. **Cohort** is variance attributable to the nine annual cycles of production of UM-HET3 mice from late spring through late fall between 2004 and 2013 (2008 was a hiatus year). **Site** is variance associated with the three ITP sites: The Jackson Laboratory, The University of Michigan, and University of Texas Health San Antonio.

The summed genetic variance explained by all 29 *Vita* loci on lifespan (*V*_g_) is higher in males than in females from T_42_ to T_320_ (Fig. 2g,h and Table 1) but drops from a base at 40% to 30% by T_500_ (Fig. 2g and Extended Data Fig. 5). This decline is due to the wave of early male mortality. By T_650_ male *Vita* heritability rebounds and climbs to 50% in the oldest survivorships. Female *Vita* heritability is almost precisely 27% in all survivorships to T_620_ but decreases to 20% by T_890_, 450 days after the minimum for males. As in males, female heritability recovers in the oldest survivorships, although by 10% less than in males. Variance explained by two-locus epistatic interactions also differs markedly by sex (*V*_g × g_ in Fig. 2g,h). This value is twice as high in females than in males, 12% versus 6%, and this is true over the entire reproductive lifespan. Note that the *V*_g_ (Fig. 2) is not a conventional narrow-sense estimate of heritability, but rather is variance explained only by the *Vita* loci and also will include dominance effects (Extended Data Fig. 6). Our results are consonant with the strong age-dependent increase in heritability noted in humans^18^ and higher sex-averaged heritability estimates when adjusting for extrinsic causes of death^28^.

We estimated the effect of three experimental sources of variance (*V*_exp_): (1) treatment with or without a nominally ineffective drug; (2) cohort year; and (3) three sites. Collectively, *V*_exp_ differs by sex and age (Fig. 2g,h). The summed level of *V*_exp_ peaks at 47% in males in the T_440_ survivorship but drops in rough synchrony with the end of reproductive life^29,30^ (Fig. 2g). Less than 20% of variance in older survivorships is linked to these non-genetic experimental sources—a counter-intuitive reduction in sensitivity. Despite efforts by ITP teams to standardize protocols, variance attributable to site is up to 43% in males and 24% in females (Fig. 2i,j). In males, variance associated with an ineffective drug is much more modest (Fig. 2i), although in absolute terms these supplements do increase lifespan at T_42_ by 40.2 ± 8.3 (mean ± s.e.m.) days. By contrast, in females, the effect of drugs is greater than that of site (Fig. 2j) and increases lifespan by 37 ± 6.7 (mean ± s.e.m.) days. This positive effect persists to T_680_ in males and to the T_800_ in females (Fig. 2i). However, it is negative by T_890_ in males (–11.7 ± 5.3 (mean ± s.e.m.) days, *P* = 0.03) and by T_1,040_ in females (–10.2 ± 5.3 (s.e.m.) days, *P* = 0.06). Other sources of variance (*V*_o_) (orange lines in Fig. 2g,h) include stochastic causes of mortality, for example uncontrolled environmental and technical factors, and higher-order genetic and G × E effects that we have not modelled, including age-dependent changes in survivors per cage and unmeasured indirect social genetic effects^31^.

## *Vita* loci have antagonistic sex interactions

The profound sex difference in life expectancies in early survivorships (Fig. 1b) can be decomposed into sets of loci that specifically influence G × S interactions and early male mortality. The likely cause is higher stress and aggression among males^32,33^. High male mortality is visible in plots for *Vita1c*, *Vita2a* and *Vita6b* (Fig. 2c and Extended Data Figs. 2–4). These and other loci have strong but transient effects earlier in the lives of males. This youthful mortality risk is eliminated in survivorships above T_725_ (Fig. 1b). From T_935_ to T_1,100_, males are typically housed solo and gain a small 8-day expectancy advantage over females (T_1,040_, *P* = 0.036).

Fourteen *Vita* loci have strong G × S interactions (Fig. 3a and Table 2). The complex of loci on chromosome 2 is an extreme example (Fig. 3a–d). *CH* and *BH* genotypes at *Vita2b* confer a lifespan advantage to females up to T_700_ (Fig. 3e), but a disadvantage to males through to T_365_. Polarities of effects are also reversed, with *CH* and *BH* having longer lifespans in females but shorter lifespans in males (Fig. 3c,d). This is due to a sex inversion in timing of sequential waves of mortality of carriers of the paternal haplotypes (Fig. 3f). *Vita2b* also has a massive G × S effect with a LOD of 8.6 at T_140_ that is entirely lost by T_860_ (Fig. 3a). The reversal of male genotype effects at *Vita2b* fits models of antagonistic pleiotropy (Fig. 3d)—genotypes that reduce mortality in the first half of life increase mortality after 600 days. The *Vita2* complex illustrates how mapping sexes together without fitting an interaction term is ill-advised. Although the sex-combined analysis defines highly significant peaks at T_800_ for *Vita2b* and *Vita2c* (Fig. 3b), this is an artefact of not modelling the interaction (Extended Data Fig. 1e,f). Similarly, G × S mapping unmasks two sex interactions at the extreme distal end of chromosome 4 (154 Mb) at *Vita4b*, a locus that is nominally only detected in males (Fig. 3a).

The male-specific *VitaXa* locus encompasses the entire proximal 70 Mb of chromosome X (Fig. 3g and Tables 1 and 2) with effects that erode almost linearly with survivorship T-age. This locus is likely to integrate net effects of a set of recessive variants on this hemizygous chromosome in males. In females, the effects of *C* and *B* haplotypes do not differ (Extended Data Figs. 1ac and 2ac).

## *Soma* loci balance body mass versus lifespan

**Extended Data Fig. 7:**
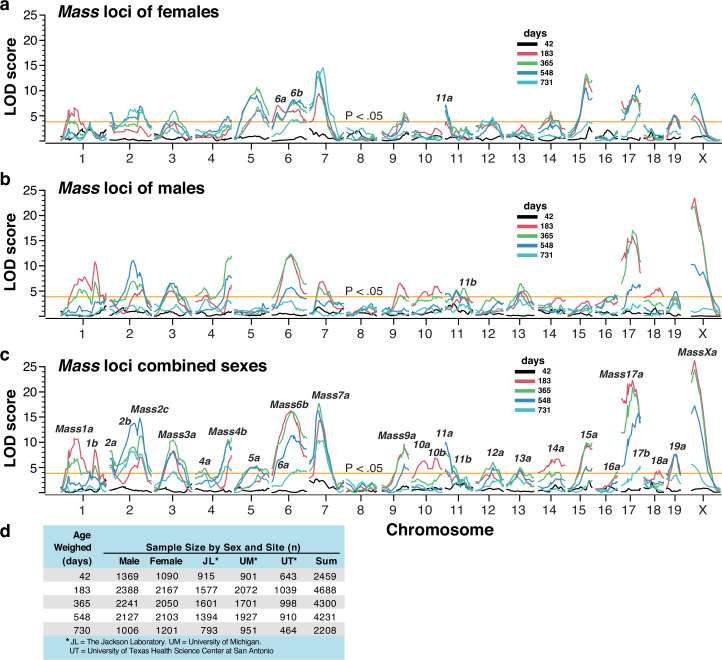
Body *Mass* loci at five ages. **a**, Female maps. **b**, Male maps. **c**, Combined maps in which all significant *Mass* loci have been labeled. Note that three of the 28 *Mass* loci are more distinct in the female map (*Mass6a*, *Mass6b*, and *Mass11a*) than in combined or male maps. *Mass11b* is more distinct in the male map. At all ages the mapping model compensates for drug treatment, cohort year, and site, and in the case of the combined sexes also adjusts for sex. Compare **(c)** with Fig. 5a, but note that color assignments are different here. **d**, Table of sample sizes for each sex and for the sites. Animals at 42 days were only weighed for the first four cohorts, accounting for the lower sample size. The lower sample size at 730 days is due in part to mortality.

Mice were weighed at 42, 183, 365, 548 and 730 days. Body masses at the first four ages correlate negatively with subsequent lifespans in both sexes (Fig. 4d–g) up until the oldest survivorships^34,35^. However, correlations differ greatly by sex—rank order rho values of –0.28 for males but only –0.11 for females at T_185_ (*P* < 0.001) (ref. ^36^). This translates to a loss of 14.3 days g^−1^ in males versus 3.7 days g^−1^ in females at the peak of reproductive performance (Fig. 4e). The sex difference remains significant to T_800_ (bold black linkage score trace in Fig. 4e). Female correlations are stable and remain close to –0.1 (Fig. 4e–h). By contrast, the negative correlation in males erodes with age and overlaps female values in older survivorships (T_890_; Fig. 4g,h) and then shifts to positive values in both sexes for body mass at 730 days (Fig. 4h). We investigated what loci account for these dynamic shifts in relations between body mass and mortality. Our first step was to map body mass loci at all five ages and evaluate their roles in mortality. We detected 28 *Mass* loci (Fig. 4a and Extended Data Fig. 7), but these are almost entirely independent of the *Soma* loci (Fig. 4a,b, note faint vertical lines) that modulate trade-offs between body mass and life expectancies.

**Extended Data Fig. 8:**
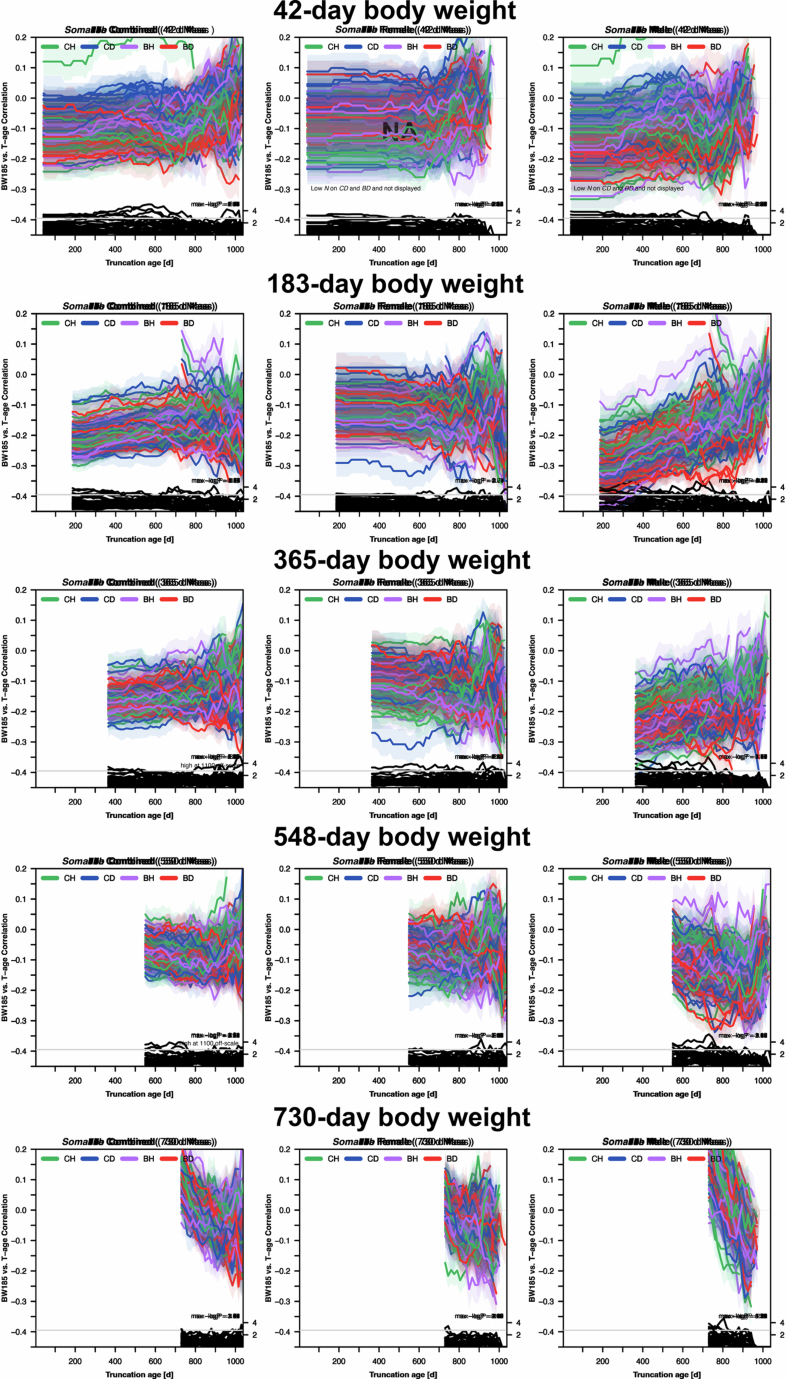
*Soma* actuarial effect size plots. **a**, An overlay of all 30 *Soma* loci and their full actuarial effect size plots at five ages at which body weights were measured as in Fig. 4i,j. This PDF should be opened in Adobe Illustrator or Inkscape to preserve the separation of the 30 layers (file also available at *files.genenetwork.org/current/umhet3_2025/*). With control of layers users can examine one or more of the 30 *Soma* loci, in the same way as in Fig. 4i–l but at all five ages at which animals were weighed. *Soma1a* is displayed in panel **a** and in the matched layer (upper left). *Soma19b* is displayed in panel **ad** and the matched layer. Actuarial correlation values are given in 15-day steps from the five ages at which mice were weighed. All technical details are provide in Fig. 4i–l.

Using correlated trait mapping^37^ we defined 30 *Soma* loci that modulate the negative and positive trade-offs between mass and mortality risk (Fig. 5b,c, Tables 3 and 4, Extended Data Fig. 8, Supplementary Tables 10 and 11). Fifteen *Soma* loci are detected only in males and four are detected only in females (Tables 3 and 4). Nineteen modulate the strength of negative correlations with body mass at 42 and 185 days. Eleven modulate the strength of positive correlations in the post-reproductive phase of life^30,38^ (Tables 3 and 4 and Extended Data Fig. 8). The effects of *Soma* loci on expectancies range from 2 to 29 days g^−1^. Table 4 presents these effects for each genotype both as day per gram and as differences in rank correlations. *Soma3b* has strong effects in males (Fig. 5i) but none in females (Fig. 5j). Males with the *CD* genotype lose 17.9 days g^−1^; those with the *CH* lose only 8.4 days g^−1^. In females, the differences in these negative correlations are insignificant (*CH*: –2.9 days g^−1^ versus *CD*: –3.4 days g^−1^). *Soma11a* has the strongest effect in females (Tables 3 and 4 and Extended Data Fig. 8), but effects are modest compared to those in males.

*Soma* loci overlap *Vita* and *Mass* loci (Fig. 4a–c and Table 4) at a chance level. The summed genome-wide coverage of *Soma* loci is 1,349 Mb (48% of the genome), and coverage of the *Vita* loci is 1,000 Mb (36% of the genome). Seven *Soma* loci have peaks within 10 Mb of *Vita* peaks (*P* = 0.19). *Soma1a* and *Vita1a* share top markers in both sexes, and both are modulated by the *H* and *D* haplotypes (Fig. 1f and Extended Data Figs. 3a and 8a). *H* lengthens life by 12 days, whereas *D* shortens life by 22 days. Surprisingly, effects of *Soma1a* oppose those of *Vita1a*, particularly in females. *H* decreases life expectancy by –4 days g^−1^ (average *ρ* = –0.12), whereas *D* increases expectancy by 1 days g^−1^ (rho = +0.02). All effect plots, for example those in Fig. 4m–p, are provided in Extended Data Fig. 8 for all loci, both sexes and T-ages.

## Epistasis is segregated by sex

We tested for epistatic interactions among the 59 *Vita* and *Soma* loci at four ages by sex with cut-off thresholds close to a LOD of 4.0, a value significant at a Benjamini and Hochberg^39^ FDR of 0.01. There are 41 significant *Vita*–*Vita* interactions among 387 *Vita* pairs that we tested in the base T_42_ survivorship using an even more stringent Bonferroni correction at *P* < 0.05 (Fig. 5a,b and Table 2)—22 in males and 19 in females. Across all four survivorships the number of *Vita–Vita* interactions is 59 in males and 43 in females (Supplementary Table 12.4). Similarly, there are 57 *Soma*–*Soma* interactions in males and 35 in females (Fig. 5a,b and Table 2). Average LODs in the four survivorships and two sexes range from 4.4 to 4.6 ± 0.5 to 0.8 (mean ± s.d.). One of the stronger male interactions is between *Vita1c* and *Vita3a* (Fig. 5d, LOD 5.0) in which effects of *BD* and *CH* genotypes at *Vita3a* produce differences of 100 days in lifespan across the *Vita1c* genotype*s*, but there is no corresponding interaction in females (Fig. 5d). However, *Vita3a* has a strong interaction with *Vita5a* in females as late as T_740_ but there is no corresponding interaction in males (Fig. 5f). The strongest female *Vita–Vita* interactions are *Vita1a–Vita6b*, *Vita3a*–*Vita5a* and *Vita1b*–*Vita9a* (Fig. 5a,b). The *BD* genotype at *Vita1b* masks any effects of *Vita9a*—the classic Mendelian definition of epistasis^40^ (Fig. 5e). Finally, there are 197 *Vita–Soma* interactions—84 in females and 113 in males—a bias that is expected from the greater numbers of male *Vita* loci (20 versus 8 in females) and male *Soma* loci (18 versus 8 in females). *Vita*–*Soma* loci have LOD scores of 4.5 ± 0.6 (mean ± s.d.).

**Extended Data Fig. 9:**
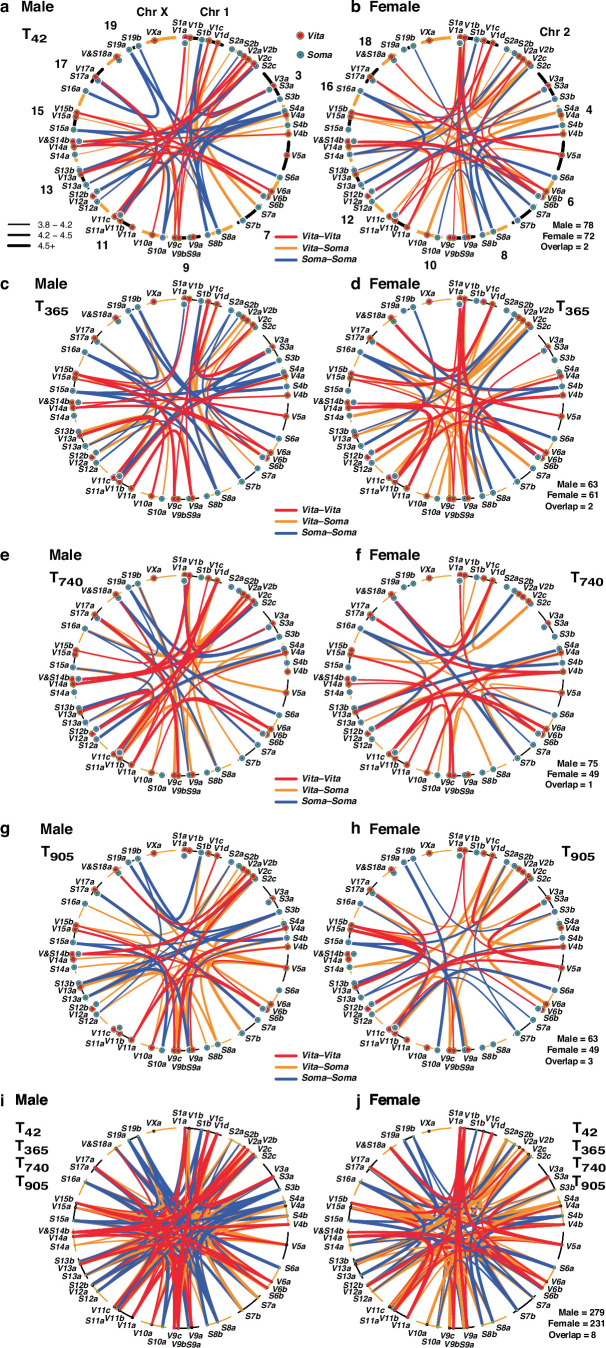
Epistatic interaction plots for the T_42_, T_365_, T_740_ and T_905_ survivorships. **a,g**, Overview of all epistatic interactions in survivorship using the same conventions. Chromosomes are labeled with abbreviated *Vita* and *Soma* symbols. Color and type of lines define partnership types (orange lines are *Vita*-*Soma* pairs). **i,j**, An overlay of all four survivorships that mainly highlights the greater cumulative numbers of male than female interactions. This pair of circle plots is a set of overlapping layers useful to directly compare different survivorships and the Adobe Illustrator format is available upon request. Number of epistatic links for both males and females is given in the lower right corner of each female plot. Few epistatic interactions overlap in both sexes. All values are given in Supplementary Table 12.

Epistatic interactions of *Vita* and *Soma* loci form segregated male and female networks (Fig. 5b,g,h and Extended Data Fig. 9). Even those few loci with minimal sex differences in their main effects do not share partnerships (Fig. 5a). For example, in females *Vita1a* pairs with *Vita6b*, *Vita9a* and *Vita11a* with LODs of 5.5, 4.6 and 4.7, respectively, but in males all of the corresponding LODs are marginal: 1.5, 3.2 and 3.5, respectively. No interactions are shared by *Vita* loci in the T_42_ survivorship (Fig. 5b), and only 2 out of 61 possible interactions of all 3 types are shared at the most lenient threshold in any survivorship (Extended Data Fig. 9 and Supplementary Table 12). The same pattern is true of *Soma*–*Soma* and *Vita*–*Soma* interactions. Polarities of interactions can even display sexual antagonism^41–44^; for example, the complementary effects of the *Vita2b CD* genotype (blue line) across *Vita1c* genotype columns (Fig. 5c). Although *Vita1c* does not have any main effect in females (Figs. 1e and 3a and Table 1), a test of epistasis exposes a strong interaction at this locus with *Vita2b* in females (Table 2).

Epistatic partnerships are stronger (Fig. 2g,h) and more stable in females than males as a function of age (Fig. 2g,h and Supplementary Table 12.3). For example, 21% and 17% of partnerships at T_42_ are matched at T_740_ and T_905_, respectively, in females, but only 9% and 0% are matched in males. Essentially all of these findings are consistent with a strongly sex-dichotomized genetic architecture—a form of genetic diplomacy that we presume harmonizes male and female phenotypes with divergent life history styles but necessarily convergent reproductive goals^45,46^.

## From maps towards mechanisms

As a first step, we assembled data on variants within each locus that are predicted to have an effect on protein function or with potential roles in ageing^47^ (Tables 1–4 and Supplementary Tables 1 and 13). We also conducted global enrichment analyses of loci with respect to Gene Ontology categories, KEGG (Kyoto Encyclopedia of Genes and Genomes) and Reactome networks (Supplementary Table 16). Although this process can help rank plausible candidate genes, there are hard limits. The two largest loci almost certainly encompass many variants. *Vita17a* extends from the centromere to 74 Mb (Table 1) and overlaps the major histocompatibility complex, which has been known since the 1970s to be linked to variation in lifespan^48^. *VitaXa* covers the proximal 70 Mb of chromosome X and is therefore an intractable oligogenic male-specific locus.

By contrast, *Vita1a* and *Vita9b* are already tractable. *Vita1a* is compact, overlapping only 14 protein-coding genes, of which *Mrpl15* (4.8 Mb), *Atp6v1h* (5.1 Mb) and *Rb1cc1* (6.2 Mb) are strong candidates involved in mitochondrial function, autophagy and nutrient sensing, respectively. ATP6V1H is notable, given its ubiquitous activity as a modulator of lysosomal acidification^49^ and mTOR function. Variants in this gene extend lifespan in *Drosophila*^50^ and *Caenorhabditis elegans*^51^ and modulate insulin secretion and type-2 diabetes risk in humans^52^. The unique data that we generated on epistatic interactions is mechanistically relevant because we now know that *Vita1a* interacts non-linearly with *Vita6b*, *Vita9a* and *Vita11a* in females but not in males. These are all strong constraint when ranking candidate genes for testing at all three loci (Fig. 6g,h).

*Vita9b* is mechanistically intriguing because it modulates mortality in both sexes in older survivorships, and also because it is a replicated locus that was previously mapped in a different cohort^53^ (Fig. 6a, inset). The *D* and *H* haplotypes at *Vita9b* reverse polarities of effects in males between T_500_ and T_700_, and there are strong mortality rate inflections in late survivorships (Fig. 6b and Extended Data Fig. 2o2). By contrast, *C* and *B* haplotypes have uniform effects that peak at T_785_ (Fig. 6c). In females, the effects are modest and are delayed to T_800_ (Extended Data Fig. 2o2). These age and sex differences are additional constraints in candidate ranking as shown below for alignment to human longevity genome-wide association studies (GWAS) data.

*Vita9b* spans 127 protein-coding genes with missense variants that are predicted to have moderate or high impact. We tested 11 *C. elegans* orthologues within *Vita9b* (*Acad11*, *Hyal1–3*, *Col6a6*, *Lrrc2*, *Ip6k2*, *Trim71*, *Poc1a*, *Stt3b*, *Pcbp4*, *Acp3* and *Apeh*) and 3 candidates just distal to the locus (*Slc22a13*, *Zfp445* and *Csrnp1*) using RNA interference (RNAi) knockdown (Fig. 6e–h). All but one of these genes have missense variants in UM-HET3 mice (Supplementary Table 14). Knockdowns of 4 candidate genes altered motility, a validated predictor of lifespan^54,55^, in aged worms older than 14 days (Fig. 6f,h). For example, suppression of *acds-10*, an orthologue of the mouse *Acad11* gene, boosted motility from 18 to 27 days with an effect comparable to the *daf-2* (*Igf1r* in mouse) positive control^56^ (Fig. 6f–h). In mice, *Acad11* is *trans*-activated by p53, enhances fatty acid β-oxidation, and increases expression with age^57–59^. This gene is a particularly high-rank candidate. Three other knockdowns reduced *C. elegans* motility after ten days (Fig. 6f–h): *pho-6* (*Acp3* in mouse), *pes-4* (*Pcbp4* in mouse) and *dpf-5* (*Apeh* in mouse); *APEH* is also well supported by human GWAS data on longevity.

To bridge the translational gap between mouse and human ageing^60,61^, we tested association between human orthologues of genes in *Vita1a* and *Vita9b* by Mendelian randomization^62,63^. In the case of *Vita1a*, we did not detect significant effects of any of the candidates we were able to test—*LYPLA1*, *MRPL15*, *PCMTD1* or *TCEA1*. In the case of *Vita9b*, we were able to test 7 out of 11 genes that we tested in *C. elegans* (Supplementary Table 14). *APEH* notably stood out. Variants in this gene are associated with parental longevity in humans (Benjamini–Hochberg –log*P* of 3.7 with paternal age at death) and with individual survival into the top 10% age group (Benjamini–Hochberg –log*P* of 1.37; Supplementary Table 15). Increased *APEH* expression in human blood is positively associated with male longevity, in line with the mouse genetics and our findings in *C. elegans*.

## Discussion

Most previous work on the genetics of ageing has defaulted to a single metric—the duration of lifespan. The actuarial method that we use tracks age-dependent mortality from puberty to senescence as a function of genotype and sex. The 59 *Vita* and *Soma* loci shape age-dependent mortality rates. Loci such as *Vita1c* and *Vita18a* act only early in life and are much more common in males than in females. They are likely to be linked to male competition, hormone status and stress resilience^36,64^. Other loci act almost exclusively from 500 to 700 days of age or affect only the oldest individuals. These late-acting loci are the prime target of longevity genetics using human centenarian cohorts^65–67^. A final subset has persistent effects on mortality across survivorships and may influence rates of ageing. The *Vita* and *Soma* loci form hundreds of epistatic interactions that are strictly segregated by sex, and ignoring sex or mapping without modelling gene-by-sex interactions is problematic. Uncovering these networks of age-dependent loci and their interactions provides an empirical genetic context for molecular, cellular, organismal and life history studies of senescence in mice and humans^2,4,6,26^. We are able to place loci into age-dependent mortality classes and to address their fit with three evolutionary explanations of ageing^19–21,27^.

The first is that senescence is caused by late-acting variants that escape selection in natural populations owing to high extrinsic causes of mortality—the mutation accumulation theory^19^. This idea predicts post-reproductive effects of common genetic variants that are both good and bad with respect to longevity, but more neutral during the reproductive phase of life. Effects of this type should only emerge at the onset of senescence; gradually, like *Vita5a* in males, or abruptly, as thresholds of resilience are breached later in life. Examples include *Vita1a* and *Vita11b* in females and *Vita5a* and *Vita10a* in males. The second more physiological explanation is that senescence is a consequence of a compromise between reproductive investment and somatic self-repair^21,27^. All early-acting *Soma* loci fit this disposable soma argument—they are genetic embodiments of a bioenergetic compromise between body mass at the height of reproduction and subsequent duration of life. The third explanation is that ageing represents a Faustian bargain that boosts fitness early in life, but calls for payment late in life—the antagonistic pleiotropy theory^2,20^. Loci such as *Vita2a*, *Vita2b*, *Vita2c* and *Vita9b* (Fig. 6b) are vivid examples that are consistent with this idea, in that genotype effects of loci on mortality invert with age. A simple prediction is that alleles that improve fitness before 400 to 600 days are linked to higher reproductive success but also to accelerated mortality. The dynamics of *Vita* and *Soma* loci provide a bridge between evolutionary explanations of senescence and genetic and molecular processes that account for progressive functional decline^68,69^.

We have defined 13 loci with strongest effects in males, 4 loci with strongest effects in females, and 12 loci with strongest effects when data from both sexes are pooled. This male bias is as pronounced among *Soma* loci as it is among *Vita* loci. Sex differences in the genetics of lifespan are amplified by their epistatic interactions, mirroring findings in *Drosophila*^70^. The divergence is so large that we are tempted to call this pattern sexual antagonism, although sexual diplomacy or compromise may be better terms. This genetic imprint is highest during the reproductive crescendo^33^, across the same age ranges that contribute to strong trade-offs between body mass and life expectancy that are accentuated by many male *Soma* loci^36^. After reproduction, natural selection is necessarily mute, and male and female life expectancies converge.

Our analysis of sources of variability that contribute to age-dependent mortality differences between the sexes is incomplete. The higher genetic variance attributable to *Vita* loci in males than in females was unexpected, and is reflected in greater success in detecting male *Vita* and *Soma* loci. By contrast, the higher variance due to experimental factors such as site and dietary additives does not yet have any pathological explanation (no wounding or sudden weight loss). Male–male interactions are a likely but unproven culprit. The loss of cage mates in older survivorships may explain the marked reduction in V_exp_ in males after T_560_ (Fig. 2g) and in females after T_800_. The relentless rise of other sources of variability (*V*_o_) probably reflects the increasing importance of hundreds of age-dependent frailty-associated loci that we have not modelled, as well as stochastic age-related diseases. This variance source must be the driver of the Gompertzian climb of mortality rates with age. The genetics of body mass coupling with lifespan is much stronger in males than in females, particularly in the younger survivorships. Interventions to blunt this coupling would therefore tend to be more effective if applied proactively early in life. By contrast, interventions that target geriatric cohorts—mice older than 800 days or humans older than 75 years—will probably have to contend with diverse drivers of escalating age-dependent diseases.

The ITP has tested pro-longevity effects of 62 drugs, about 25% of which have had positive effects. Less noted is a strong bias in favour of positive effects in males (updated to the 2021 cohort)^71^. Rapamycin is one welcome exception, with beneficial pro-longevity effects in both sexes even late in life. The loci that we have defined serve as anchor points to test compounds that modulate mortality and to determine whether they interact with known loci or define drug-specific loci as a function of sex and age. Our results can guide interventions to extend health and longevity^72–74^.

## Methods

### The UM-HET3 sibship

UM-HET3 mice are progeny of a cross between two types of F_1_ hybrids—female F_1_ mice from matings of BALB/cByJ dams to C57BL/6J sires and male F_1_ mice from matings of C3H/HeJ dams to DBA/2J sires. These four inbred progenitors are abbreviated CBy, B6, C3H and D2 when referring to mice and strains, and abbreviated *C*, *B*, *H* and *D* when referring to genotypes and haplotypes. These four fully inbred strains were selected to maximize phenotypic diversity. Young virgin F_1_ males and females bred at the Jackson Laboratory (JL) JAX facility were transferred to ITP ageing colonies at JL in Bar Harbor Maine, the University of Michigan in Ann Arbor Michigan (UM) and the University of Texas Health Science Center in San Antonio (UT). Breeding cages were set up in spring. First litters were not used. All subsequent litters were used at UT, litters of 6 or more pups were used at JL, and those with 7 or more pups were used at UM. Weanlings were entered into the study over the next 7–8 months.

This study is based on tail samples acquired initially at the three ITP sites in accordance with standards of the Association for the Assessment and Accreditation of Laboratory Animal Care and recommendations of the National Institutes of Health Guide for the Care and Use of Laboratory Animals, including annual reviews and approvals of all protocols. Links to data for ITP papers and data are available at the Mouse Phenome Database^75^.

All mice in the first four cohort years were born between April 2004 and January 2008. In these cohort years we have numerically well-balanced DNA samples from all sites. Almost no mice were generated in 2008 owing to a funding gap. All mice in the final cohort years used in this study were born between July 2009 and March 2013. The 2009 cohort includes mice from all sites, but 2010 and 2011 include mice only from UM and UT, and cohort years 2012 and 2013 include mice only from UM. The last UM-HET3 mouse in this study died in 21 December 2015. In all years, only a small percentage of mice (<10%) were born between January and April. Mice were weighed at 42 ± 2 days, and at 183 days (6 months), 365 days (12 months), 548 days (18 months) and at 730 days (24 months) with a timing error of ~7 days.

The UM-HET3 sibship segregates for ~10.6 million sequence variants (Supplementary Table 1). All UM-HET3 mice inherit *C* and *B* haplotypes from their F_1_ mothers and *H* and *D* haplotypes from their fathers. As a result, the entire sibship segregates for four genotypes, the four two-way combinations of maternal and paternal haplotypes, on all autosomes—*CH*, *CD*, *BH* and *BD*. Females inherit one entire non-recombinant *H*-type chromosome X from their paternal grandmothers and a potentially recombined chromosome X from their mothers (recombinations between *C* and *B* haplotypes only; Fig. 3g). As a result, females have either *CH* or *BH* chromosome X genotypes. Hemizygous males inherit a potentially recombined *C* or *B* chromosome X from their mothers. All mitochondria and their genomes in all animals are derived from maternal grandmothers (*C*) and male chromosome Y is derived from paternal grandfathers (*D*).

We genotyped 6,872 UM-HET3 mice for which we had full lifespan estimates (*n* = 3,252 females, *n* = 3,620 males). Of these genetic siblings 6,438 passed all genotype quality control steps at 891 markers (*n* = 3401 females, *n* = 3037 males; see Fig. 1c and ‘Genotype quality control’). To ensure balanced numbers by sex later in life, every ITP cohort initially consists of 51 male and 44 female weanlings per year, per site and treatment category, but with twofold more common untreated controls at each site. This was done to enable overly aggressive males and any wounded mice to be removed while balancing male and female numbers later in life—almost always before 550 days. The earliest minimum inclusion age in this study is T_42_, the pubescent age at which tails were docked for tissue acquisition, an age equivalent to about 12 years in humans. For numerical convenience we set the first T-age at T_35_. To compensate for twofold higher male mortality in the first 2 years of life (33% versus 16%), we included 12% more males than females in the T_42_ survivorship. The earliest death was at 46 days. This left us with a small surplus of 227 males at T_365_, but by T_560_, numerical balance was restored and there were 2,930 females and 2,929 males. This age corresponds to roughly 56 years of age in humans. All individual-level data, metadata, survivorships and mortalities per 15-day interval are provided in Supplementary Table 2.

We genotyped two major categories of mice—those not treated with any dietary intervention and mice treated with a dietary supplement that did not modify lifespan significantly on a per-drug basis using standard statistical criteria^76–78^. These latter mice have been referred to as ‘no drug effect’ (NDE) cases. However, when data are combined across the entire class of these individually ‘ineffective’ agents from 2004 to 2013, there is a highly significant combined effect that is positive on lifespan (Fig. 2i,j).

### Husbandry

Mice were weaned into same-sex cages—three males or four females per cage—at 20 ± 1 days (ref. ^22^). They lived together from weaning to death without any replacements within cages. As a result, most mice lived alone after ~1,000 days, a factor to consider with respect to mortality risks, but not yet integrated into our analysis. From 2004 until 2013, all sites used NIH-31 standard diets. For breeding cages, UM used Purina 5008, UT used Teklad 7912, and JL used Purina 5K52. For mice up to ~122 days, UM used Purina 5001, UT used Teklad 7912, and JL used Purina 5LG6. After 2004, a single control diet—LabDiet 5LG6—was used by all sites. Mice were monitored daily for signs of ill health and aggression and euthanized if moribund. Tails were obtained at 42 ± 2 days for DNA extraction. Body mass at this age was acquired for 2,459 mice, and at half-year intervals for 4,688 mice at 183 days (6 months), and down to 2,208 mice at 730 days (24 months). Our analysis includes four experimental variables—sex, site, dietary drug treatment and cohort year (Supplementary Table 3). The class of nominally ineffective treatments included in this study are listed on Mouse Phenome Database ITP Portal under the acronyms 4OHPBN, CAPEhi, CAPElo, Cur, Enal, FOhi, FOlo, GTE, HBX, I767d, MB, MCTO, MET, NFP, OAA, Res07, Reshi3, Reslo3, Simhi, Simlo and UA.

### DNA extraction, sequencing and sequence alignment

Tail tissue was processed using the MagMAX magnetic-bead extraction system and genomic DNA was quantified using a Qubit 4 fluorometer and a high-sensitivity DNA assay kit (Thermo Fisher Scientific). Locus-specific PCR primers were designed using BatchPrimer3^79^ to generate 180-bp amplicons. They were mixed using an optimized Hi-Plex approach at Floodlight Genomics. Sample-specific 12-bp barcodes were added and amplicons were pooled^80^. Primer sequences for markers with reference SNP rs identifiers are provided in Supplementary Table 4. The amplicons were sequenced to an average of ×1,000 per targeted DNA variant on an Illumina NovaSEQ (2× 150 bp reads). Data were demultiplexed and FASTQ files used for genotyping. FASTQ files were aligned to *M. musculus* GRCm38.p6/mm10 reference using Bowtie *2* (v2.3.4.1)^81^. BAM files were sorted and indexed using samtools (v1.6)^82^, and read group information was added using picard tools (v2.14.1).

### Variant calling

We called short sequence variants using bcftools^82^ in three steps: (1) we created text pileup output for all BAM files using mpileup with mapping quality of 30 or greater; (2) we called using default settings; (3) we removed variants with a read depth of less than 100 across all samples or a QUAL score of less than 100. Sequencing depth per case was used as an additional criterion for filtering reads. Sequence for C57BL/6J, C3H/HeJ and DBA/2J was downloaded from the Wellcome Sanger Mouse Genome Project^83^ (https://www.sanger.ac.uk/data/mouse-genomes-project) and that for BALB/cByJ was generated by Beierle and colleagues^84^. From these files we extracted variants that differ among the four founders (Supplementary Table 1). Variants were called in three steps as above, the differences being that the minimum depth was reduced to ×10 and QUAL score to ≥30. Variants that were called reliably in all founders were advanced for genotype phasing and mapping. VCF files with variant data on UM-HET3 and their founders are provided in Supplementary Table 5.

### Genotyping by Sequenom MassARRAY

Tails were placed in deep-well plates and sent in three batches to Neogen Corporation. Individuals were genotyped by SNP genotyping by Sequenom MassARRAY MALDI-TOF mass spectrometry^85^ at a maximum of 270 markers. Fifty markers were removed after quality control, based on deviations from expected allele frequency (0.35 < frequency < 0.65). We chose pairs of linked markers to define maternal and paternal chromosome genotypes.

### Phasing SNP genotypes

SNP genotypes were phased using R v4.0.2 (script at https://github.com/DannyArends/UM-HET3). We generated fully phased haplotypes for all sets of maternal and paternal autosomes and chromosome X. Markers fall into those that unambiguously define maternal haplotypes (*n* = 486, *C* versus *B* alleles and pink lines in Fig. 1c) and paternal haplotypes (*n* = 396, *H* versus *D* alleles and blue lines in Fig. 1c). We generated diploid genotype probabilities at all positions from phased chromosomes using the calc.genoprob() function in R/qtl. Markers were reviewed and those that had a call rate <30% were excluded. When we plot genotype or haplotype effects (Extended Data Figs. 2–5), we call genotypes and haplotypes when imputation certainty is greater than 80%. We used R/qtl to estimate number of recombinations per mouse. The mean ± s.d. value was 25.1 ± 8.6. Just over 370 animals had recombination numbers more than 2× s.d. above this value. We excluded 59 mice that had more than 80 recombinations, a value likely to be caused by sample cross-contamination. The final genotype files are available at GeneNetwork.org^86–88^ by setting Species = Mouse, Group = Aging Mouse Lifespan Studies (NIA UM-HET3), and Type = DNA Markers and SNPs and pressing the Info button. Supplementary Table 6 lists all genotypes.

### Genotype quality control

Markers and individuals with greater than 90% missing data were removed from further analysis (*n* = 333). We converted data from genotypes into parental haplotypes (see ‘Phasing SNP genotypes’). Markers were checked for Mendelian inheritance errors and removed when missing data after conversion exceeded 90%. We integrated MonsterPlex and Sequenom marker sets based on genomic positions. We inspected the marker map for linkage between neighbouring markers and removed a single marker (chr. 4_58784823) which did not show linkage to its neighbours. We also removed 20 individuals that died of unnatural causes and 22 that we inadvertently genotyped that had received an effective drug supplement. Finally, we constructed an R/qtl cross object for imputation using the remaining mice, imputed their haplotypes based on the full population (*n* = 6,438). Supplementary Table 7 shows the R/qtl cross object used for most analyses.

### Inversions in the UM-HET3

There is a large inversion on chromosome 6 of the C3H/HeJ progenitor strain^89^ between 51 Mb and 94 Mb which will affect regional map precision. The recombination fraction in this interval is 0.00025 versus an expectation of 0.21. At least 42 Mb of the paternal chromosome is locked in linkage. We detected a total of 13 potential inversions ranging in size of 3.5 to 54 Mb. Six suppress recombination on the maternal chromosome and seven suppress recombination on the paternal chromosome (Supplementary Table 8). There is overlap of maternal and paternal inversions on chromosomes 4, 7 and 11.

### Lifespan estimates depend on age of entry

Based on work by König and colleagues^38,90^, we know that in wild commensal populations of mice, 35–40% of pups do not survive beyond weaning. At birth, mean lifespan can be as low as 196 days. By 13 days, the mean expectancy has improved to 250 days for males and 500 days for females. We make these points to emphasize that lifespan estimates are made with reference to an operational starting point—a minimum ascertainment base age for a population. Researchers often incorrectly imply that mean lifespan is estimated from the date of birth, but this neglects earlier and often unknown numbers of deaths—referred to as left truncation. In our case we can only measure lifespan for UM-HET3 progeny that lived to be pubescent juveniles. When we say that the mean lifespan of the 6,438 sibs in this study is 844 ± 210 days (mean ± s.d., median of 870 days), we mean that this is the mean age at death conditioned on a mouse having lived to at least 42 ± 2 days—the age at which tail tips were taken for DNA extraction.

In a similar way we can determine the mean lifespan or life expectancy of the survivorship subsets of UM-HET3 mice that lived to be at least 185, 365, 545, 725 or 1,100 days (roughly 0.5, 1, 1.5, 2 and 3 years, respectively). The mean conditional lifespans of those five survivorships or age cohorts are 847 ± 206, 861 ± 188, 890 ± 160, 941 ± 126 and 1,162 ± 54 days (mean ± s.d.), respectively. The oldest mouse was a female that died at 1,456 days. We refer to these different ages at which lifespan can be computed as either survivorships or cohorts.

The actuarial procedure consists of sequentially studying progressive older and therefore smaller survivorships from the radix population of 6,438 mice in Fig. 1a. The minimum T-age is 42 days. Between T_42_ and T_50_ days a single male died. We truncated survivorships upward in 15-day steps from T_35_ to T_1,100_, a convenient step size for plots that can also be rationalized as roughly the range of survivorship T-ages (1,065 days) divided by the square root of the radix (*n* of 6,438). The first anchor point age was defined as 35 days in order to align plots at 365 days (1 year), 725 days (~2 years) and 1,100 days (~3 years) using 72 steps. We explored both right and bilateral truncation but do not cover the analyses here.

The actuarial mapping strategy is affected both by lower sample size and by right skew at higher truncations. To ensure that our results are not affected by this, we used three approaches. First, we use a non-parametric quantile regression in combination with standard parametric mapping to make sure associations are detected using both methods^91^. Second, we limited ourselves to a maximum T-age of T_1,100_. Third, we used a time-dependent hazard function to validate *Vita* loci.

We tested a complementary approach for mapping under the assumption that loci contribute to variation in the time-dependent hazard function as in our previous work^17^ but with refinements. We carried out a test at each marker of the null hypothesis that the (time-dependent) hazard function of death does not depend on genotypes. To do this, we used a model where the hazard function is allowed to depend smoothly on age with haplotype and baseline covariates, using natural splines with three degrees of freedom. For computational tractability we created risk sets with 50-day windows^92^.

### Implementation of actuarial mapping

We mapped in 15-day survivorship steps from T_42_ to T_1,100_. Mapping was performed using both conventional four-way mapping of genotypes at 891 markers (Fig. 1c) or by restricting analysis to each of the parental haplotypes—*C* versus *B* on the maternal chromosomes (*n* = 495 markers), and *H* versus *D* on the paternal chromosomes (*n* = 396 markers). Note that chromosome X only segregates for maternal *C* and *B* haplotypes in both sexes (Fig. 3g). In general, mapping all four genotypes provides better power. This method defined all loci in Tables 1 and 2 and full LOD scores across all survivorships are illustrated in Extended Data Figs. 1 and 2. The null model (model(H_0_)) and alternative model (model(H_alt_)) were fitted at each marker using the following linear models:

$${\rm{model}}({{\rm{H}}}_{0}):{\rm{lifespan}}={\rm{sex}}+{\rm{site}}+{\rm{cohort}}+{\rm{treatment}}+{\rm{error}}$$

$${\rm{model}}({{\rm{H}}}_{{\rm{alt}}}):{\rm{lifespan}}={\rm{sex}}+{\rm{site}}+{\rm{cohort}}+{\rm{treatment}}+{\rm{gtp}}+{\rm{error}}$$

Sex has two levels (M, F), site has three levels (JL, UT and UM), cohort year has nine levels (no animals were generated in 2008), treatment has two levels—untreated controls and cases that received a drug that had no significant effect on lifespan. The gtp (genotype probability) term is an *n* row by four-column matrix, where *n* is the number of individuals used in the mapping, and columns are genotype probabilities for markers computed by R/qtl (v1.48-1)^93,94^. Sex was dropped as a co-factor from the regression model when mapping was stratified by sex. LOD scores were computed by comparing the fit of the null model with the fit of the alternative:

$$\mathrm{LOD}=(n/2)\times {\log }_{10}(\mathrm{sum}(\mathrm{residuals}{({{\rm{H}}}_{0})}^{2})/\mathrm{sum}(\mathrm{residuals}{({{\rm{H}}}_{\mathrm{alt}})}^{2}))$$

Mapping on chromosome X requires special care due to the four-way cross structure and greater difficulty defining highly reliable markers for haplotype, particularly close to both telomeres.

### Actuarial effect size plots, their errors and interpretation

We use the four-way mapping algorithm implemented in R/qtl^94,95^ while controlling for sex, site, cohort year and drug treatment. We compute maps for survivorships starting from T_42_ up to T_1,100_ (Fig. 1a,d–f). We introduce a new type of actuarial plots in Fig. 1b,d,e. The *x* axis defines the minimum inclusion age (T-age) of each survivorship. The expected increase in error terms in older survivorships with lower sample sizes are mitigated by the reduced range over which animals die within progressively older survivorships—from 1,410 days at T_42_ to 356 days at T_1,100_. Errors of lifespans of survivorships are relatively stable—from 844 ± 210 (mean ± s.d.) in the T_42_ base population (s.d./range of 15%) to 1,162 ± 54.4 (mean ± s.d.) in the last T_1,100_ survivorship (s.d./range of 15%). When interpreting effect size plots note that increases and decreases in mortality rates among subgroups are relative to the mean lifespan of that T-age.

The T-age of a survivorship always refers to an entire age range. Actuarial truncation in a forward direction means that the oldest-old mice are embedded in every survivorship. Reverse truncation flips the polarity by truncating from oldest to youngest and defines exitships. The _1,099_T exitship consists of mice that died before 1,100 days and complements the T_1,100_ survivorship. The combination of forward, reverse and even two-sided truncations is useful to dissect potential causes of mortality in age-restricted subpopulations. In this paper we restrict attention to forward truncations.

### Significance testing for actuarial analyses

We evaluated the significance of mapping results across two axes: (1) the spatial location of markers and their independence across the genome; and (2) the temporal axis and the number of effectively independent actuarial tests using left truncation that progressively excludes mice that died before reaching the T-ages. We mapped using 891 markers but given the significant linkage between markers we effectively tested about 442 independent genome locations. This number of independent tests was estimated using the simpleM method with a block length ranging from 10 to 500 markers and a 10-marker step size^96^. The threshold at a genome-wide type 1 Bonferroni error rate of 10% is 3.65 LOD (–log_10_(0.1/442) at 5% it is 3.95, and at 1% it is 4.65. These are conservative thresholds. For maternal and paternal maps, the density of recombination is reduced by half, and corresponding thresholds are 3.44, 3.65 and 4.34.

### Locus confidence intervals

We used the standard 1.5 LOD drop to estimate the confidence interval of linkage^97^. We also estimated the temporal duration of action of loci, defined as the range of T-ages over which a locus has strong effects. The start and stop T-ages of these temporal confidence intervals are those that immediately precede or succeed survivorships that are genome-wide significant. For example, if linkage is above LOD of 3.95 from T_365_ to T_725_ survivorships, then the T-age confidence interval of the locus is from T_350_ to T_740_. This criterion is not conservative when only a few survivorships have significance. In this latter case, we defined the T-age range that brackets the peak T-age by at least a 1.5 LOD drop.

We evaluated the impact on significance testing of mapping multiple nested survivorships using a Cauchy combination test^98^ at each marker across all survivorships (Table 1). Cauchy *P* values were adjusted for multiple testing using a Benjamini and Hochberg correction to account for multiple testing across markers, and the Cauchy –log*P* in Table 1 of ≥1.50 is significant at a *P* of less than 5%.

### Locus names and ambiguities

Locus names are prefixed with *Vita*, *Soma* and *Mass* identifiers followed by chromosome and a letter suffix (for example, *Vita1a*, *Soma2b* and *Mass11c*). When the 95% confidence intervals of two adjacent QTLs overlap, and the genotype and haplotype effects appear to be the same, loci are generally assigned the same *Vita* identifier. For such ‘merged loci’, the 95% confidence interval is reduced to the overlapping interval. Determining whether a locus is detected in different subsets of the sibship is somewhat subjective, examples being *Vita3a* and *Vita14b* that could have been split into two loci by sex and T-age. We provide data for linkage scores in all three categories (sexes combined, females and males) for *Vita* loci (Supplementary Table 9), *Soma* loci (Supplementary Table 10) and *Mass* loci in (Supplementary Table 11).

### Testing for sex interactions

There are significant differences between male and female stratified survivorship maps. To investigate this formally and to detect significant sex-marker interactions modulating age-specific lifespan we tested sex interactions of *Vita* loci at the T-age with the peak LOD (Fig. 3a). We computed the LOD score difference of two models against each other:

$${\rm{model}}({{\rm{H}}}_{0}):{\rm{longevity}}={\rm{sex}}+{\rm{site}}+{\rm{cohort}}\,{\rm{year}}+{\rm{treatment}}+{\rm{gtp}}+{\rm{error}}$$

$${\rm{model}}({{\rm{H}}}_{{\rm{alt}}}):{\rm{longevity}}={\rm{sex}}+{\rm{site}}+{\rm{cohort}}\,{\rm{year}}+{\rm{treatment}}+{\rm{gtp}}+{\rm{gtp}}:{\rm{sex}}+{\rm{error}}$$

Since we are only interested in significant sex interactions at previously detected *Vita* loci (excluding only *VitaXa*) we used a Bonferroni correction based on 28 markers. A LOD score above 2.75 is significant (–log_10_(0.05/28)).

### Testing pairwise epistatic interactions

A two-dimensional analysis of marker-marker interactions affecting life expectancies was performed by testing the following models against each other in four survivorships—T_42_, T_365_, T_740_ and T_905_.

$${\rm{model}}({{\rm{H}}}_{0}):{\rm{lifespan}}={\rm{sex}}+{\rm{site}}+{\rm{cohort}}\,{\rm{year}}+{\rm{treatment}}+{{\rm{gtp}}}_{{\rm{m}}1}+{{\rm{gtp}}}_{{\rm{m}}2}+{\rm{error}}$$

$${\rm{model}}({{\rm{H}}}_{{\rm{alt}}}):{\rm{lifespan}}={\rm{sex}}+{\rm{site}}+{\rm{cohort}}\,{\rm{year}}+{\rm{treatment}}+{{\rm{gtp}}}_{{\rm{m}}1}+{{\rm{gtp}}}_{{\rm{m}}2}+{{\rm{gtp}}}_{{\rm{m}}1}:{{\rm{gtp}}}_{{\rm{m}}2}+{\rm{error}}$$

We computed the difference in fit between H_0_ and H_alt_ as explained in ‘Implementation of actuarial mapping’. To limit numbers of tests, we initially tested interactions between the 29 *Vita* loci (Table 2), excluding all pairs of loci on the same chromosome (Fig. 5a). This resulted in the matrix of 387 non-syntenic tests of pairs of loci per sex. We used Bonferroni corrections for most work. With 387 *Vita*–*Vita* tests, a LOD of approximately 4.0 is appropriate, but for more comprehensive tests of all loci in all 4 survivorships (11,768 tests) the corresponding *P* < 0.05 threshold is 5.37, a value that limits the yield of interactions to only 50 (Supplementary Table 12). By contrast, the Benjamini and Hochberg test suggests that 966 interactions have FDR of less than 0.005 (Supplementary Table 12.2). We opted for a compromise and analysed a total 289 unique epistatic interactions that are visualized in part in Fig. 5g,h for the T_42_ survivorship, and much more comprehensively Extended Data Fig. 9 for all 4 survivorships. All interactions have LODs greater than 3.8. Of a total of 11,768 tests fewer than 4% have LODs ≥ 3.8 (Supplementary Table 12).

### Visualizing genetic modulation of mortality rates

We plotted age-dependent mortality and the HRs of haplotypes to give more insight into the specific ages over which genetic differences operate with more or less force. We computed mortality rates of maternal and paternal haplotype pairs (*C* versus *B*, *H* versus *D*) for males and females separately. We used LOESS smoothers with span parameters of 0.2 (Extended Data Fig. 4), and adaptive LOESS with a span of 0.3 (refs. ^99–101^) with shorter spans over ranges with high numbers of deaths (800–950 days) and longer spans over ranges with few deaths (Fig. 2f). log_2_ HRs greater than 0.6 represent relatively strong genetic effects (HR > 1.5 or HR < 0.67 in Fig. 2f).

### Body mass versus lifespan analysis—the *Soma* loci

Body masses were measured at 42 days (right before tails were biopsied for genotyping); and at 183, 365, 548 and 731 days (6, 12, 18 and 24 months). We used these data to map loci for body masses for both sexes so that we could align and compare body mass loci with *Vita* and *Soma* loci (for example, Fig. 5a–c). Sample sizes for mapping *Mass* loci range from a low of 2,459 at 42 days to a high of 4,688 at 183 days (Supplementary Table 11.1). Models used are like those used for mapping *Vita* loci, but the variable is body mass at each of five ages.

### Actuarial analysis of *Soma* loci

To investigate the effects of body mass on lifespan, we adjusted body mass and lifespan using the model:

$$\begin{array}{l}{{\rm{Lifespan}}}_{({\rm{adjusted}})}={\rm{mean}}({\rm{Lifespan}})+{\rm{residuals}}({\rm{Lifespan}} \sim {\rm{Sex}}\\ \,+{\rm{Site}}+{\rm{Cohort}}+{\rm{Treatment}})\end{array}$$

$$\begin{array}{l}{{\rm{BW}}}_{({\rm{adjusted}})}={\rm{mean}}({\rm{BW}})+{\rm{residuals}}({\rm{BW}} \sim {\rm{Sex}}\\ \,+{\rm{Site}}+{\rm{Cohort}}+{\rm{Treatment}})\end{array}$$

Where BW is body mass. This model includes mice for which we have lifespan and body mass data at one or more of the five weight ages. We computed the sex-stratified Spearman rank order correlations between body mass at 42, 183, 365, 548 and 730 days with lifespan in 15-day increments (Fig. 4d–p). We compute the –log*P* of the difference in correlation between males and females using the cor.test function in R. The average correlation in the combined population between adjusted body mass at 183 days (~6 months) and adjusted lifespan is rho = –0.206. When computed for males, correlation is stronger (rho = –0.284), while it is weaker for females (rho = –0.110).

### CTL mapping

We used correlated trait locus (CTL) mapping^37^ to determine if a distinct set of loci modulate correlations between body mass and subsequent lifespan (Tables 3–4). Before using this procedure, we adjusted body mass and lifespan using sex, site, cohort year, and drug treatment as covariates. At each marker we stratified survivorships based on genotypes. Ideally, the sample size for each subgroup would be above 400, but in some cases, *N* was as low as 200 due to genotype uncertainty and to the lower sample sizes at T_42_ (*N* = 2549) and T_730_ (*N* = 2208). We computed rho correlations for each genotype with *n* > 100 and the *z*-scores associated with differences:

$$z=0.5\times \log ((1.0+\rho )/(1.0-\rho ))$$

Here cor is a four-value vector containing the observed correlations for *CH*, *BH*, *CD* and *BD* genotypes. We compute the sum of squares (sumOfSq) by multiplying the observed sample sizes (ss) of the *CH*, *BH*, *CD* and *BD* genotypes to the squared z-scores:

$${\rm{sumOfSq}}={\rm{sum}}({{\rm{ss}}}^{* }{{\rm{z}}}^{2})$$

We compute the squares of sums (sqOfSum):

$${\rm{sqOf}}{\rm{Sum}}={\rm{sum}}({{\rm{ss}}}^{* }{{\rm{z}}}^{2})$$

Using these values, and the sum of the sample sizes on which each correlation is based, we compute the critical value (Cv) which follows a chi-square distribution under the null hypothesis that all correlations (*z*-scores) are from the same distribution:

$${\rm{Cv}}={\rm{sumOfSq}}{\rm{\mbox{--}}}({\rm{sqOfSum}}/{\rm{sum}}({\rm{ss}}))$$

The Cv is converted to a *P* value using the chi-squared distribution using the pchisq() function in R, with *P*[*X* > *x*] and three degrees of freedom (number of genotypes – 1).

We adjust the significance threshold for multiple tests using the p.adjust function in R at a 5% FDR (refs. ^39,102^). The 5% FDR threshold is approximately 2.75 –log*P*. While still stringent, this threshold is less harsh than that applied to *Vita* loci that use a highly conservative Bonferroni correction. Our rationale for this difference is our use of conservative non-parametric rho for comparing correlations of body mass to lifespan. Differences in correlation correspond to days gained or lost relative to an average individual that can be converted to effect sizes measured in days gained or lost per gram of body mass. We computed the linear regression coefficient for body mass on lifespan for the four genotypes as:

$${\mathrm{Lifespan}}_{(\mathrm{adjusted})}=\mathrm{mean}(\mathrm{Lifespan})+\beta \times {\mathrm{bodyweight}}_{(\mathrm{adjusted})}$$

where mean(Lifespan) is the intercept of the total population (all genotypes combined), while the *β* coefficient is the estimated effect size of body mass on lifespan based on the subpopulation defined by each genotype (Table 4). This leads to effect sizes relative to an averaged mouse. While there are caveats with respect to interpreting these effects because they are computed relative to this mean, these values give readers a sense of the impact of *Soma* loci on life expectancies.

### Broad-sense haplotype-based heritability

We compute broad-sense genotype-based heritability by fitting a full model including the haplotype probabilities of the 29 top markers of all *Vita* loci:

$$\begin{array}{l}{{\rm{lifespan}}}_{({\rm{T}}-{\rm{age}})}={\rm{sex}}+{\rm{site}}+{\rm{cohort}}+{\rm{treatment}}+{{\rm{gtp}}}_{{Vita}1a}\\ \,+{{\rm{gtp}}}_{{Vita}1b}+\ldots +{{\rm{gtp}}}_{{VitaXa}}+{\rm{error}}\end{array}$$

Broad-sense haplotype-based heritability is estimated by fitting this model to all survivorships. When stratifying by sex, sex is dropped from the model as well as from the *H*^2^_e_ component described in the computation of environmental variance component below. Computing heritability is done by taking the following approach, adapted from Falconer^103^ and Lynch and Walsh^104^ using five steps:

Step 1: The vector of partial total variances (PTV):

PTV = (*σ*^2^ – *σ*^2^_residuals_)/*r*

Step 2: An adjustment factor adj—the sum of the mean sum of squares for the fixed effect including the residual variance:

adj = sum(*σ*^2^)/*r*

Step 3: The contribution of each parameter in PTV to the model:

*C*^2^_p_ = PTV/adj

Step 4: The total broad-sense genotype-based heritability:

*H*^2^_h_ = *C*^2^_*Vita1a*_  + *C*^2^_*Vita1b*_ + _…_ + *C*^2^_*VitaXa*_

Step 5: The environmental variance:

*H*^2^_e_ = *C*^2^_sex_ + *C*^2^_site_ + *C*^2^_cohort_ + *C*^2^_treatment_

where *σ*^2^ is the vector containing all the means of sums of squares (including the residual) computed by the ANOVA model. *σ*^2^_residuals_ is the residual mean sum of squares. *r* is the average number of replicates for each genotype. *H*^2^_h_ can be interpreted as the broad-sense genotype-based heritability as a sum of all *Vita* loci. *H*^2^_e_ is the environmental variance estimate of the known environmental covariates. Unexplained variance *H*^2^_u_ can be computed as:

$${{H}^{2}}_{{\rm{u}}}=1-({{H}^{2}}_{{\rm{h}}}+{{H}^{2}}_{{\rm{e}}})$$

To obtain upper and lower bounds on *H*^2^_h_ and *H*^2^_e_ we generated 50 bootstrap resamples and fit the ANOVA model to each using a random subset of 90% of the survivorship. We computed median and standard deviations across all bootstraps to estimate errors of *H*^2^_h_ and *H*^2^_e_ as a function of survivorship T-age.

### Candidate genes analysis

All annotated features located within the 95% confidence interval were downloaded using BioMart^105^. We applied several criteria to prioritize positional candidate genes: (1) first, we considered only the protein-coding genes that reside in the 95% confidence intervals; (2) we then selected genes with annotated non-synonymous SNPs segregating in the population, and these were further ranked by the potential deleterious effect based on the ENSEMBL variant effect predictor^106^; and (3) another priority score for the candidate genes was based on whether they were listed as ageing and longevity genes in GenAge^107^. We also compared our positional candidate genes against known human, *C. elegans*, *D. melanogaster* and *Saccharomyces cerevisiae* genes reportedly associated with age in GenAge. To accomplish this, we use biomaRt to convert mouse gene symbols to the corresponding species-specific orthologous gene symbols. Supplementary Tables 13 and 14 provide access to all gene models and variant types segregating in *Vita* and *Soma* loci.

### Gene set over-representation analysis

We analysed gene set enrichment in *Vita* and *Soma* loci using R. Gene annotations were retrieved from Ensembl via biomaRt, retaining only mouse protein-coding genes, but excluding olfactory receptor genes (Olf*, *n* = 1,427), vomeronasal receptor genes (Vmn*, *n* = 595), predicted genes (Gm*, *n* = 1,246) and RIKEN cDNA sequences (*n* = 490). For each locus we tested set over-representation against our filtered whole-genome background (*n* = 18,830) using clusterProfiler for Gene Ontology (Biological Process, Molecular Function, Cellular Component), KEGG pathways and Reactome pathways. An adjusted *P* cut-off of 0.2 was applied to capture marginally significant enrichments for subsequent analysis. Results are summarized in Supplementary Table 16.

### *C. elegans* candidate gene screening of *Vita9b*

Out of 262 protein-coding genes in *Vita9b*, we highlighted 98 with missense variants and a subset with *C. elegans* orthologues using Ortholist2^108^ (Supplementary Table 16). From these, we selected a subset of 15 genes (Fig. 6g) represented in the Ahringer *C. elegans* RNAi library^109^ for testing: (1) *acds-10* (orthologue of mouse *Acad11*); (2) *chhy-1* (*Hyal1*, *Hyal2* and *Hyal3*); (3) *oct-1* (*Slc22a13*); (4) *col-135* (*Col6a6*); (5) *frm-8* (*Frmpd1*); (6) *let-413* (*Lrrc2*); (7) F30A10.3 (*Ip6k2*); (8)* lin-41* (*Trim71*); (9) *lis-1* (*Poc1a*); (10) *stt-3* (*Stt3b*); (11) *C41D11.3* (*Csrnp1*); (12) *znf-782* (*Zfp445*); (13) *pes-4* (*Pcbp4*); (14) *pho-6* (*Acp3*); and (15) *dpf-5* (*Apeh*). We also screened using the L4440 empty vector control and *daf-2* (*Igf1r*) as positive control. The sequence-verified single RNAi clones were inoculated in 10 ml lysogeny broth (LB) with ampicillin (100 μg ml^−1^) and tetracycline (50 μg ml^−1^) overnight at 37 °C. The next day, 5 ml of LB with 0.5 mM isopropyl β-D-1-thiogalactopyranoside was added and inoculants were incubated for 30 min at 37 °C with shaking for pre-induction. The bacterial inoculants were centrifuged (3,700 rpm for 15 min) and the pellet was resuspended in 10 ml liquid NGM with 50 µg ml^−1^ IPTG, 100 µg ml^−1^ ampicillin and 13.32 mg ml^−1^ nystatin.

Gravid adult stage TJ1060 [*spe-9(hc88)*;*rrf-3(b26)*] *C. elegans* were synchronized by bleaching. Resulting L_1_ stage worms were suspended in concentrated heat-inactivated OP50 bacteria in liquid NGM and transferred into U-bottom 96-well plates in a final volume of 100 µl. The worms were grown on an orbital shaker (Heidolph Titramax 1000) at 600 rpm until they reached the L4 stage (2.0–2.5 days) at 25 °C to prevent progeny production. When worms reached L4 stage, 20 µl of bacterial inoculant of each RNAi clone were added into single wells of the U-bottom 96-well plates containing worms. At least 30 worms per well and 8 wells per clone were used for two independent screens. The MicroTracker (InVivo Biosystems) system was used to measure motility in liquid in 96-well plates by infrared beam breaks. Plates were measured daily for a period of at least 90 min. Significance of differences in activity were estimated using the AUC (Fig. 6f) for either the full lifespan (1–30 days relative lifelong motility, Fig. 6g) or only after 14 days (Fig. 6h) using a two-tailed *t*-test of control-normalized AUC values assuming unequal variance and using a Bonferroni correction for 15 tests.

### Mendelian randomization analysis

We used Mendelian randomization methods to rank the translational relevance of genes within *Vita* loci against their human orthologues. We extracted lists of protein-coding genes within *Vita1a* and *Vita9b* with significant human *cis-*expression quantitative trait loci (*cis*-eQTLs) in blood from eQTLGen (31,684 individuals)^110^ as exposures (Supplementary Table 15). As outcome variables, we used summary statistics on parental longevity^111,112^ from the IEU OpenGWAS project^113^ and on human longevity^114^ from the GWAS Catalog^115^. We tested for potential causal effects of variation in expression on: (1) extreme longevity of at least one parent (>95 years of age, IEU OpenGWAS.io trait ebi-a-GCST003395)^111^; (2) both parents in the top 10% of longevity (IEU OpenGWAS.io trait ebi-a-GCST006698)^112^; (3) mother’s age at death (IEU OpenGWAS.io trait ebi-a-GCST006699)^112^; (4) father’s age at death IEU OpenGWAS.io trait (ebi-a-GCST006700)^112^; (5) mean parental age at death (IEU OpenGWAS.io trait ebi-a-GCST006702)^112^; (6) individual survival into the top 10% (GWAS Catalog: GCST008598)^114^; and (7) individual survival into the top 1% (GWAS Catalog: GCST008599)^114^. In many instances, multiple *cis-*eQTLs were detected in the GWAS summary statistics, allowing them to be used as instrumental variables. Because linkage disequilibrium pruning would leave only a small number of independent variants, we applied an established principal component analysis-based approach that aggregates correlated instrumental variables into linkage disequilibrium-based principal components (principal component instrumental variables)^116^ that were evaluated using the 1000 Genomes Project panel^117^ with PLINK^118^ v1.90b6.21. We retained top principal components explaining more than 99% of genetic variance and used the inverse-variance weighted method to test for potential causal effects. When only a single instrumental variable was available for both the exposure and the outcome, or when principal component analysis yielded a single component, causal effects were estimated using the Wald ratio method implemented in TwoSampleMR (v0.6.2)^63^. For associations that were significant, we additionally performed linkage disequilibrium pruning and conducted a leave-one-out sensitivity analysis, sequentially removing each independent variant and estimating effects of the remainder. We evaluated heterogeneity of estimates using Cochran’s *Q* test, and horizontal pleiotropy was tested. In this analysis we were sensitive to key assumptions of Mendelian randomization analysis^62,119^. The assumption of relevance is reasonably well satisfied but the assumptions of independence and exclusion are only partially satisfied. However, in the context of using Mendelian randomization methods to rank candidate genes in which negative findings are as informative as positive findings, this procedure improves bidirectional translation between mouse and human studies of mortality and longevity.

### Reporting summary

Further information on research design is available in the Nature Portfolio Reporting Summary linked to this article.

## Online content

Any methods, additional references, Nature Portfolio reporting summaries, source data, extended data, supplementary information, acknowledgements, peer review information; details of author contributions and competing interests; and statements of data and code availability are available at 10.1038/s41586-026-10407-9.

## Supplementary information

Supplementary Tables This zipped file contains Supplementary Tables 1–16, including a guide to the tables.

Reporting Summary

Tables 1 & 2 This file contains full versions of Tables 1 and 2 presented in the main article, which include the main and Extended Data figure call-outs.

Tables 3 & 4 This file contains full versions of Tables 3 and 4 presented in the main article, including the Extended Data figure call-outs.

Peer Review File

## Data Availability

All primary data used in this study are provided in the supplementary tables. We also provide all lifespan and body mass data for animals used in this study and for an additional 17,858 UM-HET3 mice (total of 24,296 ITP mice born up to 20 October 2020) at the URL: https://genenetwork.org/show_trait?trait_id=10001&dataset=HET3-ITPPublish along with tools for truncation, mapping and analysis of correlations between variables such as lifespan and body mass. All figures and supplementary tables are deposited at https://aging.genenetwork.org/UM-HET3 and https://files.genenetwork.org/current/umhet3_2025/. All published data of the ITP is also made openly available at the Mouse Phenome Database ITP Portal at https://phenome.jax.org/projects/ITP1/.

## Code availability

Code used in mapping and analysis and scripts used to generate all figures and extended data figures are available at https://github.com/DannyArends/UM-HET3. Supplementary files and original figures in Adobe Illustrator format are available at https://files.genenetwork.org/current/umhet3_2025/.
