# Supplementary material for: Dynamics of genetic and somatic trade-offs in ageing and mortality
Source: Nature. 2026 Apr 22;654(8118):437–53. doi: 10.1038/s41586-026-10407-9 (PMC13253337; doi:10.1038/s41586-026-10407-9)
Supplement: Supplementary file 1 — This zipped file contains Supplementary Tables 1–16, including a guide to the tables. [file 41586_2026_10407_MOESM1_ESM.zip › Supplementary Tables_Arends_10Apr2026/Tables_3-4_Combined_Soma_22Nov2025..pdf]

	Locus	Max. –Log*P* Any T-age @	High –Log*P* at Age Weighed †	High T-Age §	Sex Effects*	Effect Range (d/g)	Max. –Log*P* Any T-age †	Actuarial Range (d) ∆	*Soma* Positions (Mb, GRCm38) ¥ Chr	Prox	Peak	Distal	Size	Marker ID	SNP ID	Days Gained or Lost per Gram at High T-Age ◊ *CH*	*BH*	*CD*	*BD*	Correlation of Weight with Lifespan (rho) ◊ *CH*	*BH*	*CD*	*BD*	*Mass* Overlap *Mass* Locus Overlap §	*Mass* Locus Type and Sex §	*Vita* Overlap *Vita* Locus Overlap §	Locus Type and Sex §	Extend Data Fig. 3
1	***Soma1a***	3.95	3.42	185	C	8.3	4.0	≤365	1	0.0	3.0	24.0	24.0	1_3010272	rs31443144	-10.2	-11.8	-3.5	-4.5	-0.23	-0.26	-0.09	-0.12			***Vita1a***, *1b*	Durable (C)	a
2	***Soma1b***	4.09	3.85	730	M	4.3	4.1	≥730	1	63.9	86.2	133.6	69.7	1_86216552	rs240615003	5.1	1.4	0.9	5.2	0.32	0.07	0.08	0.32	*Mass1b*	Early (M)	*Vita1c*	Early (M)	b
3	***Soma2a***	3.56	3.50	185	M, C	10.4	3.6	≥183	2	0.0	13.6	24.6	24.6	2_13600088	rs29825025	-11.4	-21.8	-14.4	-16.1	-0.22	-0.43	-0.29	-0.38	***Mass2a***	Early (C)			c
4	***Soma2b***	5.07	3.12	42	C	47.4	5.1	≤548	2	30.4	60.2	83.9	53.5	2_60201233	rs29953805	7.3	2.7	-40.1	-30.2	0.07	0.03	-0.23	-0.23					d
5	***Soma2c***	5.09	2.77	42	M	30.8	5.1	42	2	148.4	161.9	180.1	31.6	2_161871392	rs27303276	-13.9	-13.1	-5.8	-36.6	-0.09	-0.09	-0.05	-0.34			*Vita2c*	Mid (F,M)	e
6	***Soma3a***	3.53	2.76	550	M	5.5	3.5	42, ≥548	3	42.2	88.0	108.2	66.0	3_87974845	rs36895924	1.3	-1.0	-3.7	-4.2	0.03	-0.05	-0.12	-0.17	***Mass3a***	Durable (C)	***Vita3a***	Early, Late (M)	f
7	***Soma3b***	4.93	3.95	185	M	9.5	4.9	All	3	121.5	159.6	159.6	38.1	3_159581164	rs30858154	-8.4	-13.7	-17.9	-16.7	-0.18	-0.29	-0.38	-0.34					g
8	***Soma4a***	4.02	3.32	42	M	20.0	4.0	All	4	0.0	11.2	30.8	30.8	4_30761996	rs27779705	-15.5	-31.9	-14.1	-34.1	-0.13	-0.28	-0.11	-0.22	*Mass4a*	Early (M)			h
9	***Soma4b***	3.55	3.15	42	C, M, F	20.4	3.6	≤183, ≥730	4	66.8	107.4	156.0	89.2	4_107374161	rs28147090	-12.9	-25.1	-4.7	-18.6	-0.13	-0.17	-0.02	-0.18	*Mass4b*	Durable (M)	*Vita4b*	Mid (M)	i
10	***Soma6a***	3.99	3.60	550	C, F	3.1	4.0	All	5	0.0	8.0	25.9	25.9	6_8006720	rs49698565	-2.9	-2.4	0.2	0.1	-0.15	-0.13	0.00	0.01					j
11	***Soma6b***	3.46	3.13	730	F	4.0	3.5	≥730	6	66.8	138.7	139.6	72.8	6_138658041	rs30021501	-2.2	0.3	0.4	1.8	-0.17	0.02	0.04	0.12			***Vita6b***	Early (M)	k
12	***Soma7a***	5.27	4.75	365	M	7.6	5.3	≥183	7	4.3	16.1	43.5	39.2	7_16072018	rs32395309	-14.5	-6.9	-10.2	-7.0	-0.38	-0.20	-0.26	-0.15					l
13	***Soma7b***	3.84	3.79	550	M	7.5	3.8	≥183	7	85.1	120.1	145.3	60.2	7_120086292	rs31151709	-2.0	-4.9	2.6	-4.8	-0.09	-0.17	0.06	-0.17					m
14	***Soma8a***	3.07	2.92	365	M	6.9	3.1	≤365	8	41.1	71.7	86.0	44.9	8_71684276	rs33469281	-8.3	-5.3	-12.2	-10.4	-0.22	-0.12	-0.33	-0.27					n
15	***Soma8b***	3.48	3.13	42	C	22.9	3.5	All	8	86.0	111.3	129.1	43.1	8_126505019	rs33182145	-11.9	-13.9	-28.5	-5.6	-0.11	-0.11	-0.25	-0.03					o
16	***Soma9a***	2.93	2.92	185	C	6.6	2.9	183	9	20.0	51.1	58.1	38.1	9_51116640	rs13461391	-13.0	-6.4	-10.0	-9.0	-0.30	-0.17	-0.23	-0.22			*Vita9a*	Early (F)	p
17	***Soma10a***	4.49	3.85	185	C,M	7.1	4.5	≤183, ≥730	10	0.0	18.1	90.1	90.1	10_18144599	rs29381732	-9.8	-9.0	-3.8	-10.9	-0.25	-0.22	-0.09	-0.25	*Mass10a*	Early (M)	*Vita10a*	Late (M)	q
18	***Soma11a***	4.22	3.31	365	F	3.6	4.2	All	11	50.4	97.4	113.7	63.3	11_97448477	rs45809946	-0.1	-3.7	-2.3	-2.0	0.03	-0.18	-0.15	-0.10	***Mass11c***	Durable (M)	*Vita11b*	Late (F)	r
19	***Soma12a***	3.88	2.97	185	F	6.1	3.9	183	12	58.3	71.7	79.1	20.8	12_71677220	rs29155467	-1.2	-7.3	-4.5	-2.8	-0.05	-0.24	-0.16	-0.08	***Mass12a***	Durable (F)			s
20	***Soma12b***	2.91	2.91	730	M	4.2	2.9	≥183	12	79.1	118.2	118.2	39.1	12_118179607	rs30534543	4.8	3.6	0.6	2.1	0.26	0.28	0.03	0.13			***Vita12a***	Mid (C)	t
21	***Soma13a***	3.52	2.80	42	M	46.0	3.5	≤183	13	4.4	19.4	67.3	62.9	13_19367506	rs50623363	12.3	-33.7	-25.1	-21.2	0.04	-0.28	-0.17	-0.21					u
22	***Soma13b***	4.71	3.22	42	F	25.0	4.7	42	13	77.3	98.5	111.4	34.1	13_98521647	rs30068968	-1.5	-22.3	2.7	-19.3	-0.06	-0.25	0.01	-0.11			*Vita13a*	Early (F M)	v
23	***Soma14a***	3.12	3.12	550	M	7.1	3.1	183 – 548	14	20.7	31.0	31.0	10.3	14_30957748	rs30464161	-0.1	-4.7	-0.8	2.4	-0.02	-0.17	-0.06	0.07					w
24	***Soma14b***	3.89	3.88	42	M	49.7	3.9	42	14	79.4	101.4	120.3	40.9	14_101437466	rs49624430	-45.8	-6.9	3.9	-18.1	-0.35	-0.07	0.06	-0.12	***Mass14a***	Early (C)	***Vita14a***	Early Mid (C)	x
25	***Soma15a***	4.50	4.34	185	M	16.4	4.5	183 – 548	15	0.0	3.3	20.7	20.7	15_3288506	rs31623892	-7.1	-3.6	-20.0	-13.5	-0.15	-0.09	-0.43	-0.31					y
26	***Soma16a***	3.86	2.76	550	C, F	4.3	3.9	≥548	16	38.0	75.8	96.4	58.4	16_75758401	rs46542250	-5.1	-4.8	-9.1	-8.4	-0.15	-0.13	-0.26	-0.22	***Mass16a***	Early Mid (M)			z
27	***Soma17a***	2.97	2.87	730	F, C	2.6	3.0	183F, 548M, 730F	17	0.0	26.5	68.8	68.8	17_26542857	rs33475140	-0.8	-1.1	1.5	1.1	-0.06	-0.09	0.14	0.09	*Mass17a*	Time Diff (MF)	***Vita17a***	Durable (M)	aa
28	***Soma18a***	4.68	3.30	42	C	24.0	4.7	42M, 42–365F	18	21.1	52.5	70.8	49.8	18_52488251	rs45936103	-0.9	-13.2	-24.9	-14.5	0.02	-0.10	-0.24	-0.16			***Vita18a***		ab
29	***Soma19a***	3.32	3.32	730	M	4.4	3.3	730	19	0.0	3.4	11.6	11.6	19_3403302	rs31128510	4.1	-0.3	3.9	3.2	0.27	-0.02	0.21	0.21					ac
30	***Soma19b***	3.00	3.00	730	M	5.1	3.0	730	19	35.0	53.9	62.0	27.0	19_53851357	rs30416321	4.3	-0.8	3.0	2.8	0.23	-0.04	0.22	0.18	***Mass19a***	Early Mid (M)			ad

@ **Max –LopP Any T-age** is the maximum value across any actuarial scan from any of the five ages at which mice were weighed. This value was **not** used to establish significance.

† **High –Log*P* At Age Weighed** and Max. –Log*P* Any T-age are the highest scores corresponding to 1. one of the five ages at which mice were weighed or 2. the highest –logP at any T-age Values are genome-wide significant at *P* < .05 with BH correction at values ≥2.75.

§ **High T-Age** is the T-age at which mice were weighed that gave the **High–Log*P* When Weighed** *value*. If the **High T-age** is 730, then all values correspond to the T730 surviorship for the first listed **Sex Effects**.

* **Sex Effects** where **C** = combined, **F** = female, and **M** = male given in the order corresponding to data for **High –LogP** and **HIgh T- Age**.

∆ **Actuarial Range** in days associated with *Soma* loci. When two age ranges are given they apply in order of **Sex Effect**s. Soma15a

¥ ***Soma*** chromosomes (**Chr**) and **Positions** using GRCm38 coordinates. **Prox** and **Distal** are proximal and distal marker positions. **Size** of locus in **Mb** (megabases).

◊ Effects sizes for genotypes at the **High T-Age** and first **Sex Effects** group**.** Green, blue, beige shading for combined, male, or female data.

§ ***Mass*** **Locus Overlap** and ***Vita*** **Locus Overlap** with the *Soma* locus. If **bold** then peaks are within 10 Mb. **Vita Type and Sex** from Table 1, abbreviated.

◊ **Days Gained or Lost** effects sizes for ***CH***, **BH**, ***CD***, and ***BD***)genotypes at the **High T-Age** for first **Sex Effects** group**.** Similar values for **Correlation of Weight.** Green, blue, beige shading for combined, male, or female data.
