# Supplementary material for: Dynamics of genetic and somatic trade-offs in ageing and mortality
Source: Nature. 2026 Apr 22;654(8118):437–53. doi: 10.1038/s41586-026-10407-9 (PMC13253337; doi:10.1038/s41586-026-10407-9)
Supplement: Supplementary file 1 — This zipped file contains Supplementary Tables 1–16, including a guide to the tables. [file 41586_2026_10407_MOESM1_ESM.zip › Supplementary Tables_Arends_10Apr2026/Tables_1_2_Combined_Vita_Loci_01Oct2025.pdf]

Table_1a_Vita_Summary

				Vita Positions (Mb, GRCm38) ¥							Survivorship T-Ages ∆						Effect Sizes			Sex Effects*			Epistatic G×G Effects by Sex			Survivorship Summary
	Locus •	Peak LOD @	Cauchy –logP ^	Chr	Prox	Peak	Distal	Size (Mb)	Peak SNP ID	Marker ID	From	Peak T-Age	To	Dura-tion	Max *N at Peak* §	Mean Life § (d)	Peak Var Male %	Peak Var Fem %	Max Peak Diff (d)	Sex Effects *	G×S at Peak LOD †	Max G×S at any T-age $	*Locus*	Interactions in Male ◊	Interactions in Female ◊	Type and Age of Main Effects **
1	***Vita1a***	6.84	3.64	1	0.0	3.0	8.0	8.0	rs31443144	1_3010274	42	860	935	893	3377	1002	5.7	3.4	30	C, F, M	0.78	1.40	***Vita1a***	11c	**6b**, **9a**, **11a**	Durable, Late Reversal (M F)
2	***Vita1b***	7.91	3.83	1	16.0	24.0	31.1	15.1	rs32645812	1_24042124	42	695	845	803	5056	930	4.4	2.2	37	C, F	0.78	0.78	***Vita1b***		6b, **9a**, **9b**	Durable, Late Reversal (M F)
3	***Vita1c***	5.51	3.14	1	101.4	121.5	148.6	47.2	rs30776698	1_121483290	42	230	335	308	3255	810	3.0	0.0	52	M	2.41	2.62	***Vita1c***	***3a***, 11c	2b	Early (M)
4	***Vita1d***	5.47	3.05	1	151.7	167.1	176.3	24.6	rs32557046	1_167148678	42	230	335	293	3379	810	0.9	0.0	54	M	2.28	2.28	***Vita1d***	**2c**, **9b**, **9c**	2b, 15b, **18a**	Early (M)
5	***Vita2a***	5.54	3.14	2	71.8	89.2	110.9	39.0	rs227254817	2_89156987	42	140	275	233	1548	807	2.9	5.6	70	M, F, X	**9.78**	9.78	***Vita2a***	13a, 14a		Early, Reversal (M), RAM (F)
6	***Vita2b*** •	6.84	3.51	2	110.9	112.7	130.9	20.0	rs27463377	2_112255823	575	800	890	315	4079	970	0.5	1.1	20	F, C,X, M	0.35	8.62	***Vita2b***	3a, **14b**	1c, 1d, 6a	Reversal (M), RAM (F)
7	***Vita2c***	8.13	4.61	2	132.8	148.4	158.4	25.6	rs29880912	2_148442635	42	545	665	623	2930	906	0.0	1.2	30	F, C, X	2.65	7.31	***Vita2c***	**1d**		Reversal (M), RAM (F)
8	***Vita3a*** •	4.26	1.92	3	42.2	83.8	121.5	79.2	rs13477232	3_83354281	1055	1070	1100	45	368	1146	7.5	0.0	35	M, X	**3.07**	3.07	***Vita3a***°	**1c,** 2b	**5a**	Early, RAM, Rev (M), Durable (F)
9	***Vita4a*** •	9.00	4.84	4	38.9	52.5	63.3	24.3	rs13477705	4_52524395	42	650	710	668	2588	908	8.2	1.6	53	M, C, X	**2.98**	3.58	***Vita4a***			Mid, Reversal (M), Durable (F)
10	***Vita4b***	4.43	2.38	4	132.4	154.3	156.0	23.6	rs250153474	4_154254581	500	635	680	180	2652	900	2.5	0.0	33	M, X	**3.30**	3.64	***Vita4b***	**14b**	**12a**	Mid (M)
11	***Vita5a***	4.05	2.10	5	36.6	67.6	90.2	53.6	rs3659098	5_67573068	965	1085	1100	135	350	1149	8.0	0.0	36	M, X	**2.85**	2.91	***Vita5a***		**3a**, 17	Late (M)
12	***Vita6a***	4.14	2.93	6	97.3	107.4	122.3	25.0	rs38831890	6_93680853	42	140	560	518	3396	807	2.2	0.0	38	M, C	1.74	1.74	***Vita6a***	14a	2b, 11b	Early, Mid (M)
13	***Vita6b***	5.27	3.05	6	108.1	132.8	149.7	41.7	rs31845771	6_132762500	42	320	515	473	3296	823	2.6	0.0	47	M, X	**2.79**	2.79	***Vita6b***	**13a**, 15a	**1a**, 1b, 14a, 14b	Early, Mid, RAM (M)
14	***Vita9a***	5.68	3.75	9	13.4	29.9	44.7	31.2	rs38493199	9_34932404	42	95	725	683	2754	910	0.9	5.0	35	F, C, M, X	**5.80**	5.80	***Vita9a***		**1a**, **1b**	Early (M), Early, Mid, RAM, Rev (F)
15	***Vita9b*** •	6.66	3.28	9	95.3	104.1	116.5	21.2	rs30513860	9_104091597	935	1025	1055	120	1847	1112	6.5	9.0	24	C, F, M	1.21	2.11	***Vita9b***	**1d**, **17a**	**1b**	Early, Reversal, Late (M), Late (F)
16	***Vita9c***	5.79	3.20	9	108.1	124.1	124.4	16.3	rs49693645	9_124056586	920	980	1070	150	1990	965	1.1	1.4	20	C, M, X	2.73	2.73	***Vita9c***	**1d**, **17a**		Early, Reversal, Late (M), Late (F)
17	***Vita10a*** •	4.48	2.48	10	56.7	72.8	100.5	43.7	rs13480647	10_72780332	965	980	1010	45	1812	1078	5.8	0.2	23	C, M	0.86	1.33	***Vita10a***	14a		Late (M)
18	***Vita11a***	4.21	2.20	11	0.0	5.6	30.1	30.1	rs26911247	11_6599922	560	635	695	135	2652	901	3.4	0.0	35	M	1.86	1.86	***Vita11a***			Mid Reversal (M)
19	***Vita11b***	4.07	1.96	11	59.0	82.2	109.4	50.4	rs234364394	11_82176894	995	1040	1055	60	586	1114	0.0	9.1	29	F, X	1.86	3.45	***Vita11b***		**1a**, 14a	Durable, Late Reversal (F)
20	***Vita11c***	4.91	2.91	11	97.9	113.7	119.3	21.3	rs29454463	11_113729074	42	42	290	248	3332	806	1.7	0.2	44	M, X	**4.41**	4.71	***Vita11c***	1a, 1c, 13a		RAM (M)
21	***Vita12a*** •	5.22	3.01	12	99.6	112.9	120.1	20.6	rs33465836	12_112855820	530	635	695	165	5475	910	1.5	2.5	27	C	0.03	0.94	***Vita12a***		**4b**	Durable (M,) Durable (F)
22	***Vita13a***	5.62	3.36	13	47.9	86.8	114.9	67.0	rs29246040	13_83858506	42	230	410	368	6203	863	1.4	1.7	33	C, M	0.29	1.68	***Vita13a***	2a, 6b, 11c		RAM (M), Mid (F)
23	***Vita14a***	6.08	3.61	14	62.6	70.8	101.4	38.8	rs51512690	14_78415875	42	42	290	248	6438	844	2.8	1.2	48	C, M	1.62	1.69	***Vita14a***	2a, 6a,10a,17a	6b, 11a, **11b**	Early (M), RAM (F)
24	***Vita14b*** °	4.86	2.87	14	78.4	101.4	120.3	41.9	rs49624430	14_101437466	42	80	290	248	3377	1006	2.1	0.4	18	C, M	0.22	1.80	***Vita14b***	**2b**, **4b**	6b	RAM (M), RAM (F)
25	***Vita15a***	5.08	2.89	15	55.5	74.2	88.4	32.9	rs45900875	15_74248242	785	905	950	165	3549	1023	5.7	1.9	21	C, M, X	1.36	3.25	***Vita15a***			Early, Mid, RAM (M), Durable (F,C)
26	***Vita15b***	5.73	3.14	15	99.3	102.2	104.0	4.7	rs31838876	15_99306167	42	95	275	233	3398	813	1.3	0.0	40	M, X	**4.11**	4.11	***Vita15b***	6b	1d	Early, Mid, Late (M)
27	***Vita17a***	4.97	3.07	17	0.0	32.9	73.7	73.7	rs6348789	17_32883804	440	665	740	300	5286	925	3.8	1.3	23	C, M	0.77	2.24	***Vita17a***	**9b**, **9c**,11b, 14a	5a	RAM (M)
28	***Vita18a***	4.56	2.73	18	33.2	52.5	81.0	47.8	rs45936103	18_52488251	42	140	455	413	6431	858	1.5	1.6	28	C	1.49	1.63	***Vita18a***		**1d**	Early (M), RAM (F)
29	***VitaXa***	5.64	3.28	X	0.0	36.0	69.8	69.8	rs13483724	X_36008085	42	260	485	443	3332	806	6.6	0.0	35	M	NA	NA	***VitaXa***			RAM (M)

• **Loci** with **filled circle**s were detected in Ref. 21 and replicated here**.** *Vita3a* has an early effect but linkage is significant only late in males. **Open circle**: The highest LOD for *Vita14b* is at T860 in combined data, but see Fig. 2b for early peak LOD in males.

@ **Peak LOD** values are significant at *P* of .01, .05, and .10 with LODs of 4.65, 3.95, and 3.65, respectively**.**

^ **Cauchy –logP** values are significant at ≥ 1.5**.**

¥ ***Vita*** chromosomes (**Chr)** and **Positions** in megabases using GRCm38/mm11 assembly. **Peak** is the marker or imputed position with the highest LOD at **Peak T-age. Prox** and **Distal** are proximal and distal marker positions. **Size** of locus in **Mb** (megabases)**.**

∆ **Survivorship T-Ag**es in 15-day truncation steps over which *Vita* loci are regarded as having an impact on lifespan centered around the **Peak T-Age** that gave the **Peak LOD** for the first-listed **Sex Effects** entry**.**

§ **Max *N* at Peak** is the survivorship sample size at the ***Vita* Peak LOD** at the **Peak T-Age.** For example, for Vita1a this number is 3377 mice at T860 which had a **Mean Survival** of 1002 days.

% **Peak Var Male** and **Peak Var Fem** are percentages of variance a locus accounts for in the survivorship with the **Peak LOD.** See Extended Data Fig. 3 for variances explained at all T-ages in both sexes.

* **Sex Effects** associated with the high linkage scores: male (**M**), female (**F**), combined (**C**), and G×S effect (**X**). The order is given by LOD score from high to low. **Peak LOD** only applies to the first **Sex Effects** entry.

† **GxS LOD. Bold** values are significant with Bonferroni corrections at ≤.05 with LOD of ≥2.75 when tested at the **Peak LOD** marker and **Peak T-Age**.

$ **Max G×S LOD** in any T-age survivorship

◊ **Epistatic G×G Effects by Sex:** *Vita* loci are isted with sufixes at LOD ≥3.8. **Bold font** for LOD ≥4.5**.**

** **Type and Age of Main Effect**s depend on sex as noted. See **Results** for explanations of *categories* and caveats. **Durable**: Effects to ≥T770; **RAM**: Gradual effect reduction to ≥T800; **Rev**: Effect reversals; **Early**: ≤T500, **Mid**: T500 to T845, **Late**: ≥T860.

° ***Vita3a*** and ***Vita14b*** are RAM type loci with two T-ages with high LOD scoes. ***Vita3a*** has an early effect but LODs are significant only late in males. The highest LOD for ***Vita14b*** is at T860 in combined data, but see Fig. 2b for early peak LOD in males.
