# Supplementary material for: Dynamics of genetic and somatic trade-offs in ageing and mortality
Source: Nature. 2026 Apr 22;654(8118):437–53. doi: 10.1038/s41586-026-10407-9 (PMC13253337; doi:10.1038/s41586-026-10407-9)
Supplement: Supplementary file 1 — This zipped file contains Supplementary Tables 1–16, including a guide to the tables. [file 41586_2026_10407_MOESM1_ESM.zip › Supplementary Tables_Arends_10Apr2026/__Supplementary Information Guide.docx]

**Supplementary Information Guide for Arends et al. 2026 (Nature_526217_4)**

**Supplementary Item Description**

Supplementary Data Table 1 All known protein-coding genes in *Vita* and *Soma* loci with information on chromosomal positions, length of gene model, numbers and types of sequence variants segregating in the UM-HET3 family, variant impact predictions, and an overview from GenAge of the potential modulation of variants in each gene on lifespan in three model organisms and in humans. This file is available in both Excel and CSV formats. Supplementary Data Table 13 provides a somewhat finer-grained breakdown by variant type.

Supplementary Data Table 2 Data on the number of deaths in each 15-day actuarial window (truncation step) for all 29 *Vita* loci for males and females separately and combined by genotype (*CH,* *CD*, *BH*, *BD*). We include columns on numbers of animals alive at each truncation age extending from 5 to 1490 days of age. We provide data on numbers of animals genotyped and total number of individuals at each truncation age. The truncation age ranges used in this study start at T-42 days and extend to T-1100 days. Note that in these tables the first T-age that we used is labeled “35–50”. The first two rows (5-20, and 20-35) are buffer rows that do not differ in content from the 35-50 row, since no animals were entered into the study earlier than the age at which their tails were docked for DNA acquisition. This file is a zip file that when uncompressed converts to a folder of three TXT files for each of 29 *Vita* loci.

Supplementary Data Table 3 Effects (beta values) and significance levels of covariates (sex, site, cohort year, drug treatment) used in mapping with levels of covariates.

Supplementary Data Table 4 Primers used for MonsterPlex genotyping. Note that a few MonsterPlex amplicons defined useful secondary markers close to the primary target variant.

Supplementary Data Table 5 A table of 1165 markers and 6873 UM-HET3 mice that were genotyped using a conventional VCF SNP calling format. This table consists of 6873 columns corresponding to all animals we genotyped and 1165 rows corresponding to all markers that we genotyped. A subset of 891 markers and 6438 mice passed all quality control stages.

Supplementary Data Table 6 The final filtered set of 891 genotypes that passed all QC steps and that were used for all mapping analyses, provided here in a single Excel table with encoding of genotypes at each marker that are appropriate for mapping a 4-way cross. While there are 4 primary genotypes there are a total of 14 possible genotype states that are used by R/qtl in imputing genotype and haplotype probabilities when mapping using a 4-way cross.

Supplementary Data Table 7 Cross-object file used for mapping with R/qtl version 1. This file can be opened and used for 4-way cross mapping with R/qtl (not R/qtl2). This is the cross-object file used in GeneNetwork.org for on-line mapping.

Supplementary Data Table 8 Listing of 13 large inversions that appear to be segregating in UM-HET3 based on suppression of recombinations over unexpectedly long physical distances. Positions given in megabases with observed and expected recombination fractions and χ^2^ values. Only one of these inversions has been reported in the literature (Ref. 89).

Supplementary Data Table 9 *Vita* loci mapping results at all T-ages in a set of four companion Excel tables in one Excel workbook. We provide the LOD scores of linkage for all animals combined (main effect), males (main effect), females (main effect), and the sex interaction effect. These tables all use conditional formatting so that linkage peaks are highlighted by green colors. Each column is a marker (“Chr_bp” values) and each row is a T-age. The first truncation age is T-35, the last is T-1100.

Supplementary Data Table 10 *Soma* loci mapping results expressed as −logP values of linkages. We also include the correlations of each of the four genotypes with subsequent mean lifespan at all five T-ages at which body weights were measured. We provide one Excel workbook consisting of five tables corresponding to each age at which body weights were measured.

Supplementary Data Table 11 Mass loci mapping results (sex-adjusted) at five ages in one Excel table.

Supplementary Data Table 12 Epistasis mapping results for all *Vita* and *Soma* loci at all T-ages. Data are provided in a single Excel workbook with one Read-Me table and 12 tables summarizing epistatic interactions by sex and age. Metadata for each table provided in header section.

Supplementary Data Table 13 DNA variant subtypes and their counts in *Vita* and *Soma* loci (finer detail than that provided in Supplementary Data Table 1).

Supplementary Data Table 14 *Vita9b* locus gene list used to select the subset of candidate genes to test in *C. elegans* (Fig. 6e).

Supplementary Data Table 15 Human Mendelian randomization analysis of primary target loci (*Vita1a* and *Vita9b*), as well as of *Vita9a* and *Vita6b* (epistatic partners of *Vita1a*).

Supplementary Data Table 16 Whole protein-coding genome gene ontology (GO), KEGG, and Reactome gene set enrichment analysis of genes within individual *Vita* and *Soma* loci. This Excel workbook consists of three sheets for the three top level GO categories, and separate sheets for networks of genes defined by KEGG and Reactome.
