# Supplementary material for: Dynamics of genetic and somatic trade-offs in ageing and mortality
Source: Nature. 2026 Apr 22;654(8118):437–53. doi: 10.1038/s41586-026-10407-9 (PMC13253337; doi:10.1038/s41586-026-10407-9)
Supplement: Supplementary file 5 — Peer Review File [file 41586_2026_10407_MOESM5_ESM.pdf]

nature portfolio

Peer Review File

Dynamics of genetic and somatic trade-offs in ageing and mortality

Corresponding Author: Professor Robert Williams

Any redactions in this file are there to maintain patient confidentiality, the confidentiality of unpublished data, or to remove third-party material.

This file contains all reviewer reports in order by version, followed by all author rebuttals in order by version.

Version 0:

Reviewer comments:

Referee #1

(Remarks to the Author)
Arends and coauthors report genetic mapping of lifespan and body weight in a large cohort (>6000) of genetically heterogeneous mice. They employ a novel (to this field) actuarial approach that enables the characterization of variation in the genetic effect size and direction across the lifespan. Using this approach they map 29 lifespan loci (Vita) that show a high degree of age- and sex-specific heterogeneity. Using another novel analysis strategy, the authors map 30 loci that modify the age-specific correlation between body weight and lifespan (Soma). They further report ~300 epistatic interactions among the Vita and Soma loci which underscores the complexity of the genetic regulation of lifespan.

These finding, based on a well powered study design, demonstrate that the genetic contributions to lifespan, direct and indirectly through body weight effects, involve many active loci with age, sex and context specific effects. While this has been commonly assumed, it has never been so convincingly demonstrated. The high quality of the data and assessments over many years and multiple sites inspire confidence that these finding are robust.

Some question that I hope the authors will address:
Q1: The actuarial analysis – only possible with a large study cohort - demonstrate the potential of well-powered model organism studies to shed new light on genetic regulation of lifespan. Specifically, the age-dependent effects of many/most of these loci. What happens to the allele frequencies at the loci with the largest effect sizes over time? One would expect that the frequency of short-lived alleles should decline, which would reduce power to detect these loci later in life, but it is not clear what happens to the estimates of effect sizes, aside from wider error bars. Can the authors make the case that the estimates remain unbiased? This would be important to support their interpretation of effects that changes with age.

Q2: The pattern of high male-specific mortality in early adulthood has been previously reported (PMCID: PMC6516160). This is an important finding that largely explains the sex-specific differences in lifespan. The author should cite and relate the present findings to this (and other?) reports on this phenomenon. Does it also apply to humans?

Q3: How can the allelic state of a genetic variant which is present and constant from birth throughout lifespan, influence lifespan only later in life but not early. I think I get it, but it would be helpful if the authors spent a little more text addressing this point.

Q4. The relationship between body weight and lifespan is well established (big dogs die young). The sex-specific and age-dependent nature of the effect has also been described, e.g., in work by R Miller and colleagues. What we learn here is a little different. The presence of genetic loci that affect the age-specific body weight-lifespan correlation suggest that the population level phenomenon is variable across individuals. The authors seem to imply that the mapped loci are the “cause” of the effect, but I think this is not accurate. They have demonstrated that at the individual-level, it is genetically modifiable. Comments?

Q5. The authors report a remarkably large number of epistatic interactions. The method of error rate control (referenced in Results) seems rigorous. I find this number to be implausible. I concede that this level of epistasis is a consistent with the

genetic complexity of lifespan, but how can this many effects sizes all be large enough to detect? I may have overlooked it but did not see a section describing the epistasis test to Methods.

Q6. Similar to my point above (Q4), there is a problem with the claim that genetic variants are causal for effects such as antagonistic pleiotropy and senescence (e.g. Discussion p. 21). It is more accurate to state the genetic variants are modifiers of these processes. It is, for example, plausible that genes associated with the mechanistic cause of antagonistic pleiotropy are fixed (and hence not amenable to genetic mapping), but that the process itself is modified by variable loci that are not directly causal. One way to think about it is that genetic effects are always relative. If an allele at a locus is associated with increased lifespan in early life and decreased lifespan in later life, then the complementary allele(s) should have the mirror image effect. These are difficult concepts (for me) but I would say that the genetic loci modify the effects of antagonistic pleiotropy at an individual level, not that they are the cause of.

Some minor points:
Regarding the genetic diversity of UMHET3 mice. If we use nucleotide heterozygosity – s standard measure of genetic diversity – my back of the envelope suggest that the mice are about half as diverse as (European) humans. Not a big deal but be careful about these claims.

The number of genetic loci detected for a genetically complex trait is arbitrary and largely dependent on power and error-rate control. It is safe to say that there are many more active loci (turtles all the way down). It is much more interesting in the context to focus on the effect sizes of loci, as the authors do. Some minor adjustments to emphasis on number of loci versus effect size could improve the presentation. I like the images showing how allele effects change through time. What does it look like if you take a cross section of effect sizes thought single time points?

The figure panels, especially in the supplement, as well the (main text) tables are small and hard to read. Not sure what can be done to address this.

(Remarks on code availability)
I did not review the code. The Code Ocean link was hard to navigate. The problem was likely me. That said, I have tremendous respect for some of these authors in regards to their ability to perform rigorous and reproducible analyses and expect that there would be no issues.

Referee #2

(Remarks to the Author)
The manuscript by Arends and colleagues describes the mapping of genetic loci with survival in a large number of genetically diverse mice from the ITP cohort. The authors identified multiple loci contributing to survival of which some in an age, sex or body weight-dependent manner. In addition, the show that many of these loci show epistatic effects. They performed an impressive amount of analyses on the genetic data, but I have some concerns regarding the implications of the findings. Moreover, the performed functional characterisation seems sloppy and does currently not bring much added value to the manuscript.

- The main concern I have with the manuscript is that it is unclear what the added value would be for the scientific community outside of people working on ageing in mice. There is currently no description of its implication for human ageing/health, nor do the functional analyses provide enough depth to pinpoint the biological mechanisms underlying the observed associations. Hence, I feel it is an interesting starting point for future studies, but the dataset by itself is currently of limited usability for researchers from different fields.

- The authors mentioned the current study is an extension of the study they published in 2022. However, it is unclear if they actually managed to replicate the findings from this previous study (e.g. is the locus on chromosome 12 overlapping with the one they identified in the previous study). If not, this would indicate that increasing the sample size further will not only lead to a larger resolution (which is clearly needed for more targeted functional characterisation), but may also show that some of the current findings are not replicable.

- The functional follow-up of genes in C. elegans is an interesting addition to the paper, but the way it is currently presented is sloppy and of limited value. First of all the Methods section and Figure legend do not provide any information regarding the number of independent replicates of the lifespan experiments nor the statistical tests used to analyse the data (including adjustment for multiple testing). Second, it is unclear why the authors did not perform conventional survival analyses (e.g. a logrank test) to analyse their data, but instead use AUCs. The lack of association of their positive control (daf-2) with lifespan in adult life is also a major concern. Another concern I have is that the analysed genes are just a very small snapshot of the genes underlying the linkage peak (i.e. 15/127) and it is unclear what this selection is based on (i.e. where this all available orthologs?). The Methods section states 6 orthologs although the results of 15 orthologs are shown in the manuscript. The lack of discussion regarding the biological interpretation of the majority of the observed associations (i.e. pes-4, pho-6, and dpf-5) does also not help.

Minor comments

- Instead of basing the name of the loci on just the chromosome they should be based on the regions of the genome they are located, e.g. Chr3p21.1-3q22.2, as is normally done in human linkage studies.

- The provided tables are too information dense and very hard to read. Moreover, the majority of the figures are not self-explanatory and some of the panels are unreadable. In general, I feel the large number of presented analyses makes the results section a bit difficult to digest, especially for scientist not familiar with mouse genetics.

- The authors mentioned they identified several associations in line with different evolutionary theories of ageing. However, it is unclear what the implications of this are. Does this mean that all these theories are (partly) accurate and can co-exists or is there an (unidentified) overarching theory that would match with all observed findings?

- The selection of the 15-day steps seems a bit arbitrary. The authors should explain how they came up with this number instead of using a more directly interpretable threshold like one month.

(Remarks on code availability)
There is separate code provided via a GitHub link, but I have only reviewed the models provided in the Methods section, which look fine. However, as already stated in the 'Comments for Author' section the manuscript is missing details regarding the statistical analyses of the C. elegans data.

Referee #3

(Remarks to the Author)
This is a strong and timely study that identifies and explores genes responsible for survival in mice. I particularly like (a) the care with which the significance of the findings is discussed, (b) the clarity with which the analysis is laid out. The comments below are, however, important in explaining the results, their application, and their limits.

Page 4, last para, the term "truncation age" is used differently than in demography/actuarial science. There, late truncation is assumed. Here it's early truncation. The difference was clearly explained in the previous paper (Bou Sleiman, M. et al. 2022) but should be explained here as well. This is IMPORTANT also because the genes here do NOT affect early mortality.

Page 4, last para, "striking sex difference in early ..." should be "striking sex difference in LATE ..." at least based on the figure.

Fig 1 a: the normalization used is bit odd -- or at least, hard to follow. The graph separation of F+ is also hard to follow. It IS clear that females do better at old ages, but the figure does not make it easy -- the text does.

The discussion of heritability is generally well done. But the authors should emphasize that (a) this is wide-sense heritability, (b) even though they come up with estimates like 40%, these cannot imply strong selection, (c) what do their estimates mean for further genetic studies/for evolution.

The discussion of Soma and Mass genes and their overlap with Vita genes sheds light on theories of the comolexity of metabolic scaling (e.g., Does death drive the scaling of life? Douglas S. Glazier, Biological Reviews 2024). Also, do the findings here say (or be relevant to) caloric restriction.

I sort of follow the claim (page 22) that the results here suggest that "all ITP drugs can now be placed safely within a known constellation ..." But surely the claim as written is far too strong, and must be redone.

Finally, this study while impressive does not immediately tell us what happens with humans, although it certainly suggests important ways to proceed. In light of evolutionary theories, it would be useful to summarize conclusions about the age of action of particular genes.

Version 1:

Reviewer comments:

Referee #1

(Remarks to the Author)
Arends et al examine the time- (and sex- and body weight-) depenednet genetic archictecture of lifespan in a large cohort of genetically heterogeneous mice. They identify and characterize ~60 significant genetic loci. The scale of the study and and the combination of actuarial and genetic mapping analysis are novel and should be adopted more widely. I have no serious concerns about the data, statistical methods or conclusions. The findings are complex, which is perhaps the main point, and this somewhat compromises the clarity of the presentation - e.g., large and complex summary tables. Overall the manuscript is well written. Responses to the initial review were satisfactory and I have no additional concerns to raise.

(Remarks on code availability)
I confirmed that the code is accessible on GitHub. However, the link in the manuscript breaks to due to a minor typo - easily fixed.

Referee #2

(Remarks to the Author)
The authors have done an excellent job addressing my comments and have improved the clarity and readability of the manuscript. However, I still have some remaining comments.

- The addition of the Mendelian Randomization analyses is interesting, but currently not done using the most relevant data. First of all, parental age at death is an inferior proxy for longevity in humans. Instead the authors should make use of the results from the largest GWAS on longevity for their Mendelian Randomization analyses (PMID: 33893282). Second, it is unclear why they made use of eQTL data while there is Olink proteomics data available within the UK Biobank so they could look at proteins instead of transcript levels, which is likely more informative. Hence, the analyses should be updated accordingly.

- On line 1074 the authors refer to Supplementary Table 16, but I think this should be Supplementary Table 14 instead.

Referee #3

(Remarks to the Author)
Given that this is a revision, I will only say that the authors have done a good job of responding to the referees of the original version.

Most of the comments and the responses fall into one of the following categories:
1) Clarity of presentation, analysis
2) Details of the stat methods used esp. in comparison with previous work
3) Relationship to evolution, to humans

Re 1 & 2: The authors have provided detailed explanations of what they do, what they can do, and why. The revised/extended text lays this out with admirable brevity.

Re 3: Again, the authors have done what they can, and have explained much more precisely the limitations of their results.

Version 3:

Reviewer comments:

Referee #2

(Remarks to the Author)
The authors have sufficiently addressed my remaining comments and I have no further comments.

Open Access This Peer Review File is licensed under a Creative Commons Attribution 4.0 International License, which permits use, sharing, adaptation, distribution and reproduction in any medium or format, as long as you give appropriate credit to the original author(s) and the source, provide a link to the Creative Commons license, and indicate if changes were made.
In cases where reviewers are anonymous, credit should be given to 'Anonymous Referee' and the source.
The images or other third party material in this Peer Review File are included in the article's Creative Commons license, unless indicated otherwise in a credit line to the material. If material is not included in the article's Creative Commons license and your intended use is not permitted by statutory regulation or exceeds the permitted use, you will need to obtain permission directly from the copyright holder.
To view a copy of this license, visit https://creativecommons.org/licenses/by/4.0/

Revisions to Nature MS # 2025-08-20494

To: Editor
Date: 24Nov2025
From: RW Williams, D Arends, and colleagues

We thank the three referees for their criticisms and comments. We list our responses and the changes we have made in this revision in responses to the questions and problems that the three referees forwarded to us. The comments of the referees are given in standard font and our responses are given in bold italic text below.

Rob, Danny, and
on behalf of all authors

Referee #1 (Remarks to the Author):

Arends and coauthors report genetic mapping of lifespan and body weight in a large cohort (>6000) of genetically heterogeneous mice. They employ a novel (to this field) actuarial approach that enables the characterization of variation in the genetic effect size and direction across the lifespan. Using this approach they map 29 lifespan loci (Vita) that show a high degree of age- and sex-specific heterogeneity. Using another novel analysis strategy, the authors map 30 loci that modify the age-specific correlation between body weight and lifespan (Soma). They further report ~300 epistatic interactions among the Vita and Soma loci which underscores the complexity of the genetic regulation of lifespan.

These finding, based on a well powered study design, demonstrate that the genetic contributions to lifespan, direct and indirectly through body weight effects, involve many active loci with age, sex and context specific effects. While this has been commonly assumed, it has never been so convincingly demonstrated. The high quality of the data and assessments over many years and multiple sites inspire confidence that these finding are robust.

Some question that I hope the authors will address:

Ref 1, Q1: The actuarial analysis – only possible with a large study cohort - demonstrate the potential of well-powered model organism studies to shed new light on genetic regulation of lifespan. Specifically, the age-dependent effects of many/most of these loci. What happens to the allele frequencies at the loci with the largest effect sizes over time? One would expect that the frequency of short-lived alleles should decline, which would reduce power to detect these loci later in life, but it is not clear what happens to the estimates of effect sizes, aside from wider error bars. Can the authors make the case that the estimates remain unbiased? This would be important to support their interpretation of effects that changes with age.

RESPONSE to Q1: Yes, this is correct and this is why we discontinued actuarial truncation at 1100 days when mapping. We also discontinued truncation when mapping epistatic interactions at 905 days for the same reasons—loss of power and balance of N per genotype classes. It is both a matter of loss of balance among genotypes that you mention above and the loss of sample size.

Action taken: We have revamped Supplementary Table 2. This table (set of tables) now includes sample sizes per genotype in each survivorship for all **Vita** ***loci by sex. (The original version had only the number of deaths per genotype per 15-day interval. We have also reviewed the balance of the four genotypes in loci that have late effects (≥860 days). The genotypes are well represented. For example,*** **Vita9b** ***that we dissect in Fig. 6 is represented by between 35 and 68 male mice and between 37 and 62 female mice per genotype even as late as 1100 days. Similarly,*** **Vita5a** ***and Vita10a that were both mapped late in life in males have minimum Ns greater than 42 per genotype class. We have added text on this issue in the Results section (see “*****Late-acting *Vita* loci have effects after T_{860}**”).

Ref 1, Q2: The pattern of high male-specific mortality in early adulthood has been previously reported (PMCID: PMC6516160). This is an important finding that largely explains the sex-specific differences in lifespan. The author should cite and relate the present findings to this (and other?) reports on this phenomenon. Does it also apply to humans?

RESPONSE to Q2: We agree. The reviewer is referring to Cheng et al. (2019), work that was led by one of our co-author, Jim Nelson.

Action 1. While we did cite this paper is the initial submission we did so much too discretely in the Results (Ref. 43, third line of text on the **Soma** ***loci). This point is worth emphasis and we have therefore modified paragraph 3 of the Discussion to cite Cheng et al. (2019) in a more accessible context. For the reviewer's interest we are now mapping subsets of loci that contribute to early male mortality to address whether gene variants in these loci are linked to stress responses as suggested by Razzoli and colleagues (2018, Ref. 37).***

Action 2. We have reordered the presentation on sex differences (Fig. 3). The first paragraph has an explicit comparison of sex differences between mice and humans in late mortality. Unlike humans, male UM-HET3 mice do surprisingly well—at least as well as females. In contrast, whether or not early male mortality in mice share common causes with those in humans would be speculative. Once we are able to define variants in **Vita1c, Vita6b, Vita11c,** ***and*** **Vita15b*****, that contribute to early male mortality in UM-HET3 males we may be able to test human relevance.***

Ref 1, Q3: How can the allelic state of a genetic variant which is present and constant

from birth throughout lifespan, influence lifespan only later in life but not early. I think I get it, but it would be helpful if the authors spent a little more text addressing this point.

RESPONSE to Q3: We address this interesting question of late-acting gene variants in the discussion (paragraph 2) in the context of evolutionary theories of aging.

Action: In response to this suggestion, we have added text to the last paragraph of the results on the* Vita9b *locus—our poster child for this type of locus. Just a bit more background on this interesting problem: There is a literature that goes back to Haldane and Medawar on late-acting gene variants. These types variants are compatible with either antagonistic pleiotropy or the disposable soma theory. Effects of these variants presumably "emerge" only late in life because of degraded homeostatic resilience and threshold effects exposed late in life. Once causal variants in* Vita9b *genes have been defined we will be in a better position to evaluate mechanisms of age-dependence.

Ref 1, Q4. The relationship between body weight and lifespan is well established (big dogs die young). The sex-specific and age-dependent nature of the effect has also been described, e.g., in work by R Miller and colleagues. What we learn here is a little different. The presence of genetic loci that affect the age-specific body weight-lifespan correlation suggest that the population level phenomenon is variable across individuals. The authors seem to imply that the mapped loci are the "cause" of the effect, but I think this is not accurate. They have demonstrated that at the individual-level, it is genetically modifiable. Comments?

RESPONSE 1 to Q4: Interesting question that highlights the different ways research communities use and think about the term "cause". You ask whether we think that we have mapped causes that contribute to correlations between body weight and lifespan? Yes, that is what we claim in Figure 5. But each of the* Soma *loci only makes an individually small contribution to negative correlations between weight and life expectancy early in life, or to the positive correlations late in life. The overall "cause" in this case involves at least 30 independent* Soma *loci that are individual component "sub-causes". Previous work by Miller, Nelson, and others has demonstrated body weight to lifespan correlations, but they have been interested in the phenomenology or the impact of Mendelian variants—for example, variants in growth hormone associated genes. In contrast, the Soma loci contribute to natural variation in this correlation—one that we still need to reduce to mechanisms.

RESPONSE 2 to Q4: You ask whether the loci we have mapped are causes of differences in mortality rates or are they modifiers of differences in mortality rates? A quantitative geneticist considers these two terms to be "on a spectrum" but in one important way the terms are differ qualitatively. A modifier has to have a subject that it modifies—almost always a Mendelian locus. Here is the logic: We can say without any pushback that the CAG repeat

expansion in the HTT gene causes Huntington's disease. However, there are numerous other secondary loci and gene variants that* modify *the onset age of disease. These modifier variants are conditional on the presences of the CAG expansion in the HTT gene. In our study of age-dependent mortality, none of the mapping is done "conditional" on any other locus. All 29* Vita *loci and 30* Soma *loci have their own independent, albeit small, effects on mortality.

RESPONSE 3 to Q4: Your last point referred to "the individual level". Here we need to be cautious. We have not demonstrated specific effects of any locus at the individual level. None are Mendelian loci and we can only provide estimates of mean longevity for groups of mice parsed by their genotypes.

Ref 1, Q5. The authors report a remarkably large number of epistatic interactions. The method of error rate control (referenced in Results) seems rigorous. I find this number to be implausible. I concede that this level of epistasis is a consistent with the genetic complexity of lifespan, but how can this many effects sizes all be large enough to detect? I may have overlooked it but did not see a section describing the epistasis test to Methods.

RESPONSE 1 to Q5*: *This number represents interactions detected among the 59 loci that have known additive effects. These are not additional discoveries of loci. Think of it this way: There are (59 x 58)/2 or 1711 possible epistatic interactions between the 59 loci. We tested for interactions at four time points and in both sexes. When we correct for positional overlap of* Vita *and* Soma *loci, the final number of tests is 11,768 (see the first paragraph in Results on epistasis). The percentage of epistatic interactions we have detected at a stringent threshold is actually modest—only 3.3%.

Action 1: We have added a sentence to the first paragraph on epistasis in the Results section that summarizes the point above.

Ref 1, Q6. Similar to my point above (Q4), there is a problem with the claim that genetic variants are causal for effects such as antagonistic pleiotropy and senescence (e.g. Discussion p. 21). It is more accurate to state the genetic variants are modifiers of these processes. It is, for example, plausible that genes associated with the mechanistic cause of antagonistic pleiotropy are fixed (and hence not amenable to genetic mapping), but that the process itself is modified by variable loci that are not directly causal. One way to think about it is that genetic effects are always relative. If an allele at a locus is associated with increased lifespan in early life and decreased lifespan in later life, then the complementary allele(s) should have the mirror image effect. These are difficult concepts (for me) but I would say that the genetic loci modify the effects of antagonistic pleiotropy at an individual level, not that they are the cause of.

***RESPONSE to Q6*:**
Action 1: As mentioned above (response to Q4) we are uncomfortable using the word "modifier" since this begs the question as to what exactly is being

modified. But we are comfortable finessing this issue by substituting the word "modulators" for "causes". We have made this replacement in the first sentence of the second paragraph on the Soma ***loci:*** *"*To identify genetic modulators of negative and positive weight-to-lifespan covariation…".

Ref 1, Minor Point 1. Regarding the genetic diversity of UMHET3 mice. If we use nucleotide heterozygosity – a standard measure of genetic diversity – my back of the envelope suggest that the mice are about half as diverse as (European) humans. Not a big deal but be careful about these claims.

RESPONSE to Point 1: The diversity comparison that is most germane in our context is of common variants that segregate with minor allele frequencies of great than 1% or 5%, and that are mappable in GWA studies. At a 5% MAF threshold that is used in many GWA studies, the West Eurasian ancestry has on the order of 8–10 million variants whereas the West African ancestry has on the order of 10–12 million variants (for context see 1000 Genome Project, gnomAD, and UK Biobank). In the UM-HET3 about 11 million variants segregate at a MAF of 25%. Human ancestries do not have this level of common variation. For example, highly diverse West Africans ancestries collectively segregated for about 6 million at a MAF greater than 10%.

Action: We have qualified the text in the introduction. Let us know if this new text is well tempered. It now reads: "These UM-HET3 siblings segregate for ~11 million DNA variants with a minor allele frequency of 0.25—a level of genetic heterogeneity comparable to that of the most diverse human ancestries."

Ref 1, Minor Point 2. The number of genetic loci detected for a genetically complex trait is arbitrary and largely dependent on power and error-rate control. It is safe to say that there are many more active loci (turtles all the way down). It is much more interesting in the context to focus on the effect sizes of loci, as the authors do. Some minor adjustments to emphasis on number of loci versus effect size could improve the presentation. I like the images showing how allele effects change through time. What does it look like if you take a cross section of effect sizes thought single time points?

RESPONSE to Point 2:
Agreed. This would certainly be true in a GWAS and seems true given the present results compared to those in our previous study (Bou Sleiman et al. 2022) is which we only detected one locus at a stringent threshold and six more at a lenient threshold (FDR of ~10%). With a 2-fold increase in sample size and better mapping methods we are up to 29 Vita ***loci—almost precisely the 4-fold boost expected based on predicted power. It will be interesting to see if numbers of*** Vita ***and*** Soma ***loci follow this exponential power relation as we genotype 25,000+ UM-HET3 mice or asymptote at under 100.***

Action: The question of the "cross section of effect sizes", please see Extended Data Figure 6. In this figure we provide the variance explained

across all survivorships—with the vertical axis being the "cross sections" for survivorships from 42 to 1100 days. At any one "age" (survivorship T-age) the number of loci with large effects is more modest of course than the total count.

Ref 1, Minor Point 3. The figure panels, especially in the supplement, as well the (main text) tables are small and hard to read. Not sure what can be done to address this.

RESPONSE to Point 3: Thanks, yes, we are also sensitive to this problem. We will work with Nature to ensure that all main figures are legible in the print and PDF versions. As you point out, the Extended Data figures are not easily interpretable at the default 100% magnification level. However, we have confirmed that all of these images (PDFs) are legible when magnified at 400%. They all are vector graphics and can be magnified as needed. These large "compendia" figures are necessary to enable us and all readers to conveniently review and compare effect sizes of **Vita** ***and Soma loci.***

Ref 1, Point 4 on code availability. I did not review the code. The Code Ocean link was hard to navigate. The problem was likely me. That said, I have tremendous respect for some of these authors in regards to their ability to perform rigorous and reproducible analyses and expect that there would be no issues.

RESPONSE to Point 4: Our apology. All code was and is available at *github.com/DannyArends/UM-HET3* ***(see Methods section and Additional Information section). Over the last month we have greatly improved the ReadMe file for the major classes of analysis.***

Referee #2 (Remarks to the Author):

The manuscript by Arends and colleagues describes the mapping of genetic loci with survival in a large number of genetically diverse mice from the ITP cohort. The authors identified multiple loci contributing to survival of which some in an age, sex or body weight-dependent manner. In addition, the show that many of these loci show epistatic effects. They performed an impressive amount of analyses on the genetic data, but I have some concerns regarding the implications of the findings.

Ref 2, Major Concern 1: The main concern I have with the manuscript is that it is unclear what the added value would be for the scientific community outside of people working on ageing in mice. There is currently no description of its implication for human ageing/health, nor do the functional analyses provide enough depth to pinpoint the biological mechanisms underlying the observed associations. Hence, I feel it is an interesting starting point for future studies, but the dataset by itself is currently of limited usability for researchers from different fields.

> ***RESPONSE to Concern 1: We understand the referees point very well and are also keen to get to mechanisms. In this revision we have worked hard on the last section of the results that is now titled "*****From Maps Towards Mechanisms*****". There is much more "bite" now on how our results can be used to get an mechanisms.***
>
> ***Action 1. We have also highlighted the implications for human ageing and health throughout—most prominently in the Discussion. We have also been explicit in bracketing the scope of this work by listing four crucial questions related to the causes of ageing in the Introduction that we hope will interest most readers, whatever their field. We provide specific answers—point by point—in the discussion. We think these and several other finding should interest those "outside of people working on ageing in mice".***
>
> ***Action 2 (minor but helpful). We have written a new first paragraph for the Results that outlines the main sections of the work for ease of navigation. The last section is titled*** **From Maps Towards Mechanism,** ***and its purpose is to provide concrete examples of how we and molecular biologists interested in mechanisms can exploit*** **Vita** ***and*** **Soma** ***loci to test genetic interventions and environmental perturbations using genotype-selected cohorts of UM-HET3 mice. We are doing this ourselves now using rapamycin as a perturbation (see last paragraph of the Discussion). Several new sets of sections have been added to drive home the point that loci can be used to develop mechanistic hypotheses in both the case of*** **Vita1a** ***and*** **Vita9b*****.***
>
> ***Action 3 (also minor). We provide a direct answer to the reviewer's question in a modified version of the last sentence of the introduction, that now harmonizes with the last paragraph of the discussion. This introductory sentence now reads:*** **"With persistence, it should now be practical to convert**

some of these loci into well-defined molecular and cellular mechanisms that modulate age-dependent rates of aging and risks of mortality with potential translational relevance to human health and longevity." **The last paragraph of the discussion goes into somewhat more detail on potential.**

Action 4 (a major addition). For this revision we have added an analysis of candidate genes within **Vita1a** ***and*** **Vita9b** ***using Mendelian randomization and cis-eQTL data from large GWAS cohorts in UK Biobank that included data on parental longevity. This analysis follows in the footsteps of our 2021 study in Science (Bou Sleiman et al. 2021). We cover these finding in the last section of the Results. This work involved the addition of 1. text on MR ranking of murine candidate genes for*** **Vita1a** ***and*** **Vita9b*****, 2. a new paragraph in Methods, and 3. a large set of supplementary data files on MR statistics (Supplementary Data Table 15).***

Action 5. Referee 1 also asked us to comment on the degree to which sex differences in age-dependent mortality correspond between mice and humans (see above). We added text that addresses this question. We make the point that whereas senescent male and female mice have almost precisely matched mortality rates this is not the case in humans; an interesting species difference.

Ref 2, Concern 2: The authors mentioned the current study is an extension of the study they published in 2022. However, it is unclear if they actually managed to replicate the findings from this previous study (e.g. is the locus on chromosome 12 overlapping with the one they identified in the previous study). If not, this would indicate that increasing the sample size further will not only lead to a larger resolution (which is clearly needed for more targeted functional characterization) but may also show that some of the current findings are not replicable.

RESPONSE to Concern 2: **We agree this is an important issue and worth adding.**

Action: We have added text on the replication of the mapping results from Bou Sleiman et al. 2022 into both the Introduction and Results. In brief, 6 or 7 replicate. We have also added this information into Table 1 by flagging the 6 of 7 loci that did replicate with much higher LOD scores in the enlarged cohort.

Ref 2, Concern 3 (five specific criticisms): The functional follow-up of genes in C. elegans is an interesting addition to the paper, but the way it is currently presented is sloppy and of limited value.

(1) First of all the Methods section and Figure legend do not provide any information regarding the number of independent replicates of the lifespan experiments nor the statistical tests used to analyse the data (including adjustment for multiple testing).
(2) Second, it is unclear why the authors did not perform conventional survival analyses (e.g. a logrank test) to analyse their data, but instead use AUCs.

(3) The lack of association of their positive control (daf-2) with lifespan in adult life is also a major concern.
(4) Another concern I have is that the analysed genes are just a very small snapshot of the genes underlying the linkage peak (i.e. 15/127) and it is unclear what this selection is based on (i.e. where this all available orthologs?).
(5) The Methods section states 6 orthologs although the results of 15 orthologs are shown in the manuscript. The lack of discussion regarding the biological interpretation of the majority of the observed associations (i.e. pes-4, pho-6, and dpf-5) does also not help.

RESPONSES to the five criticisms that are part of Concern 3:

Response to Point 1 on independent replicates:
Action: This information is included in Fig. 6e (far right) in which we provide an overview of the design. We used 8 technical replicates (wells) in each of 2 independent runs (plates). We agree with the referee that this information should be more visible We have highlighted this information in Fig. 6e, and we have added text to the Methods section that provide more detail on replication.

Response to Point 2 on survival vs activity assays: We opted to use activity assays rather than lifespan assays since this approach gives similar results (see citations in From Maps Towards Mechanisms). We used an age truncation method in which we computed the AUC activity levels starting from two different ages—day 0 (Fig. 6g) and day 14 (Fig. 6f). Of interest, these two AUC analyses highlight significant different knock-down effects by gene and age.

Action: We have added text in the Results and Methods that provide more context on selection of genes for testing and the use of the AUC assays. We note that log-rank tests are not generally applicable for continuous AUC data types and would require us to binarize results.

Response to Point 3 on* daf-2 *positive control: It is well known that* daf-2 *positive controls are mildly less motile early in life (please see PMIDs* 15590605 *and 9725835; and Fig 6g). Thereafter however, daf-2 knockdowns are more active relative to controls and this reflects a late-life survival advantages (Fig 6h).

Response to Point 4 on selection of orthologs that were tested: Yes, this is true; a comprehensive screen of all genes would be stronger. And yes, the ranking of candidate is mainly based on 1. missense variants, 2. a good C.e. ortholog, and 3. biological plausibility or association with longevity. For example, the Nature paper (2023, PMID:* 37612507*) from Vera Gorbunova's group motivated our including of the hyaluronoglucosaminidase genes in* Vita9b *(*Hyal1 = chhy-1*).

One other important rational for our more limited survey: Our goal at this point to highlight the potential and risks of methods of candidate gene analysis in anticipation of the next phase of this work. We are now beginning to genotype all 25,000 UM-HET3 by low-pass sequencing. We expect our loci to become much smaller or be split over the next three years. At that point the comprehensive screening of C.e. and of UM-HET3 fibroblasts will be much more practical.

Action: We have added text to the Method section that outlines our selection criteria and we have added a new Supplementary Table 14 that provides the full list of candidates and filtration variables.

Response to Point 5 on Methods section being inconsistent with the figure: Thank you for noting this problem.

Action: The Methods and the figure have been corrected. Thanks. The addition of Supplementary Table 14 should also help. Finally, we have added more biological interpretation of the two top-ranked variant in **Vita9b*****—Acad11 and Apeh—in the last two paragraphs of the Results. T***

Ref 2, Minor comment 1
Instead of basing the name of the loci on just the chromosome they should be based on the regions of the genome they are located, e.g. Chr3p21.1-3q22.2, as is normally done in human linkage studies.

RESPONSE to Minor Point 1: Nomenclature standards in mouse genetics do not follow human standards. Mouse chromosomes are all telocentric and the p and q arm cytogenetic designations are not used. We follow a short-form naming convention for loci that is relatively common in mouse QTL studies and easier to handle in tables and figures.

Ref 2, Minor comment 2
The provided tables are too information dense and very hard to read. Moreover, the majority of the figures are not self-explanatory and some of the panels are unreadable. In general, I feel the large number of presented analyses makes the results section a bit difficult to digest, especially for scientist not familiar with mouse genetics.

RESPONSE to Minor Point 2: We feel the same way and to address this problem we added a short introduction to the Results that will quickly allow readers to pick topics of most interest to them. The figures align one-to-one with the six sections of Results. Fonts have been enlarged in some figures. Yes, the two tables are dense, but this is definitely the most compact representation of the core findings of this entire study and quite readable when enlarged on screen. We will also work with the Nature team to improve legibility and clarity of these two tables and the figures.

Ref 2, Minor comment 3
The authors mentioned they identified several associations in line with different evolutionary theories of ageing. However, it is unclear what the implications of this are. Does this mean that all these theories are (partly) accurate and can co-exists or is there an (unidentified) overarching theory that would match with all observed findings?

> ***RESPONSE to Minor Point 3: Our findings are much more compatible with your first idea—that different loci support different theories of aging. We do not think our results are compatible with one overarching theory of aging. More definitive confirmation will require, as you point out, mechanistic studies of specific polymorphisms in* Vita *and* Soma *loci. We slightly expanded the discussion on these evolutionary theories of ageing to highlight implications for current major hypotheses.***

Ref 2, Minor comment 4
The selection of the 15-day steps seems a bit arbitrary. The authors should explain how they came up with this number instead of using a more directly interpretable threshold like one month.

> ***RESPONSE to Minor Point 4: Yes, we initially tried different step sizes but settled on 15-days. Larger steps obscured effects that are short lived, while shorter time spans added to computational and display overhead without exposing any novelties. The step size we can "afford" to take is an inverse function of the square root of the sample size. When we get up to x4 of the current sample we should be able to shift to 7-day intervals, and probably out to a T-age of 1200 days.***
>
> **Action: We have added text to the Methods section on the choice of 15-day actuarial steps and added more detail on the start and stop left truncation ages.**

Referee #2 (Remarks on code availability):
There is separate code provided via a GitHub link, but I have only reviewed the models provided in the Methods section, which look fine. However, as already stated in the 'Comments for Author' section the manuscript is missing details regarding the statistical analyses of the C. elegans data.

> ***RESPONSE and Action: Thanks. This is fixed in the revision (last paragraph of the Methods).***

Referee #3 (Remarks to the Author):

This is a strong and timely study that identifies and explores genes responsible for survival in mice. I particularly like (a) the care with which the significance of the findings is discussed, (b) the clarity with which the analysis is laid out. The comments below are, however, important in explaining the results, their application, and their limits.

Ref 3, Comment 1, Page 4, last para: the term "truncation age" is used differently than in demography/actuarial science. There, late truncation is assumed. Here it's early truncation. The difference was clearly explained in the previous paper (Bou Sleiman, M. et al. 2022) but should be explained here as well. This is IMPORTANT also because the genes here do NOT affect early mortality.

> ***RESPONSE to Comment 1: Yes, this is a bit confusing. In Bou Sleiman we refer to the percentage truncation level as well as fractional "exclusion levels" (see fig. 2A of Bou Sleiman et al. on the right side). By this we mean*** **conventional left truncation that only includes mice that survived up to at least a specified T-age.** ***For example, the Chr 4 locus in the Science paper that is significant at a 20% truncation level excludes mice that died at the youngest ages, but the actual age is not specified, and this obscures the biological interpretation because readers do not know what age corresponds to a specific percentage without some effort.***
>
> ***Action: Given the ambiguity of our original terminology, we have modified the Methods in two sections toward the top to improve the description and details on the method of truncation. We strictly use left truncation for all mapping presented in the paper. We now mention that other truncation methods can be applied.***

Ref 3, Comment 2, Page 4, last paragraph: "striking sex difference in early ..." should be "striking sex difference in LATE ..." at least based on the figure.

> ***RESPONSE to Comment 2. This may tie together with comment 1 and the poor explanation on our part of Fig. 1b. We have modified this paragraph and also added a better explanation in the legend. The death of many more males than females early in life (from 215 to 410 days) is reflected in the large sex difference in lifespan that include these age ranges.***

Ref 3, Comment 3, Fig. 1 a: the normalization used is bit odd -- or at least, hard to follow. The graph separation of F+ is also hard to follow. It IS clear that females do better at old ages, but the figure does not make it easy -- the text does.

> ***RESPONSE to Comment 3. We have removed the F+ regions. It is genuinely confusion and obscure the main point. The ITP team intentionally added a surplus of males into the study at weaning to compensate for early male deaths. As shown in Fig 1b, the males and females have similar lifespans but***

only after about 710 days. We have simplified Fig. 1a to finesse this problem.

Ref 3, Comment 4: The discussion of heritability is generally well done. But the authors should emphasize that (a) this is wide-sense heritability, (b) even though they come up with estimates like 40%, these cannot imply strong selection, (c) what do their estimates mean for further genetic studies/for evolution.

RESPONSE to Comment 4a on heritability. Yes, the heritability estimates include additive and dominance effects and as a result they are not narrow-sense estimates. We also provide estimates of variance accounted for by two-locus epistatic interactions between **Vita** ***loci. The relation between heritability, variant types, selection, and fitness is complex and controversial. In commenting on a draft of this submission and on this general topic, Brian Charlesworth pointed us to his 2015 paper in PNAS (Causes of natural variation in fitness: Evidence from studies of*** **Drosophila** ***populations).***

Action on Comment 4a: We have completely rewritten the paragraphs on heritability in the Results and improved the nomenclature of variance components. We have added the following text relevant to this point to the Result. "Note that the heritability Vg in Figure 2 is not a conventional narrow-sense estimate, but is the summed variance explained by the **Vita** ***loci that also will include dominance effects (Extended Data Fig. 6)."***

RESPONSE to Comment 4b on selection. We agree fully. We cannot really say anything about selection using the UM-HET3 family. This is a single sibship with no minor (rare) variants and free of most true external mortality factors that would mold age-dependent genetics of mortality. The ITP protocols do not intentionally select on any factor other than standard mouse housing.

Action on Comment 4b: None in text directly on selection, but we have added an important new paragraph in the Discussion on heritability and other sources of variance in lifespan of the many survivorships. Thanks for motivating this key addition.

RESPONSE to Comment 4c on implications of heritability for further work. This is an intriguing question that we have partly addressed in the next to last paragraph of the Discussion.

Selection for unusually long lifespans would, as you suggest, not be efficient, but because we know which genotypes and interactions are associated with longer lifespan, we could in principle genotype animals at weaning and only age those animals with polygenic longevity associated loci and combinations of loci. While interesting, we are currently focused on extending the current type of analyses to obtain much more precise estimates of parameters of age-related mortality. Once this work has been completed, we or other teams may be in a position to test the accuracy with which lifespan can be predicted.

Action on Comment 4c: Great point but no good home on the topic of selection in this paper.

Ref 3, Comment 5: The discussion of Soma and Mass genes and their overlap with *Vita* genes sheds light on theories of the complexity of metabolic scaling (e.g., Does death drive the scaling of life? Douglas S. Glazier, Biological Reviews 2024). Also, do the findings here say (or be relevant to) caloric restriction.

RESPONSE to Comment 5: **This is an intriguing review paper, and the clearest work we know of that strongly links mortality rates, metabolic rates, with other allometric trends.**

Comment 6: I sort of follow the claim (page 22) that the results here suggest that "all ITP drugs can now be placed safely within a known constellation ..." But surely the claim as written is far too strong, and must be redone.

RESPONSE to Comment 6: We have rewritten this text. The 59 loci we have defined can be used as anchor points in testing effects of drugs such as rapamycin. In preliminary work we have demonstrated strong locus-by-rapamycin interaction effects at a small subset of **Vita** ***loci.***
Action: The word "all" was too strong and has been deleted. We have rewritten this sentence to be more scientifically accurate as above.

Ref 3, Comment 7: Finally, this study while impressive does not immediately tell us what happens with humans, although it certainly suggests important ways to proceed. In light of evolutionary theories, it would be useful to summarize conclusions about the age of action of particular genes.

RESPONSE to Comment 7: All referees requested more background or implications relevant to human aging. There is now quite a bit more on this topic throughout the manuscript. Given the radical differences in life history and evolutionary history between **Mus musculus** ***and*** **Homo sapiens*****, we cannot extrapolate these granular genetic results to humans. The best we can hope for is that a subset of loci that we define in mice may also modulate mortality rates and lifespans of humans and may ultimately be actionable using interventions in both species. We are now more explicit on these point in the last two paragraphs of the discussion. Please also see our responses to:***

- ***Referee 1, Q2, on sex differences in mice and humans***
- ***Referee 1, Minor Point 1 on genetic diversity levels in the UM-HET3 and human ancestries***
- ***Referee 2 on Concern 1***

THE UNIVERSITY OF TENNESSEE
Health Science Center

College of Medicine
Department of Genetics, Genomics, and Informatics
71 S Manassas Street, Memphis, TN 38163
email: rwilliams@uthsc.edu

14Jan2026

Changes made in response to changes suggested by Referee 2 (and in response to Dr.Editor requests)

1. We have added the Mendelian randomization analysis suggested by Referee 2 using data from Deelen et al., 2019 in Nature Communication. This added further support of *APEH* as a candidate gene of late-life mortality. The –logP at *APEH* is again nominally significant using this data set that evaluated subject longevity rather than parental longevity. The necessary revisions are restricted to 1. final paragraph of Results (*From Maps Towards Mechanisms*), 2. the end of the Methods section, and 3. an improved version of Supplementary Data Table 15. The goal of MR is to step from associations toward more mechanistic models. Our thanks for the further nudge.

2. The former version of this manuscript was 7028 words, excluding the methods section. Referee 2 commented on the complexity of the results and the challenge to readers. In this revision we have managed to significantly shorten the Results, and we hope also improve the clarity. The main text is now down to 5181 words in this second revision. This loss of verbiage is a big gain for readers. Abstract is 152 words, introduction 616, results 3681, and discussion 883 words. Many typographical error have been fixed.

3. None of the changes above affect the content of the prior version or our prior responses made to address points raised by all three referees. There is one exception. In the interest of brevity, in this revision we have deleted the "orienting" paragraph that introduced the Results section. This paragraph struck us as atypical of Nature articles, but we would be happy to put it back at the start of the Results section if this is regarded as useful still by Referee 2 and the editorial team. This paragraph would add back about 160 words.

4. We have corrected errors introduced in the *From Maps Towards Mechanisms* section of the Results, specifically errors related to the potential epistatic partnerships of the *Vita1a* candidate gene with those in the other three loci. This error was flagged by two of our coauthors in the second paragraph of this section. We made the correction

and we moved most of the details into the legend (see details in Fig. 5g,h legend text).

5. We have improved some of the Extended Data figures for visual clarity (a problem flagged previously by Referee 2 with respect to small font sizes) and to be compatible with the A4 ratio. We note that some of the Extended Data figures do require magnification using PDF zoom features to see details.

6. Request to avoid language that implies causality beyond associations. We have strongly "toned down" the words "cause", "control", "direct" by extirpating them from this revision. We revisited the 04Jan2026 revision of the manuscript again and found two other instances in which the assertions were too strong. These have now been "toned down" changes are in the attached version dated 14Jan2026. We changed "direct" to "balance" in the header "Soma loci *balance* trade-offs between body weight and lifespan". In the discussion we changed "*may control rates of ageing*" to "*may influence rates of ageing*".

   We would like to push back respectfully on the complete avoidance of implications of some modest level of linkage between genotype variants and mortality risk. We could of course replace "influence" with "associate" if that is a mandate, but this is not what most geneticists mean. It is not what Mendelian randomization tries to assert. The use of "associate" nominally removes the arrow of causality but with an implied "wink". Yes, the *APOE* ε4/ε4 genotype in West Eurasians is associated with a higher risk of Alzheimer's disease by 65 years of age in large GWA studies, and there is a strongly implied arrow embedded in this statement no matter how blandly it is written. Caveats and cofactor almost always apply—sex, ancestry, age, environment, etc. The paper is written to recognize and even celebrate much of this inherent complexity. In our experimental setting we are in a comparatively strong position vis-à-vis GWASs in that environment and diet over the lifespan is relatively stable and we are working with one large sibship.

   Again, this issue is *not* a sticking point for us, but we would prefer to leave the language toned down, but not amorphous. Happy to work with Therese and the editorial team to get it right. We would also be happy to add a short caveat paragraph in the Discussion about why *Vita* and *Soma* loci are *not* good mortality predictors for individuals due to their small effect sizes (variance explained) and the high contingency on environmental factors, and many higher order terms we have not dared to measure or model yet.
